# Dual DNA Methylation Patterns in the CNS Reveal Developmentally Poised Chromatin and Monoallelic Expression of Critical Genes

**DOI:** 10.1371/journal.pone.0013843

**Published:** 2010-11-04

**Authors:** Jinhui Wang, Zuzana Valo, Chauncey W. Bowers, David D. Smith, Zheng Liu, Judith Singer-Sam

**Affiliations:** 1 Division of Biology, Beckman Research Institute, City of Hope National Medical Center, Duarte, California, United States of America; 2 Division of Computational Biology, Beckman Research Institute, City of Hope National Medical Center, Duarte, California, United States of America; 3 Division of Biostatistics, City of Hope National Medical Center, Duarte, California, United States of America; 4 Bioinformatics Core Facility, Department of Molecular Medicine, Beckman Research Institute, City of Hope National Medical Center, Duarte, California, United States of America; University Hospital Vall d'Hebron, Spain

## Abstract

As a first step towards discovery of genes expressed from only one allele in the CNS, we used a tiling array assay for DNA sequences that are both methylated and unmethylated (the MAUD assay). We analyzed regulatory regions of the entire mouse brain transcriptome, and found that approximately 10% of the genes assayed showed dual DNA methylation patterns. They include a large subset of genes that display marks of both active and silent, i.e., poised, chromatin during development, consistent with a link between differential DNA methylation and lineage-specific differentiation within the CNS. Sixty-five of the MAUD hits and 57 other genes whose function is of relevance to CNS development and/or disorders were tested for allele-specific expression in F_1_ hybrid clonal neural stem cell (NSC) lines. Eight MAUD hits and one additional gene showed such expression. They include *Lgi1*, which causes a subtype of inherited epilepsy that displays autosomal dominance with incomplete penetrance; *Gfra2,* a receptor for glial cell line-derived neurotrophic factor GDNF that has been linked to kindling epilepsy; *Unc5a*, a netrin-1 receptor important in neurodevelopment; and *Cspg4,* a membrane chondroitin sulfate proteoglycan associated with malignant melanoma and astrocytoma in human. Three of the genes, *Camk2a, Kcnc4*, and *Unc5a*, show preferential expression of the same allele in all clonal NSC lines tested. The other six genes show a stochastic pattern of monoallelic expression in some NSC lines and bi-allelic expression in others. These results support the estimate that 1–2% of genes expressed in the CNS may be subject to allelic exclusion, and demonstrate that the group includes genes implicated in major disorders of the CNS as well as neurodevelopment.

## Introduction

A number of inherited disorders of the CNS show incomplete penetrance, i.e., a high rate of discordance between identical twins, and variable phenotypes among affected individuals. The probability of transmitting these disorders to progeny is the same for both twins, so that the differences between the twins are likely to be epigenetic. Diseases showing incomplete penetrance include autism, epilepsy and multiple sclerosis, as well as a number of behavioral disorders. The inheritance pattern resembles that seen for female carriers of X-linked disorders, who are mosaics for expression of mutant and wild alleles [1], [2]. Thus, it is possible that similar monoallelic expression of a subset of autosomal genes might contribute to the incomplete penetrance of certain CNS disorders.

Despite its potential importance, there have been few studies of autosomal genes that undergo random allelic exclusion (i.e., monoallelic expression that is not dependent upon parent-of-origin). Known genes include olfactory receptors [3]; vomeronasal (pheromone) receptors [4]; some components of the immune system [5]; and a subgroup of developmental genes [6]. It has been reported that at least 1% of autosomal genes are expressed monoallelically in human lymphoblastoid cells [7], but less is known about monoallelic expression in the CNS [8].

A limiting factor in analysis of random allelic exclusion has been the difficulty of detection in mixed populations *in vivo*. Previous work has shown that regulatory DNA sequences that are both methylated and unmethylated in the same tissue could provide a first step leading to discovery of novel autosomal genes undergoing allelic exclusion.

Differential DNA methylation is known to be associated with control regions of imprinted genes (reviewed in Bird [9]). Aside from imprinting, an association of gene silencing with DNA methylation has been reported for a number of known cases of random monoallelic expression. For example, the silent alleles of the *Klra1* family genes in mouse and in human are differentially methylated [10], as is the mouse kappa light chain [11]. The monoallelically expressed cytokines IL-4, IL-5 and IL-13 require DNA methyltransferase for maintenance of gene silencing [12], and DNA methylation of the upstream region of the *Tlr4* receptor is associated with silencing as well [13].

With the advent of high-throughput screening techniques such as microarray-based methods and next-generation sequencing, it has become possible to probe DNA methylation of entire genomes or of selected genomic regions [14]–[17]. Several groups have adapted global DNA methylation screens to aid in identification of genes that are imprinted or show preferential allele-specific expression [18]–[20]. We recently described a microarray-based assay for both methylated and unmethylated DNA (the MAUD assay), and, in a pilot study, used it to identify several genes showing allele-specific expression [8].

Here we describe use of the assay to analyze the regulatory regions of the entire mouse brain transcriptome. Because rodent brain is a mixture largely of roughly equal numbers of neurons and glia [21], [22], we considered that in addition to monoallelic expression our assay might also detect dual DNA methylation patterns for genes with potential for expression in only one of these two cell types. Bioinformatic analysis of the MAUD hits and comparison with published studies on ‘poised chromatin’ in ES cells [23] suggest that this is the case. Following analysis by the MAUD assay, we identified nine genes that show monoallelic expression in some or all of the clonal neural stem cell lines. The list includes genes implicated in neural development and neurotransmission, as well as major CNS disorders. Among these is a subtype of inherited epilepsy showing incomplete penetrance, consistent with the hypothesis that monoallelic expression may in some cases lead to this pattern of inheritance.

## Results and Discussion

### Outline of the assay

**Figure 1:**
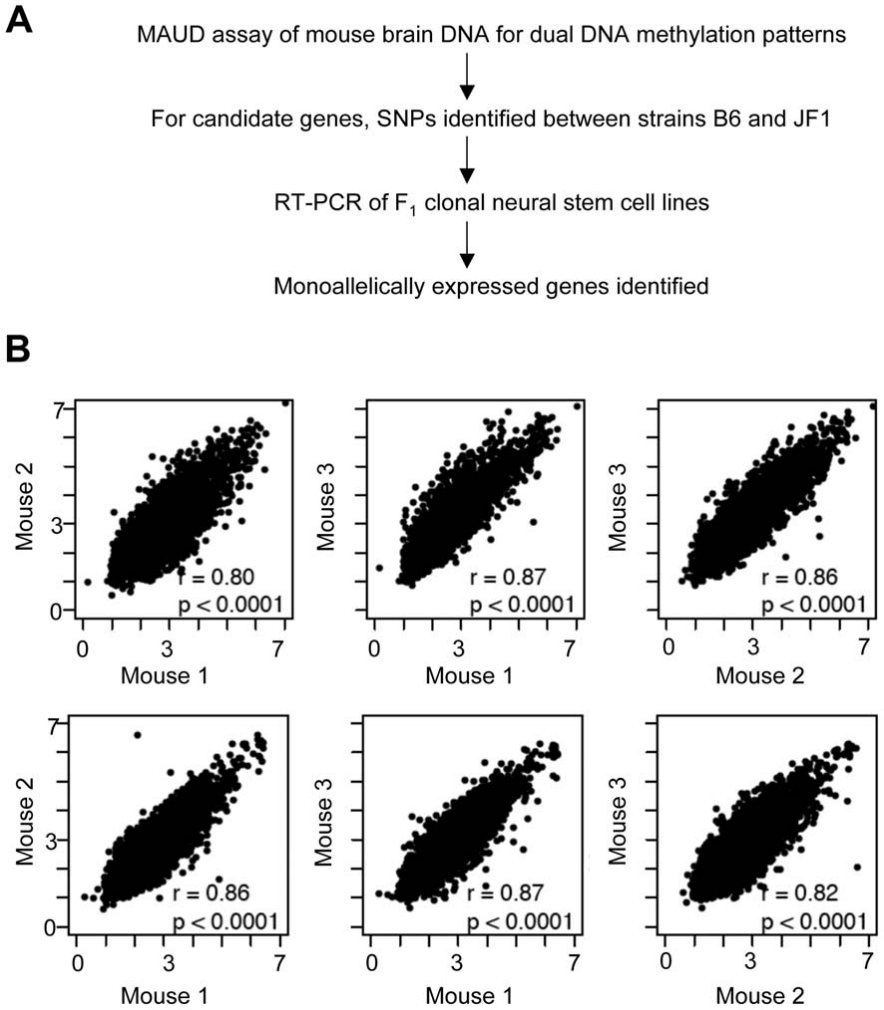
Overview of the MAUD assay. A. Experimental design. B. Reproducibility of the assay. Results for three biological replicates are shown. For each mouse forebrain sample, two hybridizations were carried out, one to detect unmethylated DNA (top row), and one to detect methylated DNA (bottom row). Each of the six plots shows the microarray signals (log2 ratio of experimental sample to control) for mouse 1 vs. mouse 2, mouse 1 vs. mouse 3 and mouse 2 vs. mouse 3 as indicated. For each combination, the correlation between mice is shown (r), as well as the two-way intra-class correlation for the presence or absence of peaks. A *P* value of<0.0001 demonstrated good reproducibility for multiple independent samples.

An outline of the experimental design is shown in Figure 1A. The MAUD assay for the detection of *m*ethylated *a*nd *u*nmethylated *D*NA has been previously described [8]. Briefly, mouse brain DNA is cleaved with a restriction enzyme (Csp6I) and oligonucleotide linkers are added. DNA is divided into three aliquots, and treated with either a) McrBC, which leaves unmethylated DNA intact, or; b) restriction enzymes (HpaII, AciI and HpyCH4IV) that leave methylated DNA intact, or, c) a mixture of all 4 enzymes, providing a negative control. Following amplification of intact DNA by ligation-mediated PCR, the methylated and unmethylated DNA aliquots are hybridized to two separate tiling arrays, each vs. the negative control. In the current study a set of 5 custom tiling microarrays was designed, containing probes for the mouse “regulome” (transcription start sites ±8 kb of 23,393 autosomal genes). Of these, 2237 genes (9.6% of the total) met our criteria for MAUD hits (Dataset S1, Dataset S3). Selected MAUD hits and other genes of interest were then probed directly for allele-specific expression in a panel of six clonal neural stem cell lines isolated from F_1_ (B6 X JF1) mice.

### Reproducibility of the MAUD assay and enrichment for monoallelic expression

**Figure 2:**
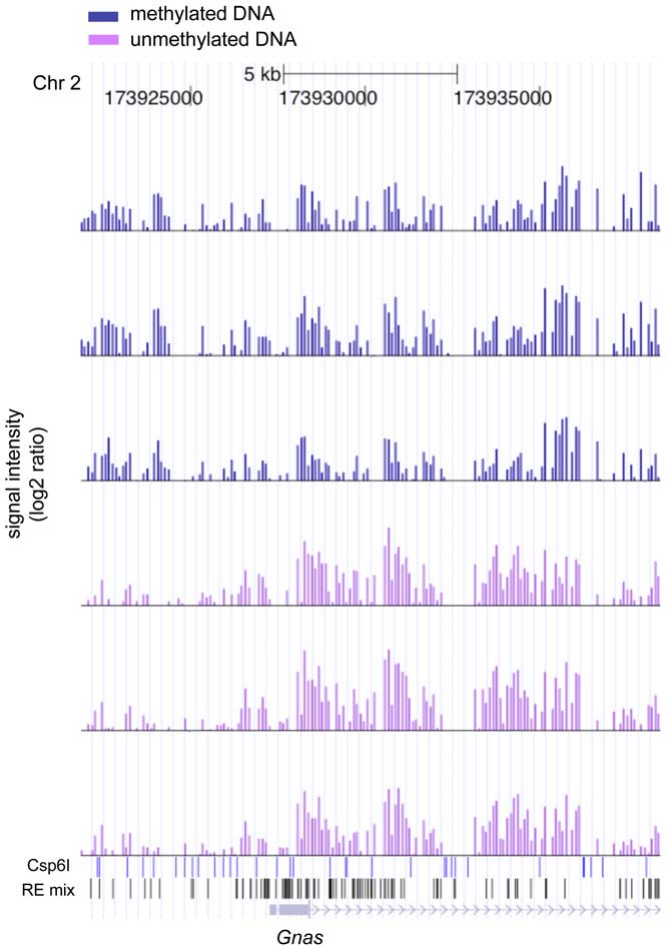
Representative results of the MAUD assay. The X-axis shows probe signals aligned with the nucleotide position and restriction sites along mouse Chr 2 at the *Gnas* locus (http://genome.ucsc.edu) [49], [50]. The Y-axis shows the log2 ratio of signal intensities for unmethylated vs. control DNA (purple bars), and methylated vs. control DNA (blue bars). The triplicate tracks show results for three biological replicates; for each track, Y_max_ is 7.2. Below the track blue vertical lines indicate Csp6I restriction sites; black vertical lines show the location of the restriction sites for HpaII AciI and HpyCH4IV. The origin of the line with blue arrows indicates the transcription start site and orientation of the differentially methylated *Gnas* gene. Nine of eleven differentially methylated control genes were detected by the MAUD assay (see text).

As shown in Figure 1B, the MAUD assay shows quite good sample-to-sample reproducibility. Furthermore, we found enrichment for differentially methylated DNA. We examined 11 genes (imprinting control centers) that are known to be differentially methylated in mouse brain at the promoter-linked sequences probed by our assay; *Gnas, Gtl2*, *H19, Mcst2*, *Mest, Nap1l5, Nnat, Peg3, Peg10 , Snrpn* and *Zrsr1*. Of these, 9 (all but *Peg10* and *Zrsr1*) showed a positive MAUD signal (Figure 2, Figure S1), reflecting about 8-fold enrichment for these regions by use of the MAUD assay *(P*<10^−6^).

**Figure 3:**
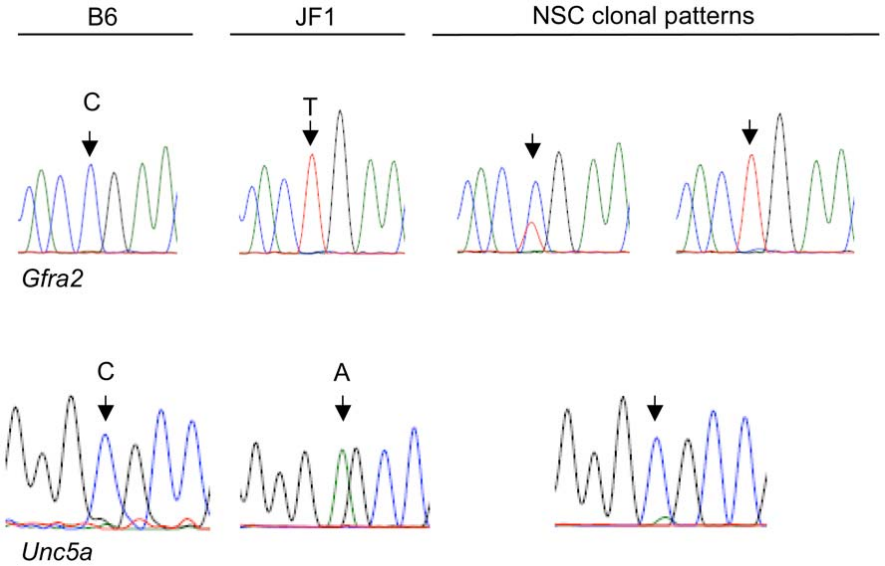
Patterns of allele-specific expression of *Gfra2* and *Unc5a* in clonal NSC lines. The chromatograms show sequencing results following RT-PCR of RNA from B6 or JF1 brain and representative F_1_ hybrid NSC clonal lines, as indicated. Complete results are shown in Figure S4, and summarized in Table 1.

**Figure 4:**
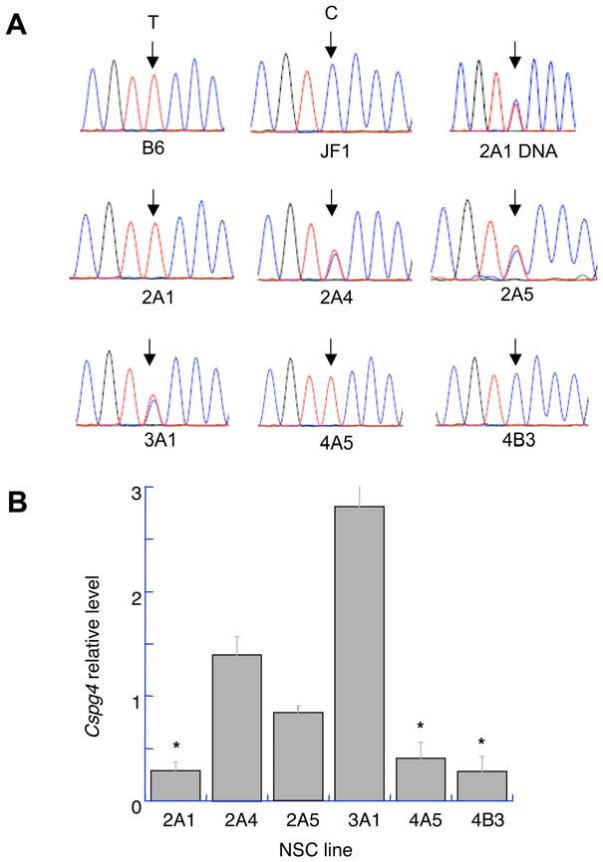
Expression of *Cspg4* in clonal NSC lines. (A). Allele-specific expression. *Top row*; SNPs between the two parental mouse strains. The chromatogram at the right shows the presence of both alleles in DNA from the NSC line 2A1. Both alleles were also present in lines 4A5 and 4B3 (Figure S4). *Middle and bottom rows:* Allele-specific expression for the clonal NSC lines indicated. (B). Relative expression in NSC lines showing bi-allelic or monoallelic expression. The latter are marked with an asterisk. Results were obtained by real-time RT-PCR. *Y-axis,* expression levels of *Cspg4* normalized relative to *Pgk1*. For each sample, error bars indicate the SEM (*n* = 3 technical replicates).

We also observed enrichment for genes showing random monoallelic expression. We analyzed the expression of 122 genes, including 65 MAUD hits and 57 other genes of interest. The genes were selected based on potential relevance to CNS development and/or disorders determined by 1) gene descriptions and 2) chromosomal mapping to disease-susceptibility loci and/or disease associations of respective human homologues. Eight of the MAUD hits, and one other gene, *Unc5a,* showed monoallelic expression in at least one of the six cell lines tested. These results are consistent with an 8-fold enrichment of genes showing random monoallelic expression, similar to that seen for imprinted loci (*P*<0.04, Fisher's exact test). Results relevant to these estimates are shown in Figure S2, Figure S3 and Figure S4, with representative data shown in Figures 3, 4 5. All genes assayed for monoallelic expression are listed in Dataset S2.

**Figure 5:**
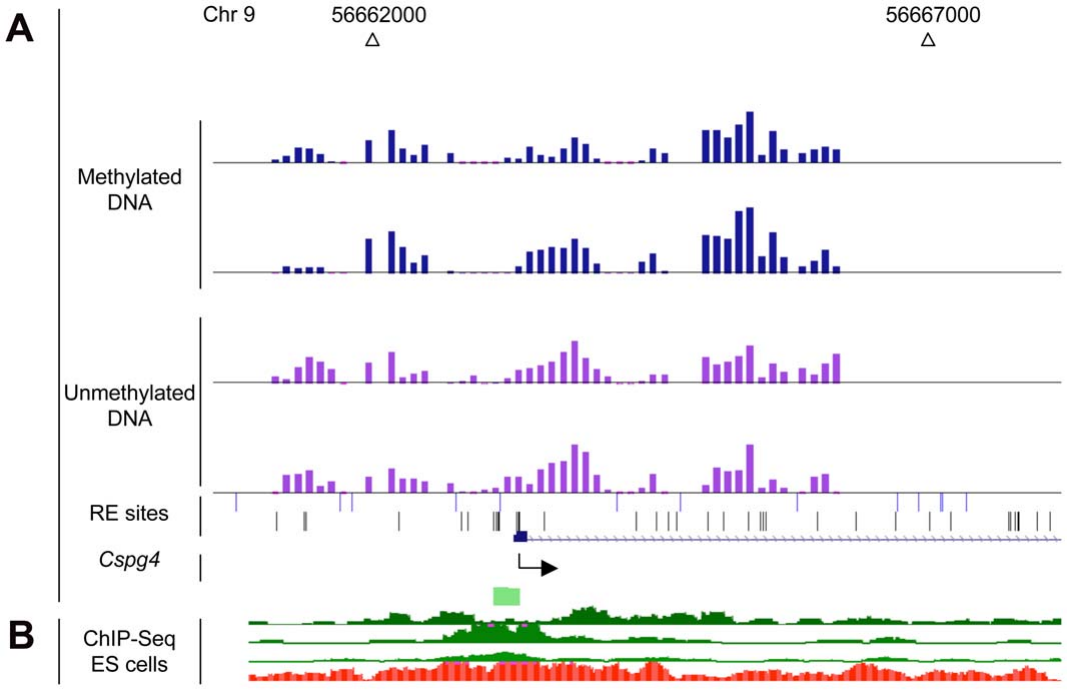
Annotation of the *Cspg4* promoter region. (A). MAUD assay results. Duplicate tracks (http://genome.ucsc.edu) show results for two mouse brain samples. The light green box shows the location of a small CpG island. (B). Location of chromatin IP signals in mouse ES cells. Top to bottom, H3K4me1, H3K4me2, H3K4me3 (green), H3K27me3 (red) [23].

### Heritable but variable monoallelic expression

The 122 genes that we probed for monoallelic expression were selected on the basis of annotation suggesting a possible role in human diseases and/or neurodevelopment (Dataset S3). Of these, Table 1 lists the nine genes showing at least 90% preferential expression of one allele in at least one NSC line. For *Camk2a, Kcnc4* and *Unc5a*, the same allele is expressed in all 6 NSC lines. For this group of genes, the preference for expression of only one of the alleles is statistically significant *(P*<0.001). While the other genes show bi-allelic expression in some (or most) of the cell lines, in those lines showing monoallelic expression there is a similar pattern of preference for either the B6 or JF1 allele (see Figure 3 and Figure S4).

**Table 1:** Allele-specific expression in undifferentiated NSCs, and NSCs differentiated to neurons and astrocytes.

Gene	Cell type	Neural stem cell line						Function/disease
		2A1	2A4	2A5	3A1	4A5	4B3	
*Camk2a*	*undiff*	91% J	80% J	81% J	97% J	84% J	91% J	Calcium/calmodulin-dependent protein kinase II
	*astrocytes* *	90% J	biallelic	87% J	90% J	biallelic	87% J	alpha/behavioral abnormalities in KO mice
	*neurons*	87% J	biallelic	biallelic	biallelic	n.d.	90%	
*Cspg4*	*undiff*	100% B	biallelic	biallelic	biallelic	100% B	100% J	Chondroitin sulfate proteoglycan 4
	*astrocytes* **	100% B	n.d.	biallelic	biallelic	100% B	100% J	/association with human melanoma, astrocytoma
	*neurons* **	95% B	n.d.	biallelic	biallelic	100% B	100% J	
*Gfra2*	*undiff*	100% J	95% J	biallelic	86% J	98% J	∧	Glial cell line derived neurotrophic factor family
	*astrocytes* **	94% J	100% J	biallelic	98% J	biallelic	∧	receptor alpha 2/kindling epilepsy association
	*neurons* **	95% J	100% J	biallelic	88% J	93% J	∧	
*Igsf3*	*undiff*	biallelic	biallelic	93% J	82% J	biallelic	biallelic	Immunoglobulin superfamily, member 3
	*astrocytes*	biallelic	biallelic	biallelic	biallelic	biallelic	biallelic	
	*neurons*	biallelic	n.d.	biallelic	biallelic	biallelic	n.d.	
*Kcnc4*	*undiff*	98% B	91% B	91% B	97% B	96% B	90% B	Potassium voltage gated channel
	*astrocytes* **	93% B	n.d.	90% B	94% B	biallelic	biallelic	/Shaw-related subfamily, member 4
	*neurons* *	n.d.	n.d.	83% B	n.d.	n.d.	93% B	
*Lgi1*	*undiff*	biallelic	biallelic	biallelic	100% J	93% J	biallelic	Leucine-rich repeat LGI family, member 1
	*astrocytes*	biallelic	biallelic	biallelic	biallelic	n.d.	n.d.	/partial epilepsy
	*neurons* **	biallelic	biallelic	biallelic	100% J	biallelic	n.d.	
*Slc6a1*	*undiff*	biallelic	biallelic	n.d.	100% B	biallelic	biallelic	Neurotransmitter transporter, GABA, member 1
	*astrocytes*	n.d.	n.d.	n.d.	n.d.	n.d.	n.d.	
	*neurons*	n.d.	n.d.	n.d.	n.d.	n.d.	n.d.	
*Unc5a*	*undiff*	90% B	92% B	92% B	99% B	88% B	94% B	Unc-5 homolog A (C. elegans)
	*astrocytes*	n.d.	n.d.	n.d.	n.d.	n.d.	n.d.	/axon navigation
	*neurons*	n.d.	n.d.	n.d.	n.d.	n.d.	n.d.	
*Vmp*	*undiff*	100% J	biallelic	98% J	100% J	94% J	98% J	Vesicular membrane protein p24
	*astrocytes*	n.d.	n.d.	n.d.	n.d.	n.d.	n.d.	
	*neurons*	n.d.	n.d.	n.d.	n.d.	n.d.	n.d.	

The letters ‘B’ and ‘J’ denote values corresponding to preferential (80% to 100%) expression of the B6 or JF1 allele, respectively. See Figure S4 for detailed results, including technical replicates and standard errors. Asterisks denote concordance with allele-specific expression in NSCs:

*P<0.05;

**P<0.0001.

∧only JF1 allele present.

*n.d*., not detectable.

Only the *Cspg4* gene showed three patterns of expression: JF1 allele, B6 allele, or bi-allelic, depending upon the cell line (see Figure 4A). Although the level of expression of each of the nine genes in NSC lines allowed measurement of allele-specific expression by RT-PCR, for most of the genes transcript abundance was in the range of one or a few molecules/cell (our unpublished data). The level of expression of *Cspg4*, (∼15 molecules per cell), was high enough for us to perform quantitative real time RT-PCR to determine relative abundance of *Cspg4* in the 6 NSC clines. Consistent with other genes showing allelic exclusion [7], we found a correlation between lower levels of abundance of *Cspg4* transcripts and monoallelic expression (Figure 4B).

Table 1 shows the patterns of allele-specific expression following differentiation of NSCs to neuronal or astrocytic populations. For most of the 6 genes showing detectable expression in the differentiated cells, the allele-specific pattern was preserved in astrocytes and/or neurons (indicated by the asterisks in Table 1). Allele-specific expression in astrocytes and neurons in culture is of particular interest considering that Table 1 also shows that nearly all of the nine genes showing monoallelic expression function in neural development/neurotransmission, and/or are associated with major diseases. For example, *Camk2a* knockouts are associated with behavioral defects [24]. *Unc5a* is a member of the immunoglobulin superfamily that is believed to play a role in cell and axonal migration in the developing CNS, acting as a netrin-1 receptor [25]. It has been reported to play a role in neuronal apoptosis during spinal cord development [26], and may influence neuronal growth in the hippocampus [27]. *Gfra2* is the glial cell line-derived neurotrophic factor (GDNF) family receptor alpha-2. It plays a role in neuron survival and differentiation. *Gfra2* and *Lgi1* have been linked to two different subtypes of epilepsy [28], [29]. *Cspg4*, a membrane-bound chondroitin sulfate proteoglycan, has been reported to play a role in melanoma spreading along the epithelium [30]. Under the alternate symbol NG2, the protein has been characterized in the brain [31]. There is a link between its up-regulation in brain and astrocytoma [32], [33].

### Monoallelic expression and incomplete penetrance

Two aspects of the allele-specific expression patterns we observe may be relevant to incomplete penetrance of a number of inherited diseases. First, is the gene-specific variability between different clonal cell lines. There is precedent for such variability in different human clonal lymphoblastoid lines [7]. There is also evidence for probabilistic regulation even within clonal populations in the immune system. For example, cytokines and NK cell receptors in an activated clonal population may be expressed from either or both alleles [34]–[36]. It has been suggested that this stochastic process is part of development of the immune response repertoire; perhaps, in some cases, a similar process is intrinsic to development of the central nervous system. In any event, our results suggest that adult tissues will be mosaics of cells expressing one or two alleles for some genes. For CNS tissue, the location and/or connectivity of cells expressing only a mutant allele in a heterozygote could determine the penetrance of the respective inherited disorder.

The second potentially relevant aspect is preferential expression from one allele. Genes showing such preferential expression may contribute to the inheritance pattern of diseases that show autosomal dominance with incomplete penetrance. For example, human *LGI1* mutations cause ‘autosomal dominant partial epilepsy with auditory features’, with penetrance varying from 25% to 100% in different affected families [29]. Such variation could be explained by a hierarchy of preferential expression of different alleles, as is seen for the X-linked *Xce* locus [37]. It might also arise from selection of a preferred allele during development [38]. A mutation in a preferentially expressed allele would result in a dominant phenotype, whereas the same mutation could result in incomplete penetrance if the respective wild type allele showed preferred expression. Recent studies demonstrating mouse strain-specific DNA methylation patterns [39], and at least two cases of allele-specific expression associated with specific SNPs in human [20] provide precedents for this idea.

### Significance of dual DNA methylation patterns in mouse brain

Previous studies have shown DNA methylation differences between brain and other organs, but dual DNA methylation patterns within brain were not examined. For example, it is known that about 5% of CpG islands show different DNA methylation patterns depending upon organ type [39]. One recent study identified a small number of “tissue-specific” differentially methylated regions that were almost entirely methylated in brain or kidney [40]. Promoter strength and CpG density seem to influence the likelihood of DNA methylation, although results obtained by different techniques are not entirely consistent [41]–[45].

Unlike these previous studies, the MAUD assay has allowed us to screen for sequences that are both fully methylated and unmethylated in the same tissue. Our finding of such sequences in brain suggests the possibility of lineage-specific methylation within brain. Since there are approximately equal numbers of glial and neuronal cells in adult rodent brain (reviewed in [22]), the MAUD assay will detect genes that are methylated in one cell type and unmethylated in the other. We therefore hypothesized that, in addition to its use to detect monoallelic expression, differential DNA methylation within mouse brain may also identify clusters of genes that have the potential for expression only in neurons or glia, but not both.

We first utilized the DAVID Bioinformatics Resource [46], [47] to assess whether MAUD hits were significantly enriched in genes that might be expressed in differentiated cells of the CNS. We found statistically significant enrichment for voltage-gated channels (*P*<10^−6^, 2.6 fold enrichment, Swiss Protein (SP) keyword), genes involved in development (e.g., system development, *P*<10^−7^, 1.4 fold enrichment, Gene Ontology term 0048731), and guanine-nucleotide releasing factors *(P*<10^−9^, 3.3 fold enrichment, SP keyword). The DAVID clustering algorithm confirmed these classes of molecules to be robust in their enrichment (Table S1). Subdividing the list of MAUD hits by distance from transcription start sites (Figure S5) did not further distinguish these enriched groups of genes.

We next examined whether there was a relationship between MAUD hits and a subset of genes analyzed by ChIP-Seq in a previous study [23]. In that study, ∼2700 of the assayed genes were found to have two opposite chromatin modifications in ES cells, H3K4Me2/Me3 *and* H3K27Me3, markers for active and silent chromatin, respectively. The finding of genes showing this bivalent (or ‘poised’) chromatin, led the authors to propose that the bivalent state would be resolved to univalency depending upon the potential for expression in various differentiated lineages.

We found that of 2,337 MAUD hits, 405 genes (18% of the total) are included in the list of genes with poised chromatin in ES cells. The enrichment is highly significant when compared to the frequency of such chromatin for all 23,393 genes we assayed *(P*<0.0001, chi-square test). An example is shown in Figure 5 (see Dataset S3 for the complete list). Use of the DAVID Bioinformatics Resource showed that the overlapping genes are even more enriched for voltage-gated channels (7.3-fold enrichment, *P*<10^−11^) and for genes expressed during development (2.4-fold enrichment, *P*<10^−18^). Genes involved in sensory organ development are particularly enriched in this overlapping set *(P*<10^−6^, 3.9 fold enrichment^,^ versus *P*<10^−2^, 1.6 fold enrichment, for overlapping and all MAUD hits, respectively). In the MAUD hits that were not bivalent by the criteria of Mikkelsen et al. [23], there was no enrichment of genes coding for ion channels or development. These results are consistent with the hypothesis that stem cell bivalency foreshadows later developmental events where different organs and/or cell types are restricted in their potential to express certain genes via DNA methylation.

### Perspective

We have used the MAUD assay to analyze differential DNA methylation at regulatory regions of the mouse brain transcriptome. Our results show that differential DNA methylation may mark cells of different lineages within brain, and suggest that the assay will be useful for similar analysis of other tissues and developmental states.

The MAUD assay enriched eight-fold for genes showing monoallelic expression. We do not yet know whether the dual DNA methylation pattern of these genes is due to two different states of DNA methylation within each cell of the CNS (as expected for imprinted genes), or is an epigenetic manifestation of the two different major lineages within the brain. Interestingly, MAUD hits and the genes showing poised chromatin in ES cells are both enriched for genes expressed during development. The overlapping genes include four of the eight MAUD hits showing monoallelic expression, raising the possibility of a link between poised chromatin during development and the potential for monoallelic expression. In any event, given the likelihood discussed above that many of the MAUD hits reveal differences in developmental commitment between neurons and glia, we expect that specific enrichment for genes showing monoallelic expression will be significantly larger than 8-fold in more homogeneous cell populations. We observe partial DNA methylation of these genes not only in the CNS, but also in kidney, liver and lung (Figure S3). These results are consistent with the potential for monoallelic expression of these genes in non-CNS tissues.

Two of the nine genes that show allelic exclusion are implicated in epilepsy, some subtypes of which show both twin discordance and phenotypic variability. Our finding of monoallelic expression of *Lgi1*, if true also in human, would provide an example of how such expression may affect specific inheritance patterns. Increased levels of *Cspg4* are associated with astrocytoma. In light of this association, it would be worth exploring the mechanism(s) underlying silencing of one allele. In summary, the compelling functions of the genes we describe here suggest that additional high-throughput screens for monoallelic expression will lead to insight concerning major disorders of the CNS, and how they are inherited.

## Materials and Methods

### Ethics statement

Procedures on mice involved little or no pain or distress and were approved by the Institutional Animal Care and Use Committee of City of Hope (IACUC #97013, approved until 12/20/2010). City of Hope is accredited by AAALAC.

Adult (∼6 week old) female C57BL/6 (B6) mice were used for the MAUD assay as previously described [8]. NSC lines were derived from F_1_ mice resulting from the reciprocal cross of strains B6 and *Mus musculus molossinus* JF1 (JF1); for cell lines 2A4 and 2A5, the JF1 allele is maternal; for cell lines 2A1, 3A1, 4A5 and 4B3, the B6 allele is maternal [8]. Differentiation of NSC lines to neurons and astrocytes was carried out for 7 days as previously described [48]. Differentiation was monitored by microscopic examination of cellular morphology, and by quantitative RT-PCR for *Nes* and *Dcx* transcripts (see Figure S4). Isolation of DNA and RNA, the MAUD assay and RT-PCR were all performed as previously described [8]. Dataset 2 lists the primers used in this study. Design of the 5-chip Nimblegen custom array was based on mouse genome build 36 (Mm8): Coordinates of all tiling array probes are available upon request.

The algorithm used for detection of MAUD peaks was previously described [8]. In the present study, an additional filter was used requiring that peaks for both unmethylated (UM) and methylated (M) DNA coincide in the same Csp6I fragment. Within each fragment, amplitudes of peaks were estimated by summing the values (log2 signal vs. background) of the respective probes. Criteria for MAUD hits included: 1) UM and M peaks were present in each of the three biological replicates; 2) The ratios of M to UM of the peak amplitudes were allowed to vary two-fold about 0.8 (a 1∶1 ratio corrected to 0.8 to reflect the M/UM ratios in known differentially methylated controls). 3) Reflecting the same correction, a minimum peak amplitude of 5 and 4 and a median probe height of at least 2 and 1.6 were required for UM and M peaks, respectively. Relevant information for MAUD hits and other genes of interest including gene descriptions, mapping coordinates (cytobands) of homologous human genes, and associated OMIM diseases were obtained from NCBI databases (www.ncbi.nlm.nih.gov).

The DAVID Bioinformatics site (http://david.abcc.ncifcrf.gov) was used as recommended to determine possible enrichment of gene categories. The background consisted of all autosomal genes (geneids) probed by the Nimblegen arrays. The relevant *P* values noted are also from the DAVID Bioinformatics website. Aside from these, and unless otherwise noted, *P* values for tests of proportions were modeled using binomial distributions. In the case of concordance between NSCs and differentiated astrocytes or neurons, a +/− 10% standard error binary classifier was used, based on the estimate that up to 10% of genes would show concordance by chance alone.

For real time RT-PCR, reverse transcription was carried out on 500 ng of total RNA mixed with random primers. PCR was performed using iQ SYBR Green Supermix (Bio-Rad). Each 25 µl reaction mix contained 1 µl (∼25 ng) cDNA and 0.4 µM of upstream and downstream primers for *Cspg4* or *Pgk1*. For absolute quantitation, a dilution series of the *Cspg4* amplicon quantified with PicoGreen (Invitrogen) was included. The number of *Cspg4* transcripts/cell was then calculated assuming ∼30 pg total RNA/cell.

## Supporting Information

Figure S1 MAUD assay of differentially methylated controls. Results for Gtl2, H19, Mcst2, Mest, Nap1l5, Nnat, Peg3 and Snrpn are shown in alphabetical order. X-axis, nucleotide position along the mouse chromosome indicated. Y-axis, log2 ratio for methylated DNA vs. control (blue bars) and unmethylated DNA vs. control (purple bars). Ymax for each track, 7.2. For each gene, results are shown for the three mice assayed. Below the six tracks, the blue vertical lines show the location of Csp6I sites, and the black lines show the location of DNA methylation-sensitive HpaII AciI and HpyCH4IV sites. Below these lines, the transcription start site and structure of each gene is shown schematically: Positions of exons (bars) and introns (small arrows) are shown, with the direction of the arrows indicating the orientation of transcription. The figures were obtained by alignment of our custom tracks with annotation showing the location of the genes and restriction enzyme sites indicated [49], [50].
(0.29 MB PDF)

Figure S2 MAUD assay of genes showing monoallelic expression. Maximum peak height (log2) ratios are shown for peaks that are coincident in both tracks. The turquoise boxes highlight the DNA sequences analyzed directly for DNA methylation (Figure S3).
(0.14 MB PDF)

Figure S3 Partial DNA methylation of selected MAUD hits. DNA methylation analysis was carried out by use of the EpiTYPER system (Sequenom). Briefly, 1 µg bisulfite treated DNA from B6 brain, kidney, liver or lung was amplified with gene-specific primers using downstream primers that contain a T7 promoter tag. Following in vitro RNA synthesis and base-specific cleavage, MALDI-TOF mass spectrometry was used to determine relative DNA methylation based on the RNA cleavage pattern. Adjacent circles show biological replicates as indicated. Control panels show B6 (brain) DNA spiked prior to bisulfite treatment with either 1 ng of unmethylated amplicons or 1 ng of SssI-treated methylated amplicons, as shown. SssI treatment was carried out following the instructions of the manufacturer (New England Biolabs). Primers were designed with the aid of Primer3 or MethPrimer (www.urogene.org/methprimer), as appropriate. A list of relevant primers is available upon request. For each gene analyzed, CpG sites (or clusters) underlined below correspond to CpG #1, 2 or 3, respectively. For CpGs included within restriction enzyme sites AciI, HpaII or HpyCH4IV, the entire restriction site is underlined. Camk2a: GCAAGACTGCGTCACAGAGCG Cspg4: GGGGCCAGCCGTCGTCCTTGAGTCAAGCCTTGAAGGGTGGGAAGGGAGTCTGACTCCTGTCTGCGGTCCTCAGCCTGGACAAGAGCAGGAGGTGGGTGTAACGGGGTGTTGAA Gfra2: CCTAGCCTCACGCTCCAAGGATGAAGCCAGACAAGTCCAAAGTATAAATAACAAAAAAGGATTTTCATTCTCATGATTCTTTTTTTCCAGACAGGGCAGAGAGAAAAGGATTATCTCAGATGTCCTTAATGCAGGCACAGAATCTACAGACCCAGAGCTGCTGTCATTTTGTTTATTCATATGCTAACCCGGATTGACTAATG Igsf3: GCAGCTGGTCGCTCGCGTCTCCATTCTAGGTTTCTTGCACTTACAGGATTTATCCGTGGAGGTTGTCTCTGAATTATCTGCACCCTTATAAAAGTTAACAGGCATCCGGAATGAGGATG Kcnc4: GCAAAACCCCGGAATTAGGATGCTTGGTGAAGAGCTGGGTTCCCCCCACCACTTTTTTATGAATTGCTTATTCCCACTTGTGTGTCCAGGAACAGCTCAGAATTGGCCTCTGCCTATGTTCCTCCGCTGTGGGCAAGTCTTTTGGCTCCTGTGCCAGCCAGAGCGCCACAGCATGA Lgi1: CCTACAGGCACGTTGGATACCCCCACCTTGTACACAGTGAATGCTGGCCCTGGTTTGCAGCAGTTTCCACTTCCATGAAGCTTTTAATCCTCTCGAATCAACATTATCACCACCACCATCATCATCCTCATCCCAGCAATCCAATAAGAGTGTGAACATGACTAACACTGCCCTCCTCTCACAAAGCCAATAGAGTTAAAGGAGCCATTGAGCCCGGAGTCAGTT Slc6a1: GGAGACAGACGGCCGGTCACCACTGAGGGAAAAAACGGCAATGATCAGTCCCCAGTGGAAACCGTGTTCTGGGG Vmp: GCTGAAGGCCGGTTCTCAATGATCAAGATCCAATTTCACATTTTCTCAATTGCTGTAGCTAAGAAATTCTGTGTGTCCAGATCTGAGGCCTAGCCTTTGTTTCACAGGGAAGCTTTACTTTGTAGAGGAACGTGGGGTGTGCTGAAGGTGTAGAAGACCAGACTGGTAGCACACTTAACCTTGATGAGGTAGAGTGTCAGGCTGCAGAGTGCTGTAATACTTACGTTGTAAATCCA .
(0.07 MB PDF)

Figure S4 RT-PCR results for genes that show allele-specific expression in NSC lines. (A-I). Results are shown for Camk2a, Cspg4, Gfra2, Igsf3, Kcnc4, Lgi1, Slc6a1, Unc5a and Vmp, in alphabetical order. Automated sequencing of RT-PCR products was performed after identification of B6/JF1 SNPs included within the amplified sequences. Representative results are shown for brain tissue from B6 and JF1 mice, F1 hybrid progeny, clonal NSC lines and neuronal and astrocytic populations derived from them, as indicated. PCR results for genomic DNA from relevant clonal lines are also shown, verifying the presence of both alleles. The relative intensity (peak height) of the signal for each base at SNP sites was measured to determine the % signal. For each sample, the percent expression of the predominant allele is shown. The number of replicates is in parentheses. For samples including at least three technical replicates, the SEM is given; otherwise the range is shown. CI, confidence interval. (J). Standard curves confirm the linearity of the assay. For each gene shown, RT-PCR products of strains B6 and JF1 were mixed in the proportions shown prior to automated sequencing (% input). Representative standard curves are shown for the mismatches C vs. C+T, T vs. C+T, A vs. A+G, and C vs. A+C, as indicated. (K). Comparison of Nes (nestin) RNA in neural stem cells vs. atrocytes/neurons (left graph) and of Dcx (doublecortin) RNA in astrocytes vs. neurons (right graph). Nestin and doublecortin are markers of neural progenitor cells in developing and adult brain, and of cells of neural lineage, respectively [51], [52]. Quantitative real-time PCR of cDNA was performed in triplicate. For each experiment, values were normalized to those obtained for the housekeeping gene Pgk1, with the average relative ratio set at 1.0. Results are shown for all cell lines except for the outliers 2A5 (left graph) and 3A1 (right graph). (Outliers showed values >2 standard deviations from the mean.) Error bars, ± SEM.
(1.10 MB PDF)

Figure S5 Distance of MAUD peaks from transcription start sites. Top, MAUD hits with monoallelic expression; bottom, all MAUD hits.
(0.02 MB PDF)

Table S1 Top 10 annotated clusters from DAVID Bioinformatics Site.
(0.12 MB DOC)

Dataset S1 List of MAUD peaks.
(0.39 MB XLS)

Dataset S2 List of primers and genes assayed.
(0.07 MB PDF)

Dataset S3 Expression pattern and description of MAUD hits.
(0.40 MB XLS)
