# Supplementary figures and images for: Dual DNA Methylation Patterns in the CNS Reveal Developmentally Poised Chromatin and Monoallelic Expression of Critical Genes
Source: PLoS One. 2010 Nov 4;5(11):e13843. doi: 10.1371/journal.pone.0013843 (PMC2973945; doi:10.1371/journal.pone.0013843)

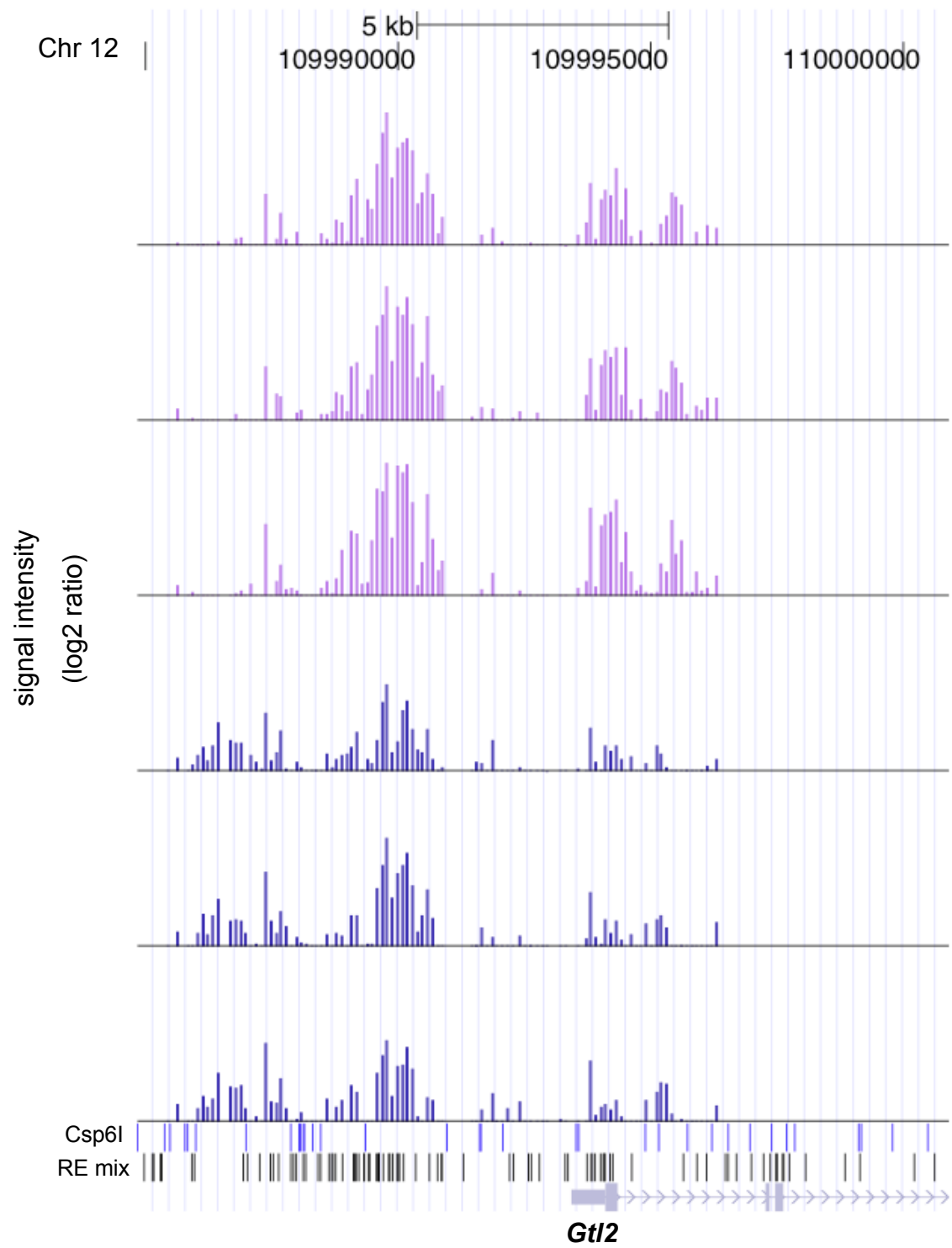

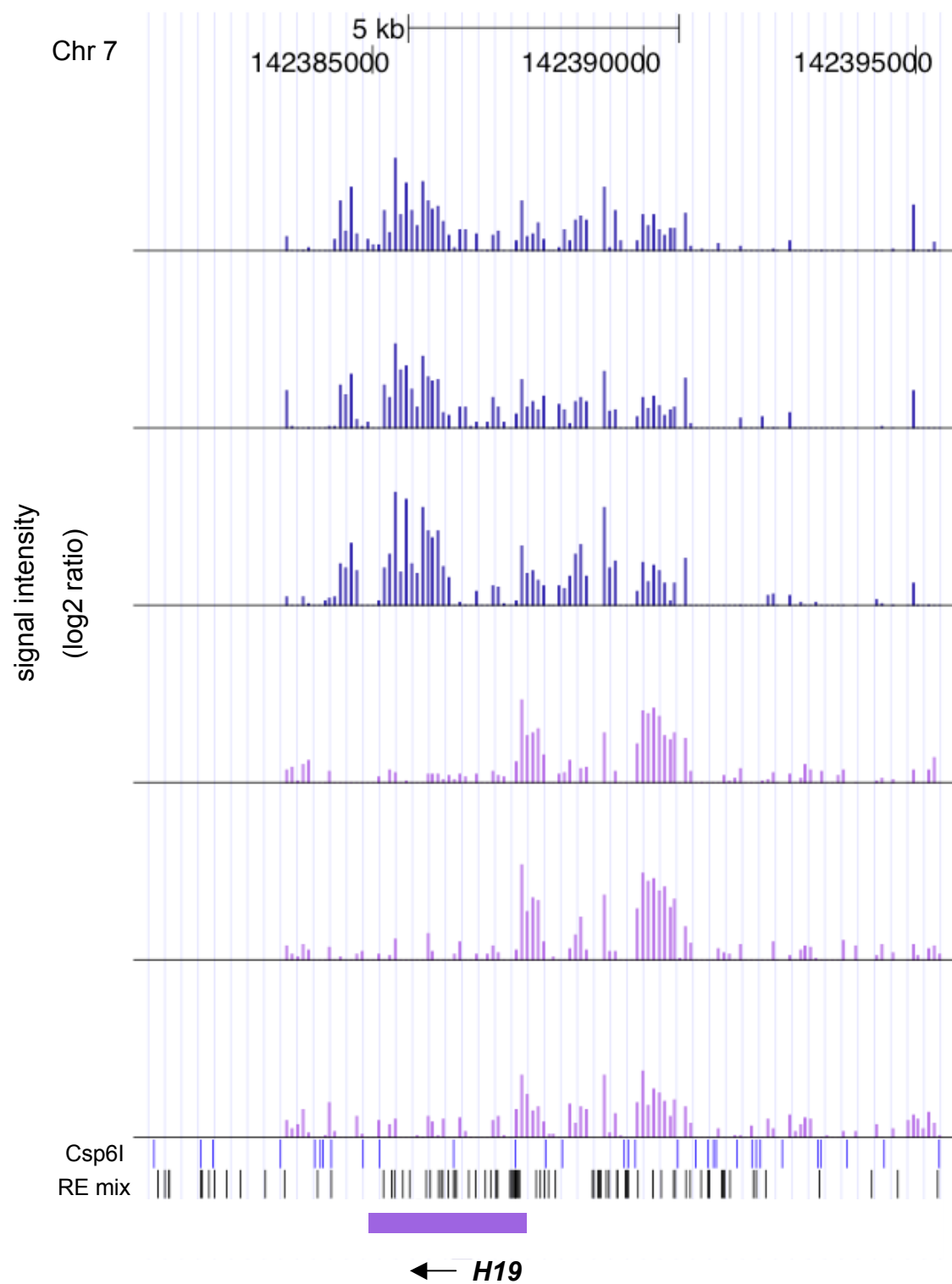

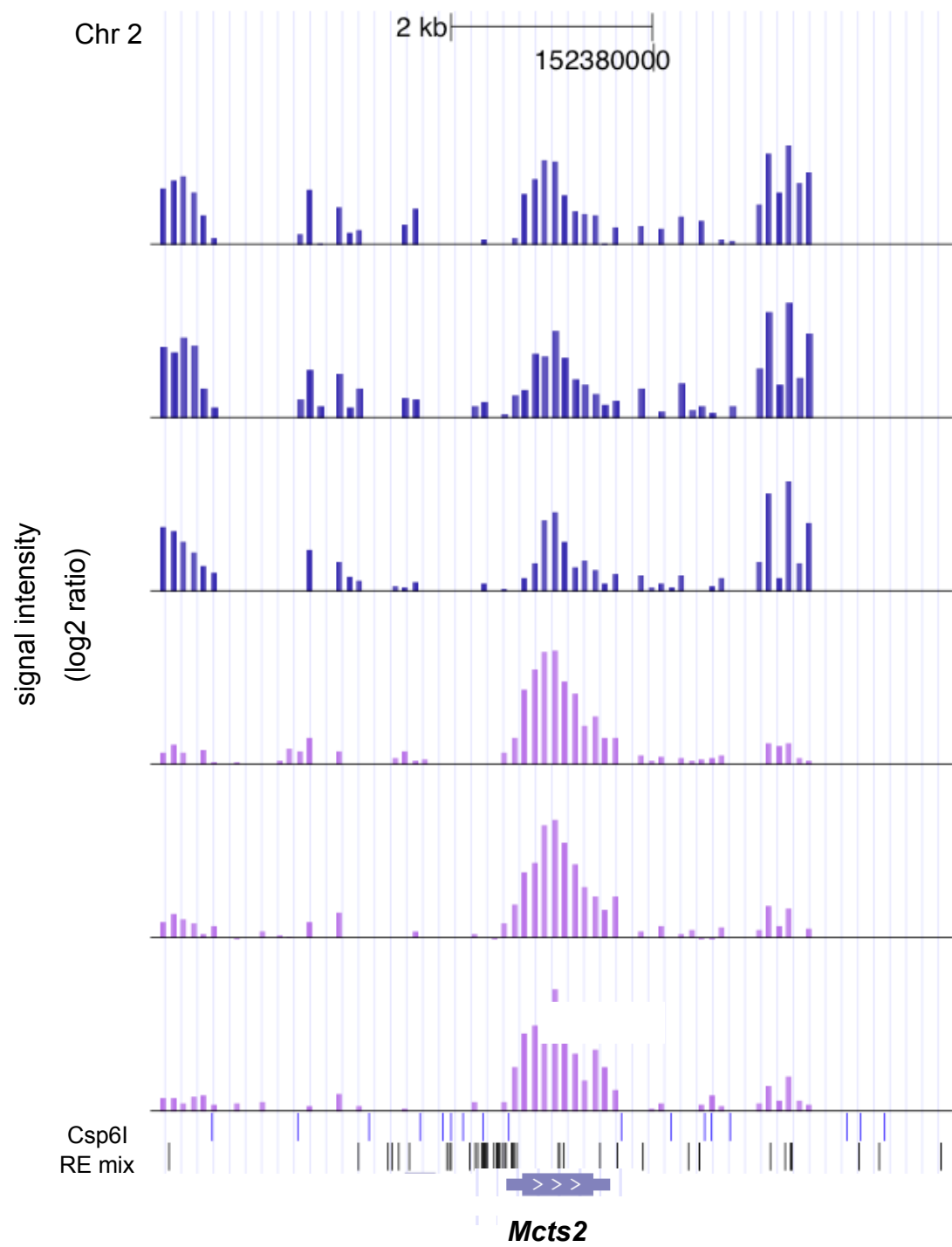

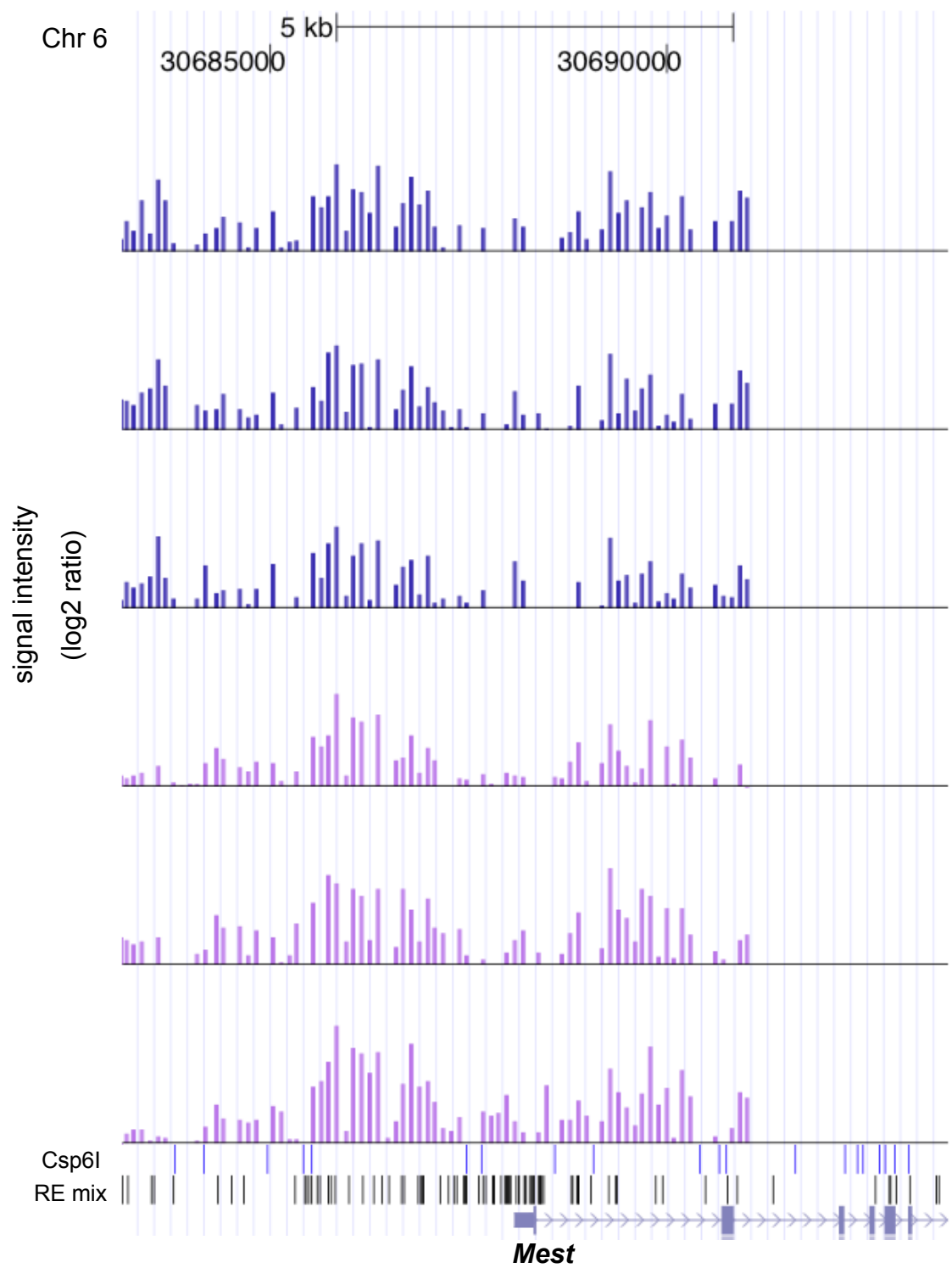

Chr 6

5 kb

58835000

58840000

signal intensity  
(log2 ratio)

Csp6l  
RE mix

*Nap1l5*

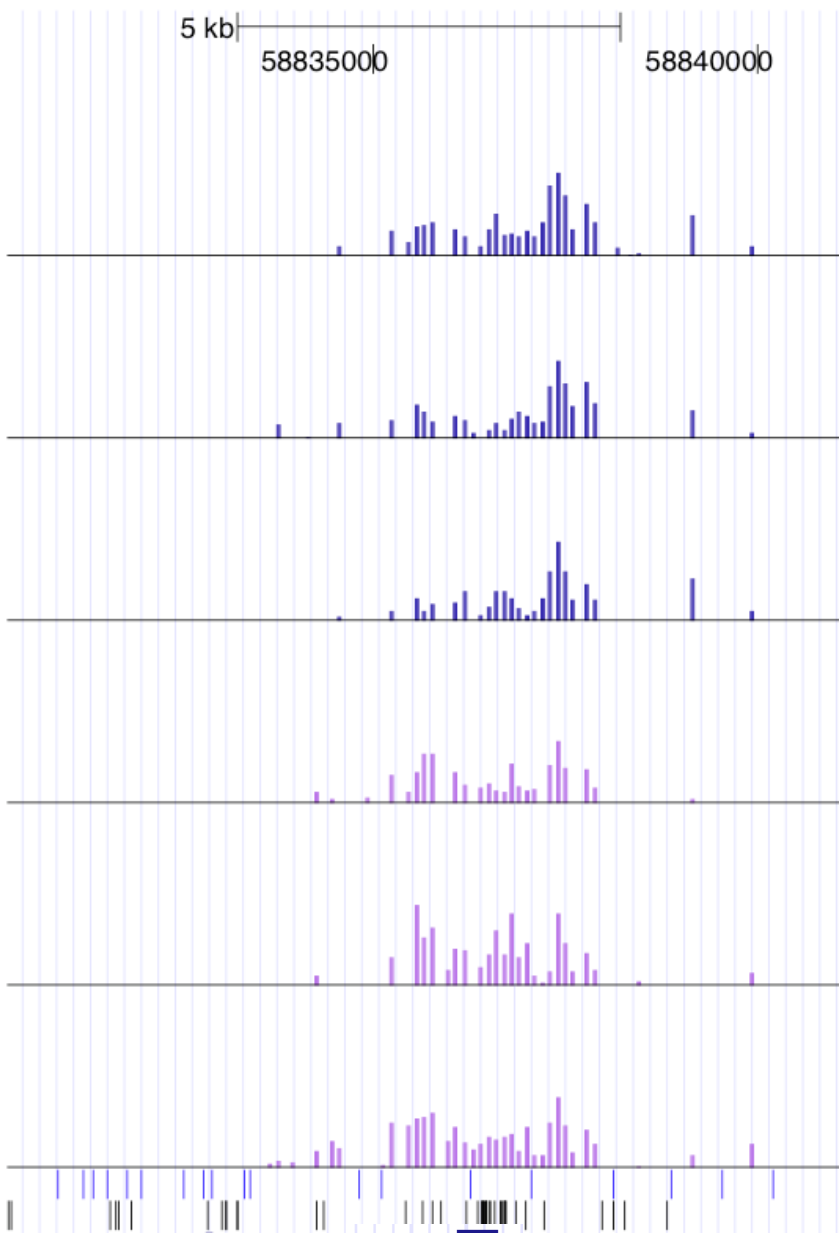

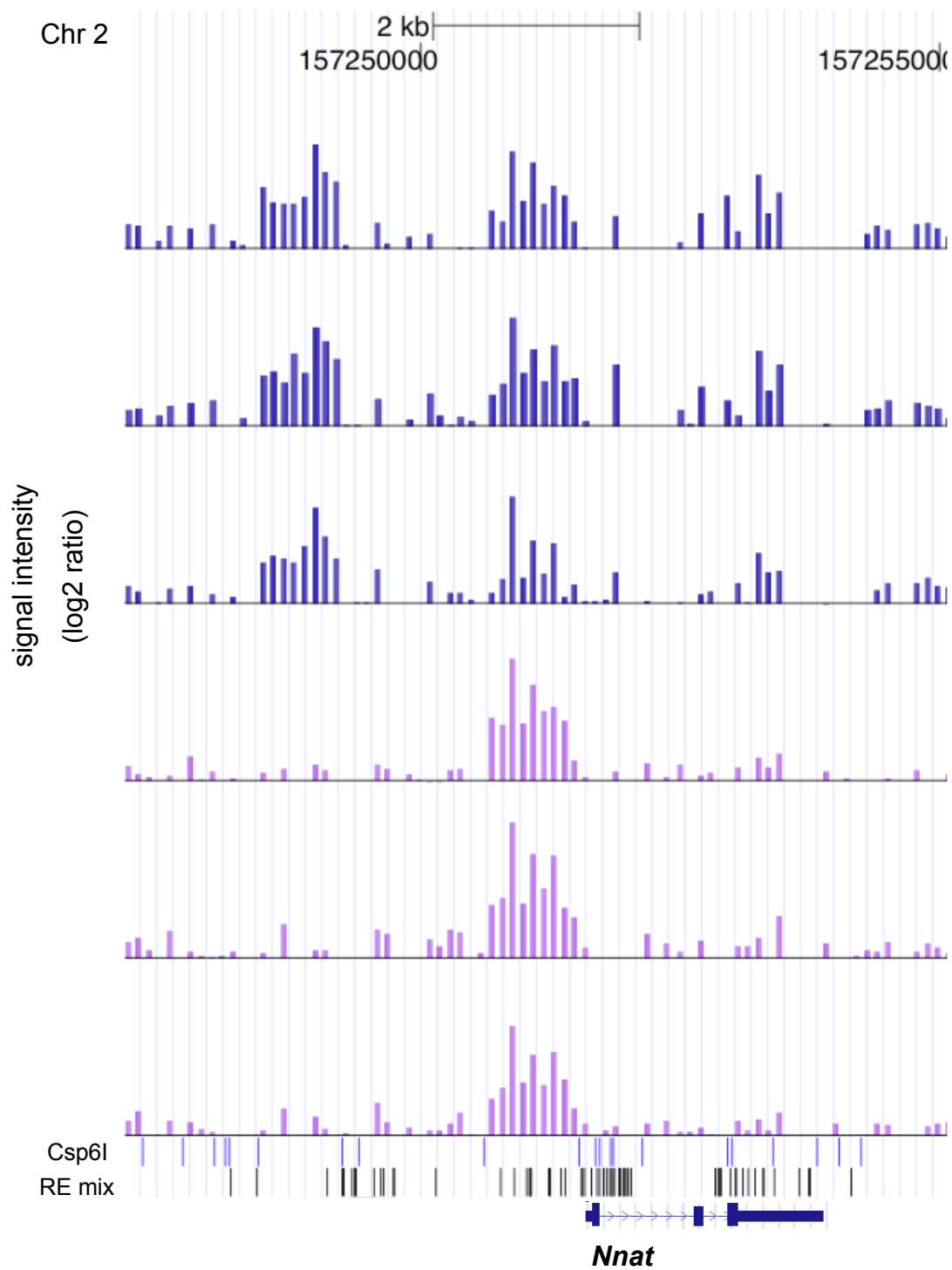

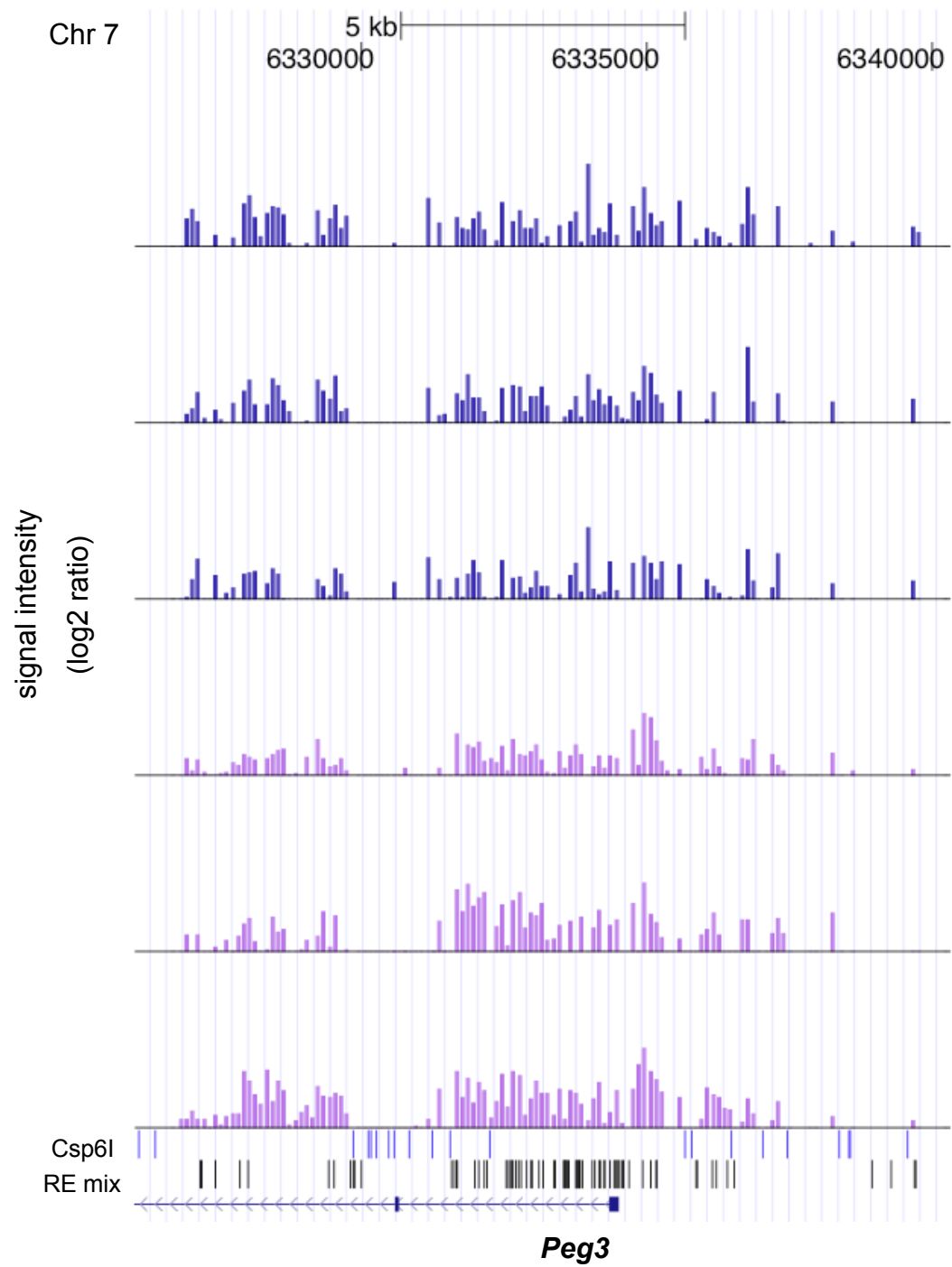

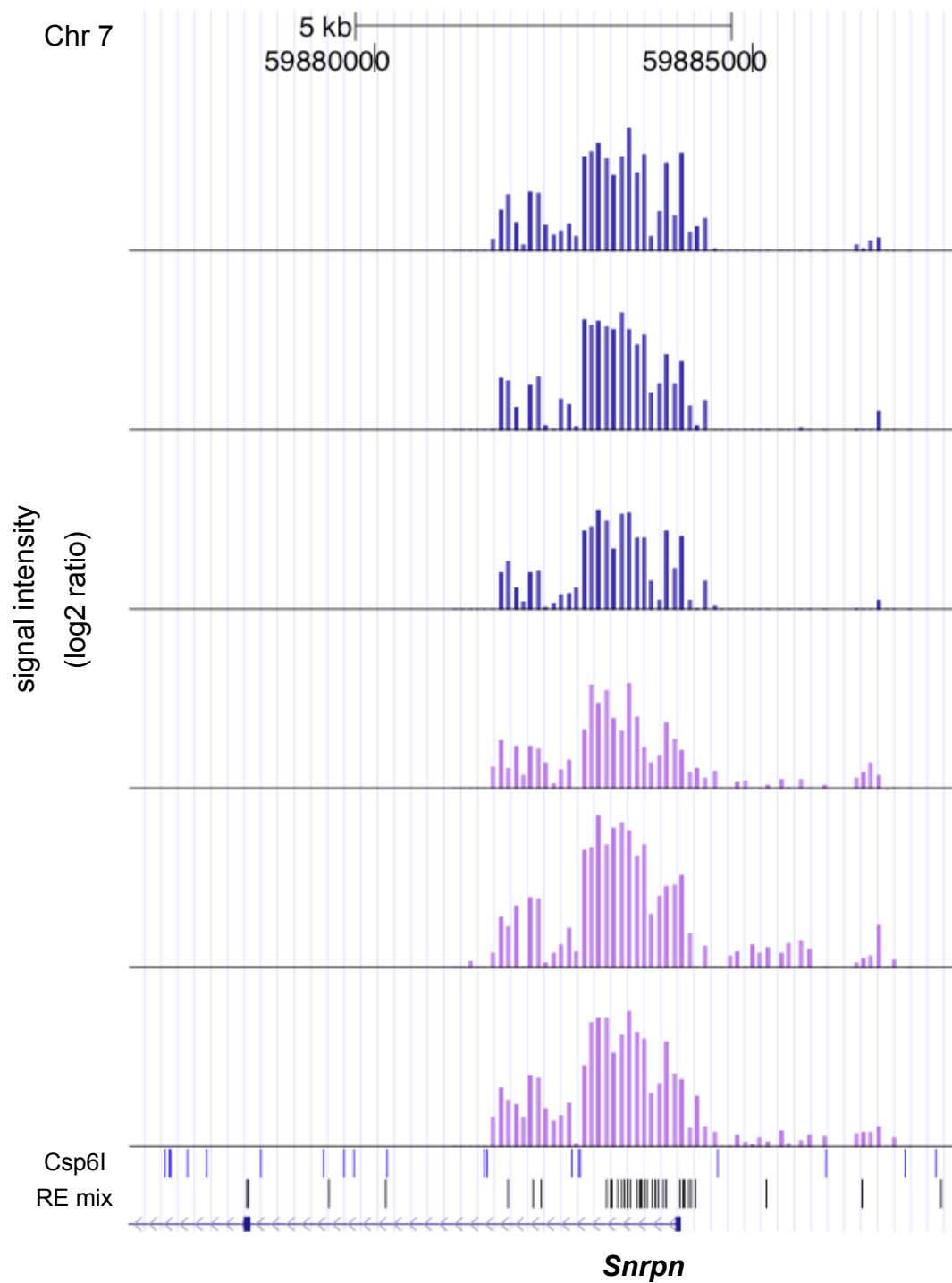

Supplement: Figure S1 — MAUD assay of differentially methylated controls. Results for Gtl2, H19, Mcst2, Mest, Nap1l5, Nnat, Peg3 and Snrpn are shown in alphabetical order. X-axis, nucleotide position along the mouse chromosome indicated. Y-axis, log2 ratio for methylated DNA vs. control (blue bars) and unmethylated DNA vs. control (purple bars). Ymax for each track, 7.2. For each gene, results are shown for the three mice assayed. Below the six tracks, the blue vertical lines show the location of Csp6I sites, and the black lines show the location of DNA methylation-sensitive HpaII AciI and HpyCH4IV sites. Below these lines, the transcription start site and structure of each gene is shown schematically: Positions of exons (bars) and introns (small arrows) are shown, with the direction of the arrows indicating the orientation of transcription. The figures were obtained by alignment of our custom tracks with annotation showing the location of the genes and restriction enzyme sites indicated [49], [50]. (0.29 MB PDF) [file pone.0013843.s001.pdf]

■ methylated DNA  
■ unmethylated DNA

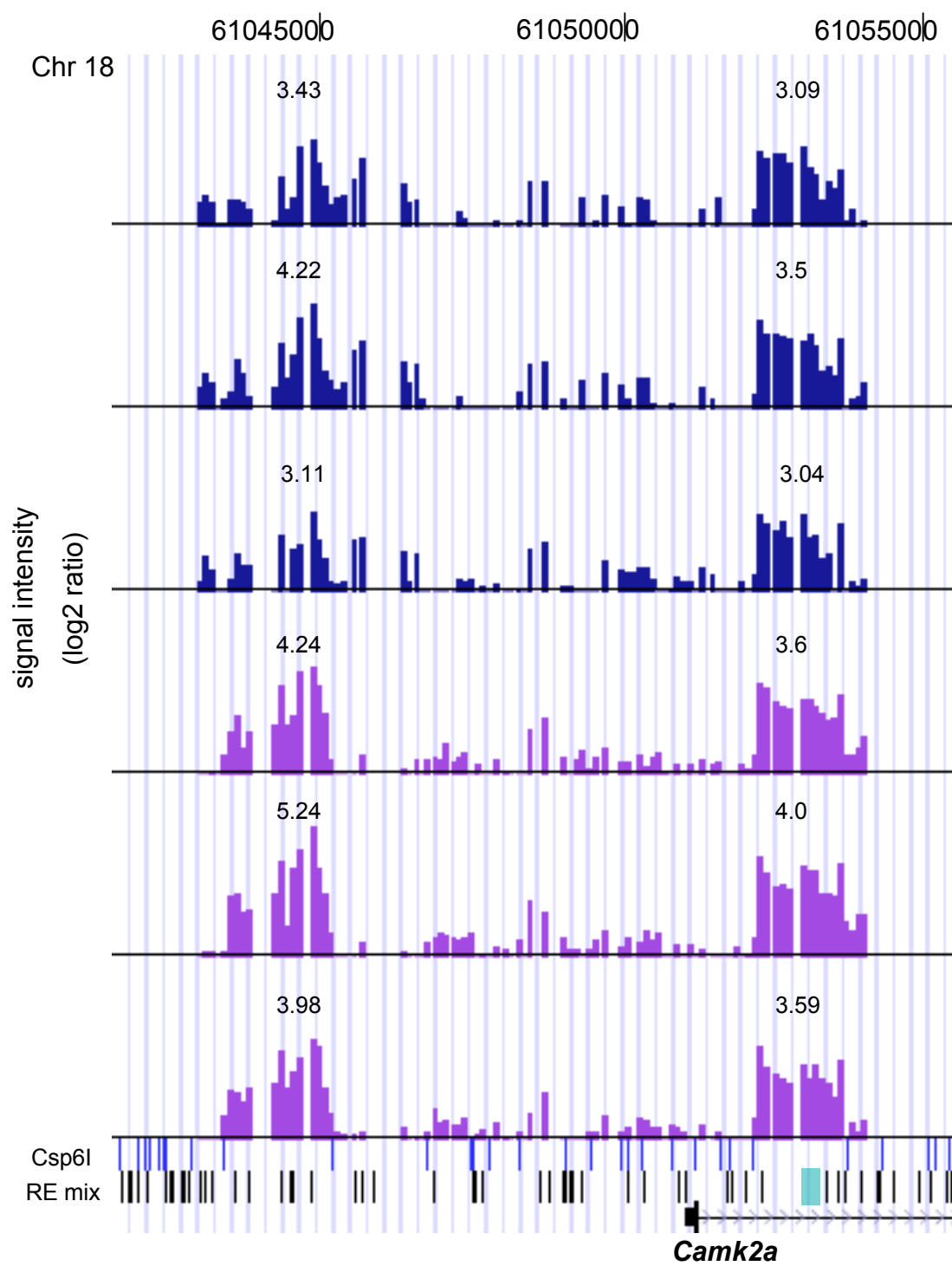

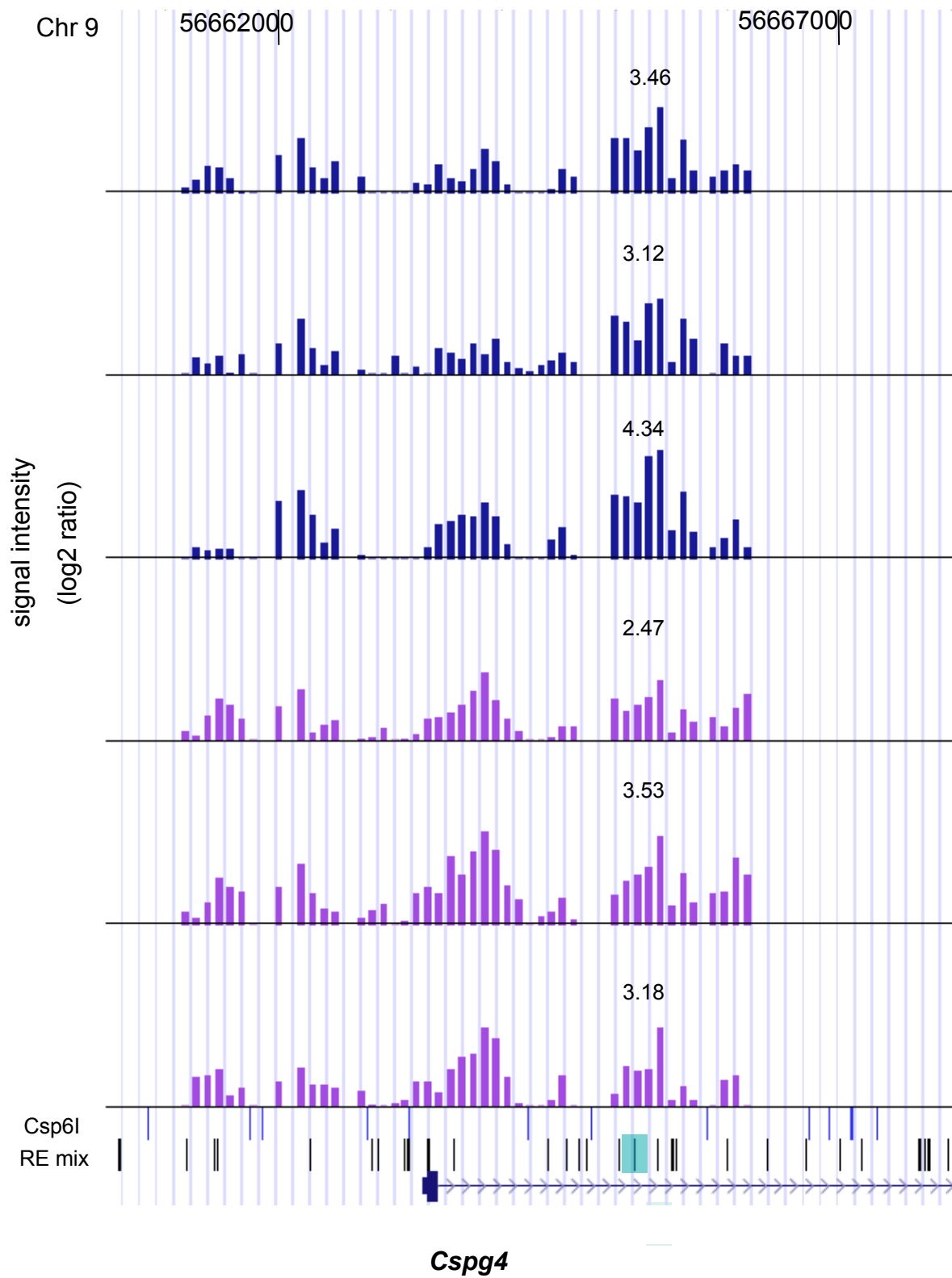

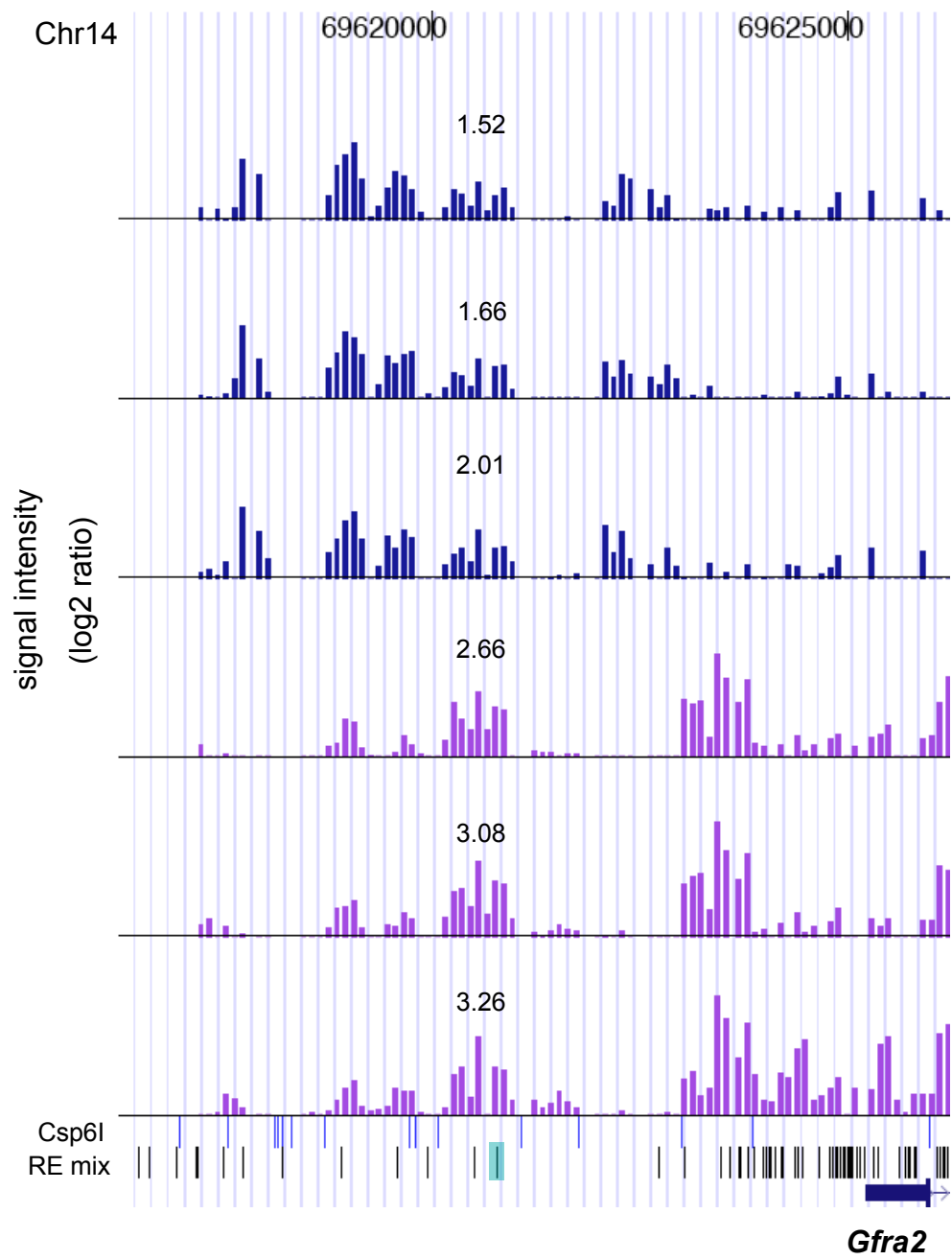

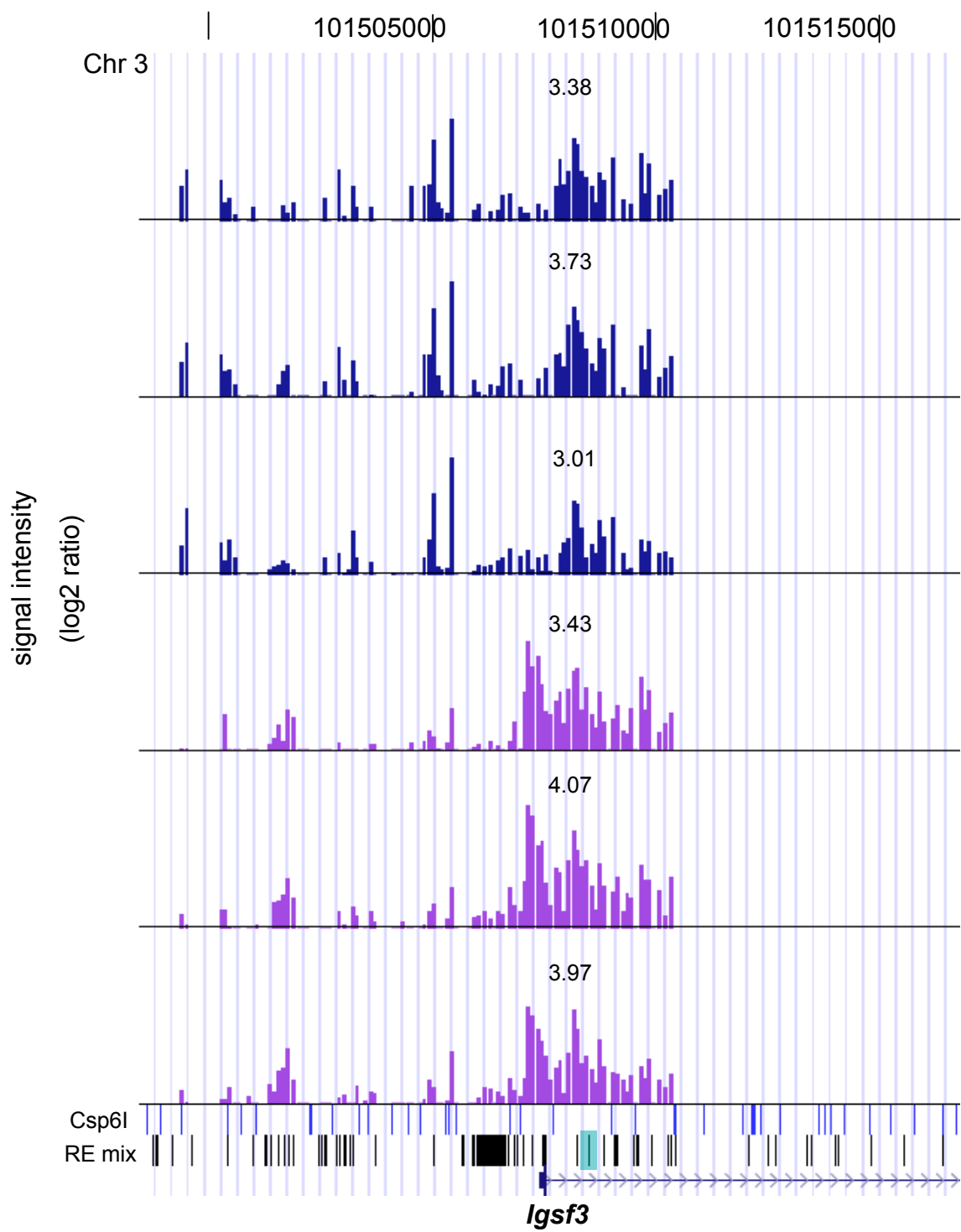

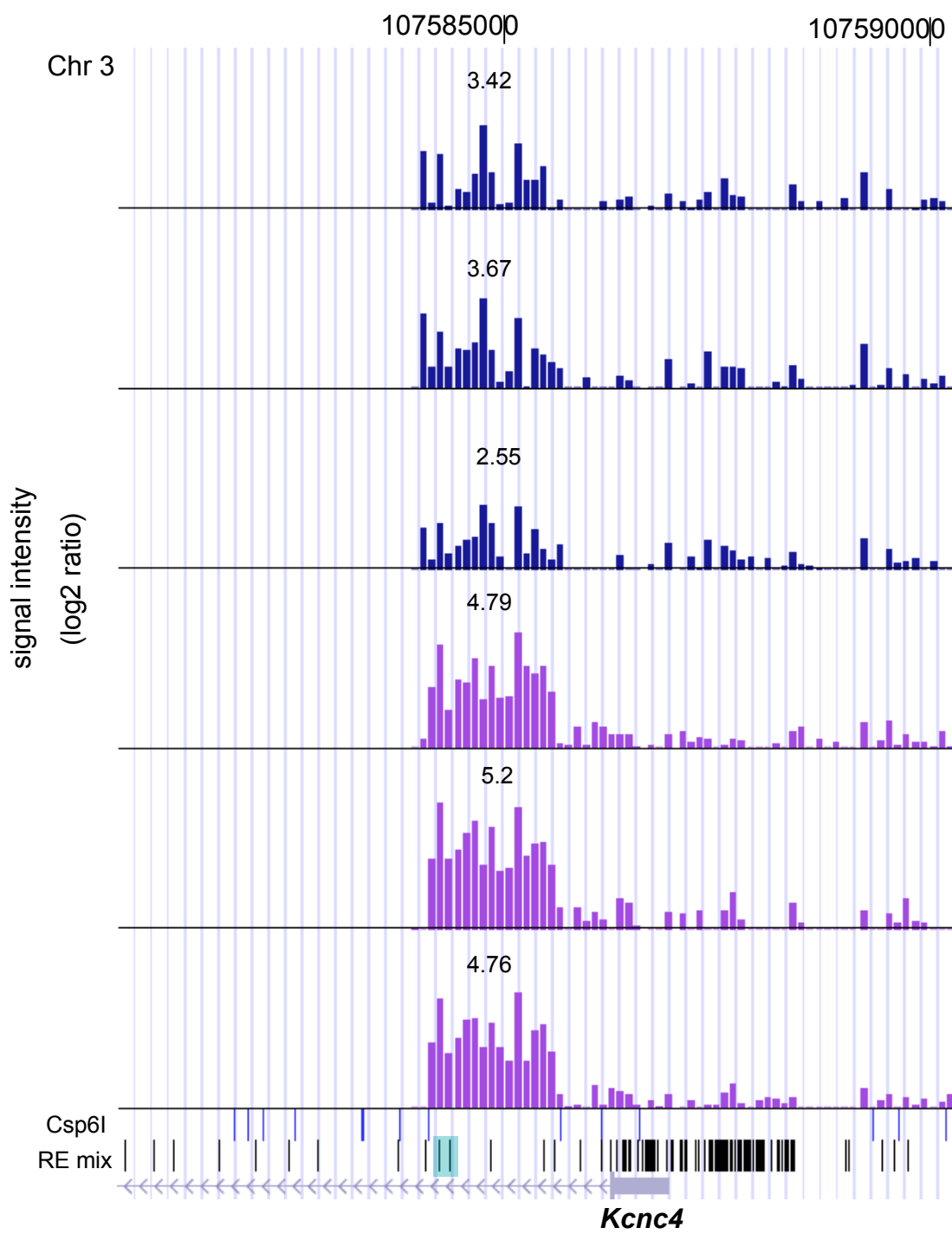

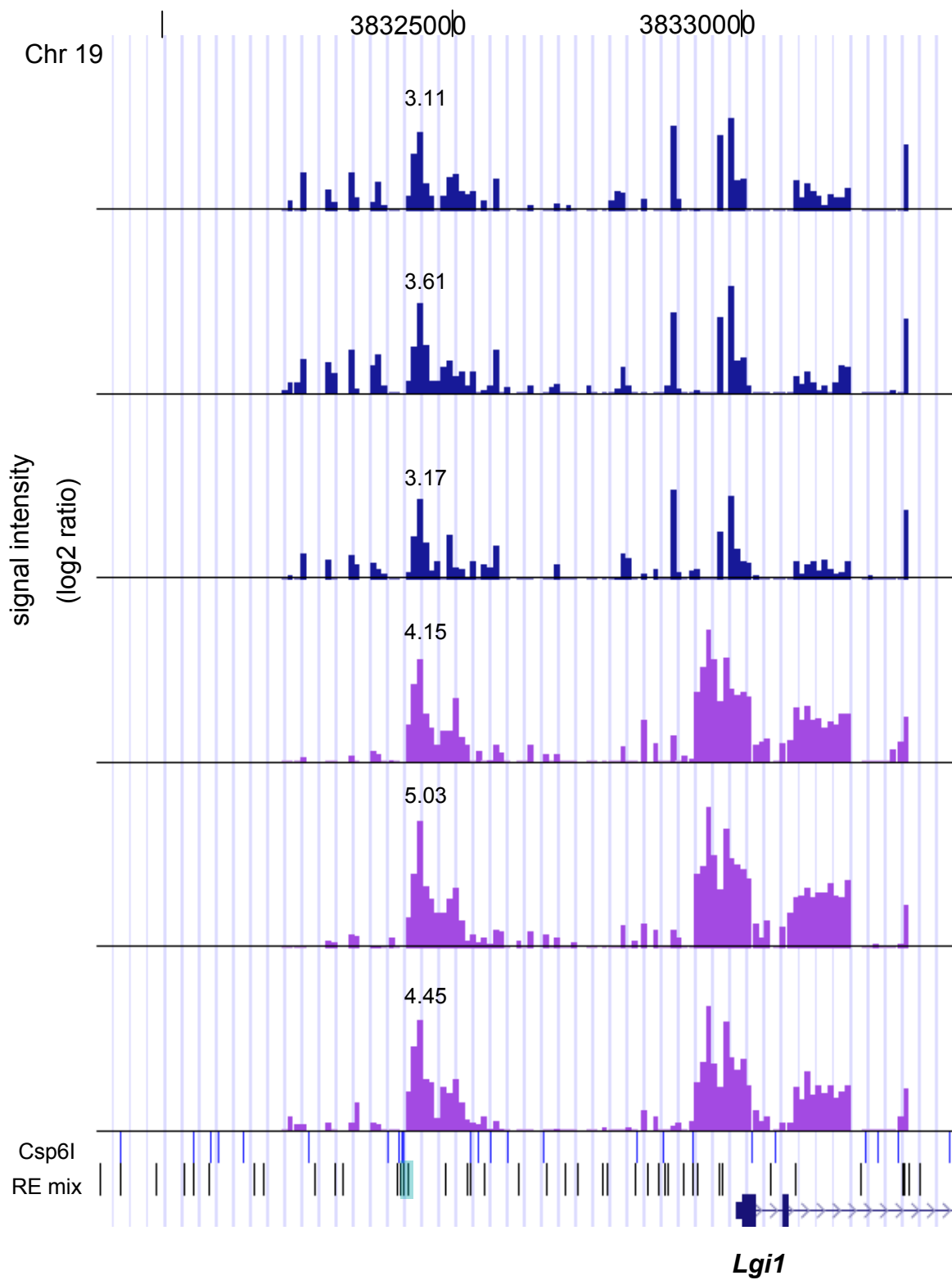

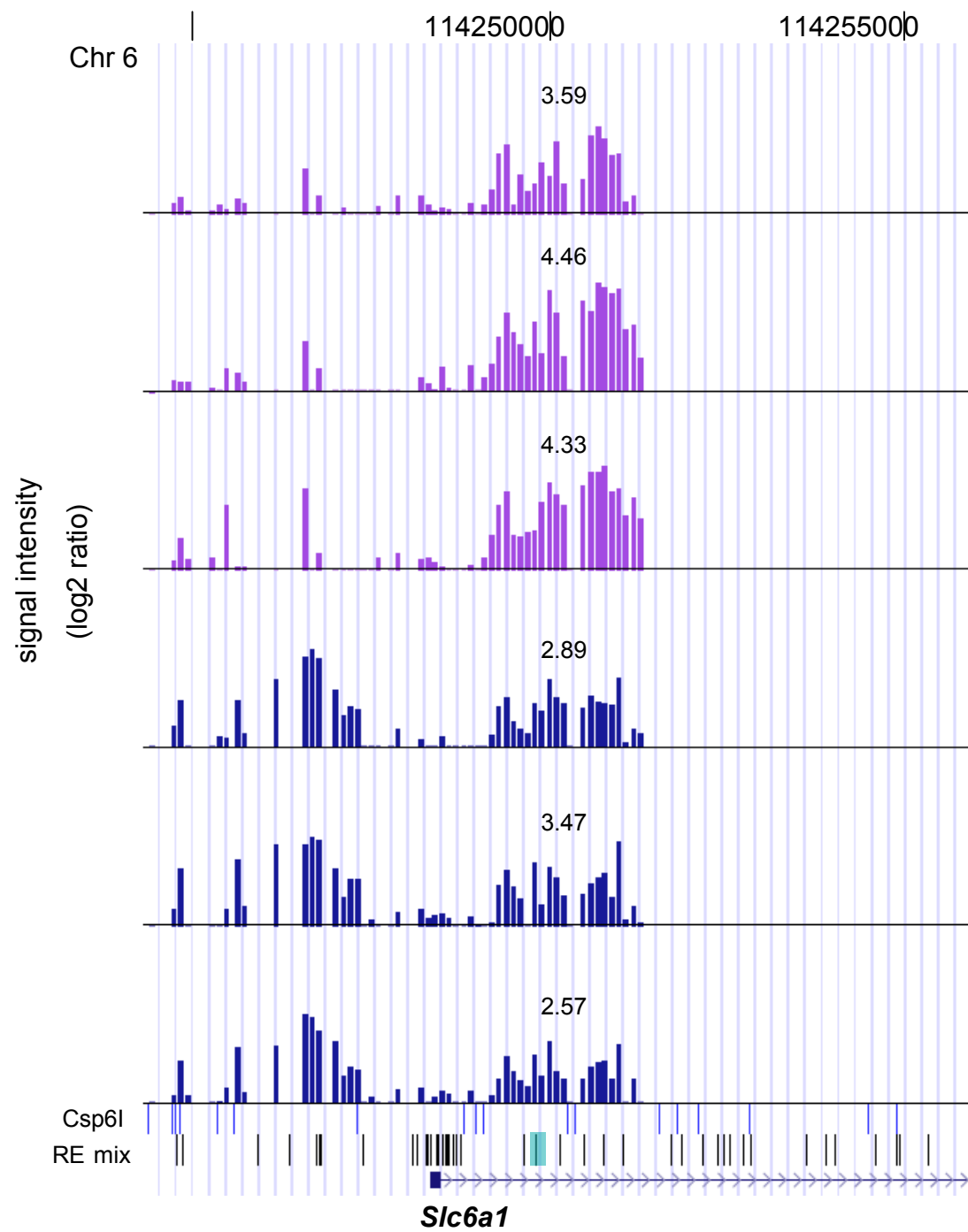

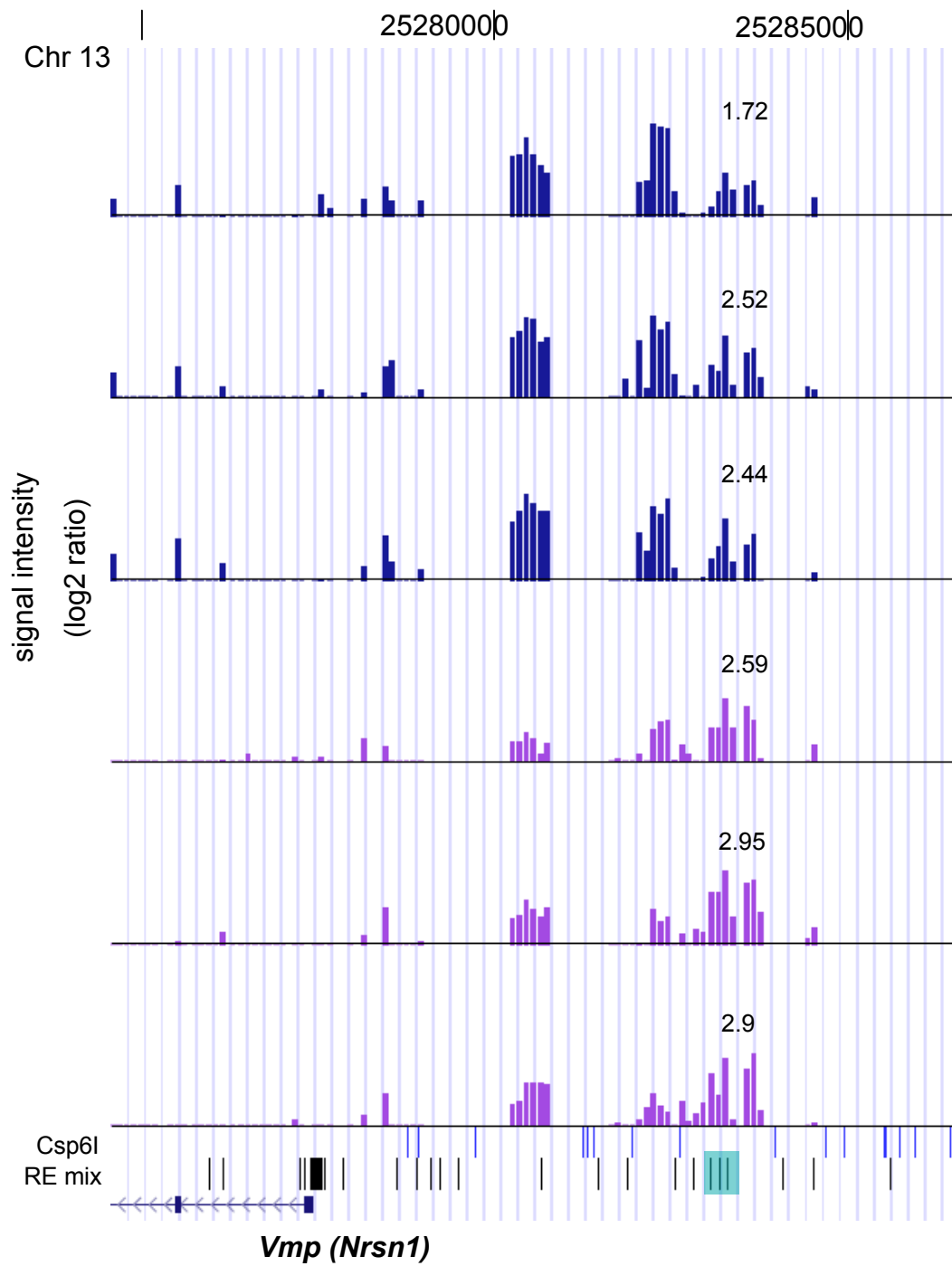

Supplement: Figure S2 — MAUD assay of genes showing monoallelic expression. Maximum peak height (log2) ratios are shown for peaks that are coincident in both tracks. The turquoise boxes highlight the DNA sequences analyzed directly for DNA methylation (Figure S3). (0.14 MB PDF) [file pone.0013843.s002.pdf]

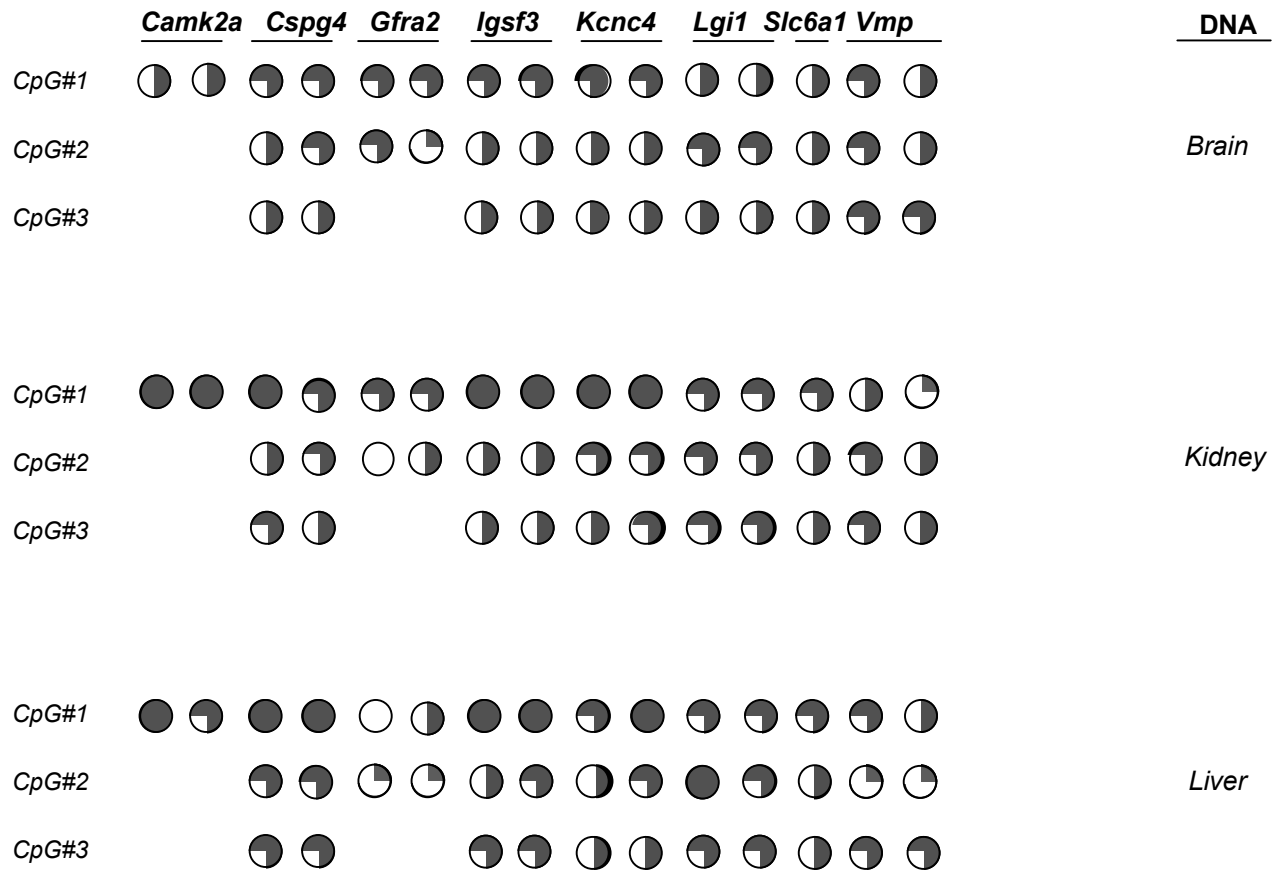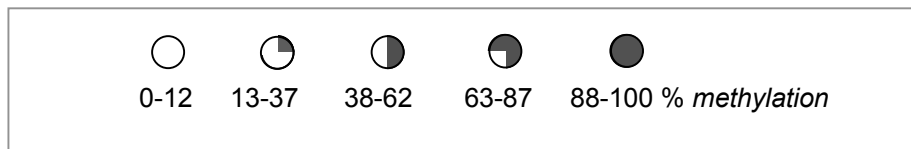

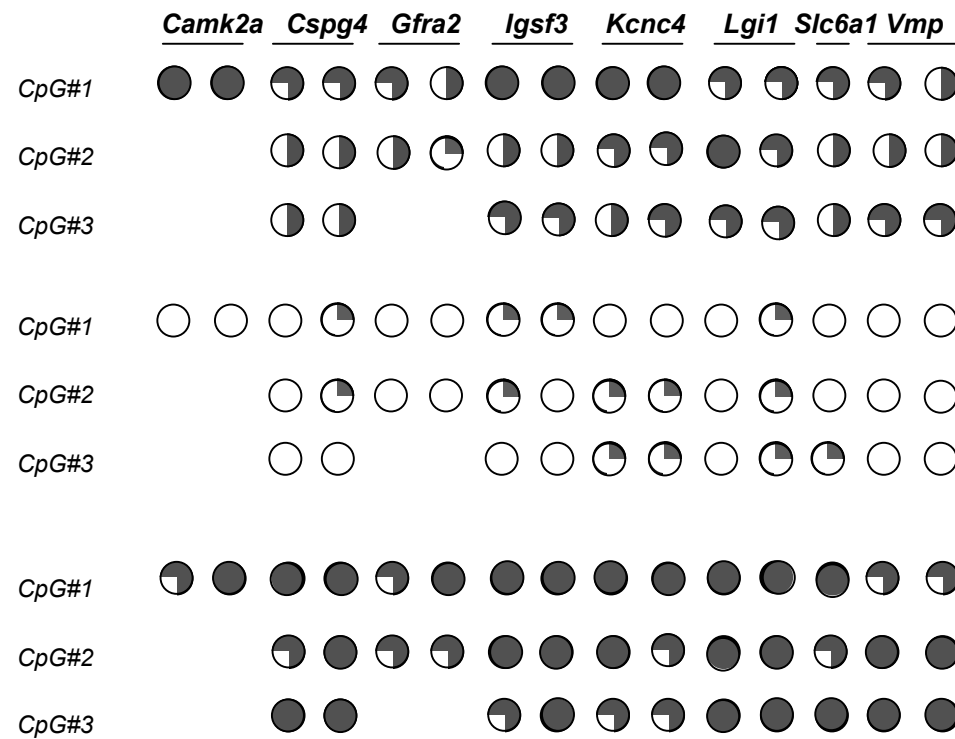

DNA

*Lung*

*Unmethylated amplicon*

*Methylated (SssI-treated) amplicon*

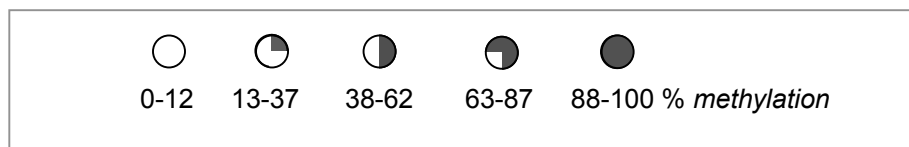

Supplement: Figure S3 — Partial DNA methylation of selected MAUD hits. DNA methylation analysis was carried out by use of the EpiTYPER system (Sequenom). Briefly, 1 µg bisulfite treated DNA from B6 brain, kidney, liver or lung was amplified with gene-specific primers using downstream primers that contain a T7 promoter tag. Following in vitro RNA synthesis and base-specific cleavage, MALDI-TOF mass spectrometry was used to determine relative DNA methylation based on the RNA cleavage pattern. Adjacent circles show biological replicates as indicated. Control panels show B6 (brain) DNA spiked prior to bisulfite treatment with either 1 ng of unmethylated amplicons or 1 ng of SssI-treated methylated amplicons, as shown. SssI treatment was carried out following the instructions of the manufacturer (New England Biolabs). Primers were designed with the aid of Primer3 or MethPrimer (www.urogene.org/methprimer), as appropriate. A list of relevant primers is available upon request. For each gene analyzed, CpG sites (or clusters) underlined below correspond to CpG #1, 2 or 3, respectively. For CpGs included within restriction enzyme sites AciI, HpaII or HpyCH4IV, the entire restriction site is underlined. Camk2a: GCAAGACTGCGTCACAGAGCG Cspg4: GGGGCCAGCCGTCGTCCTTGAGTCAAGCCTTGAAGGGTGGGAAGGGAGTCTGACTCCTGTCTGCGGTCCTCAGCCTGGACAAGAGCAGGAGGTGGGTGTAACGGGGTGTTGAA Gfra2: CCTAGCCTCACGCTCCAAGGATGAAGCCAGACAAGTCCAAAGTATAAATAACAAAAAAGGATTTTCATTCTCATGATTCTTTTTTTCCAGACAGGGCAGAGAGAAAAGGATTATCTCAGATGTCCTTAATGCAGGCACAGAATCTACAGACCCAGAGCTGCTGTCATTTTGTTTATTCATATGCTAACCCGGATTGACTAATG Igsf3: GCAGCTGGTCGCTCGCGTCTCCATTCTAGGTTTCTTGCACTTACAGGATTTATCCGTGGAGGTTGTCTCTGAATTATCTGCACCCTTATAAAAGTTAACAGGCATCCGGAATGAGGATG Kcnc4: GCAAAACCCCGGAATTAGGATGCTTGGTGAAGAGCTGGGTTCCCCCCACCACTTTTTTATGAATTGCTTATTCCCACTTGTGTGTCCAGGAACAGCTCAGAATTGGCCTCTGCCTATGTTCCTCCGCTGTGGGCAAGTCTTTTGGCTCCTGTGCCAGCCAGAGCGCCACAGCATGA Lgi1: CCTACAGGCACGTTGGATACCCCCACCTTGTACACAGTGAATGCTGGCCCTGGTTTGCAGCAGTTTCCACTTCCATGAAGCTTTTAATCCTCTCGAATCAACATTATCACCACCACCATCATCATCCTCATCC [file pone.0013843.s003.pdf]

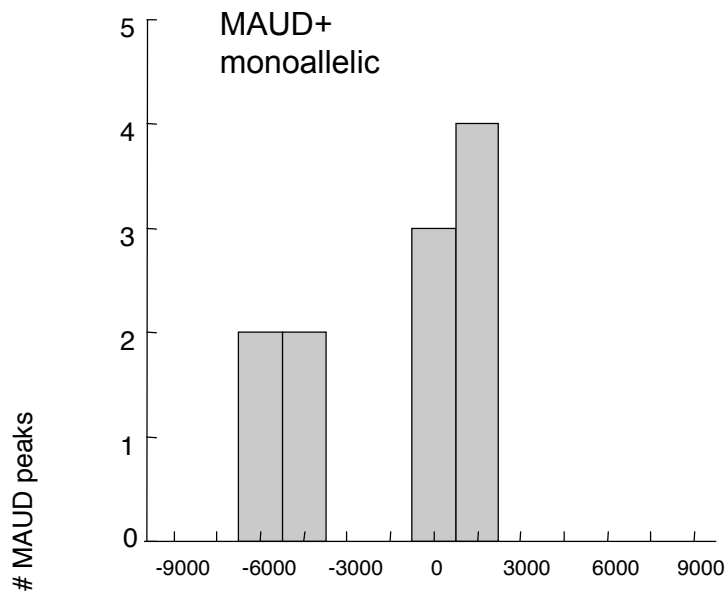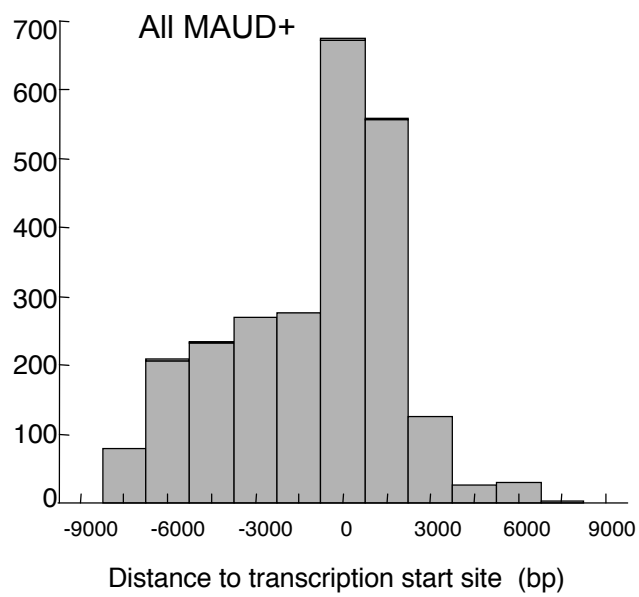

Supplement: Figure S5 — Distance of MAUD peaks from transcription start sites. Top, MAUD hits with monoallelic expression; bottom, all MAUD hits. (0.02 MB PDF) [file pone.0013843.s005.pdf]
