# Supplementary material for: Dual DNA Methylation Patterns in the CNS Reveal Developmentally Poised Chromatin and Monoallelic Expression of Critical Genes
Source: PLoS One. 2010 Nov 4;5(11):e13843. doi: 10.1371/journal.pone.0013843 (PMC2973945; doi:10.1371/journal.pone.0013843)
Supplement: Figure S4 — RT-PCR results for genes that show allele-specific expression in NSC lines. (A-I). Results are shown for Camk2a, Cspg4, Gfra2, Igsf3, Kcnc4, Lgi1, Slc6a1, Unc5a and Vmp, in alphabetical order. Automated sequencing of RT-PCR products was performed after identification of B6/JF1 SNPs included within the amplified sequences. Representative results are shown for brain tissue from B6 and JF1 mice, F1 hybrid progeny, clonal NSC lines and neuronal and astrocytic populations derived from them, as indicated. PCR results for genomic DNA from relevant clonal lines are also shown, verifying the presence of both alleles. The relative intensity (peak height) of the signal for each base at SNP sites was measured to determine the % signal. For each sample, the percent expression of the predominant allele is shown. The number of replicates is in parentheses. For samples including at least three technical replicates, the SEM is given; otherwise the range is shown. CI, confidence interval. (J). Standard curves confirm the linearity of the assay. For each gene shown, RT-PCR products of strains B6 and JF1 were mixed in the proportions shown prior to automated sequencing (% input). Representative standard curves are shown for the mismatches C vs. C+T, T vs. C+T, A vs. A+G, and C vs. A+C, as indicated. (K). Comparison of Nes (nestin) RNA in neural stem cells vs. atrocytes/neurons (left graph) and of Dcx (doublecortin) RNA in astrocytes vs. neurons (right graph). Nestin and doublecortin are markers of neural progenitor cells in developing and adult brain, and of cells of neural lineage, respectively [51], [52]. Quantitative real-time PCR of cDNA was performed in triplicate. For each experiment, values were normalized to those obtained for the housekeeping gene Pgk1, with the average relative ratio set at 1.0. Results are shown for all cell lines except for the outliers 2A5 (left graph) and 3A1 (right graph). (Outliers showed values >2 standard deviations from the mean.) Error bars, ± SEM. [file pone.0013843.s004.pdf]

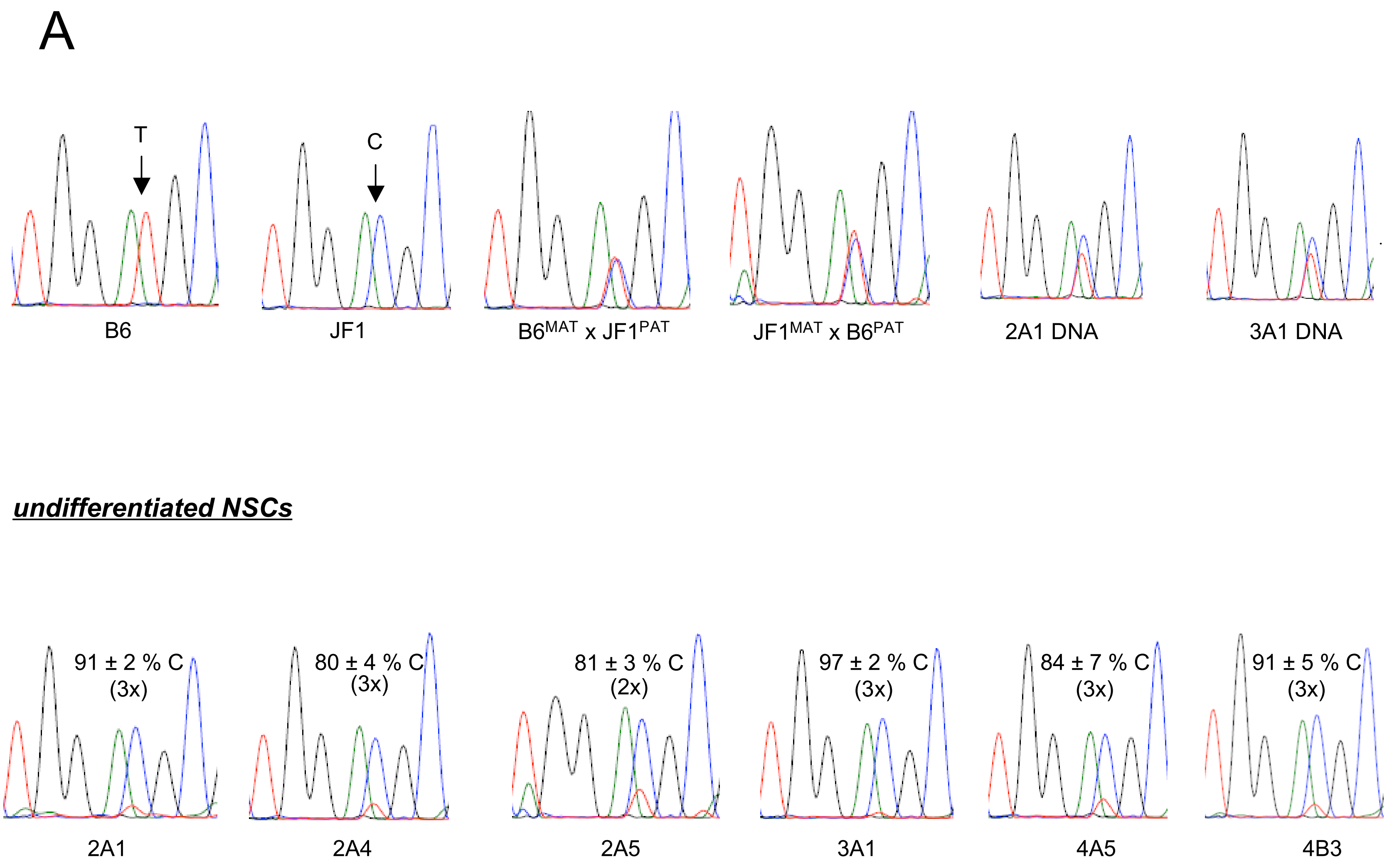

Camk2a (primer set 837/838; 837/1506 for DNA)

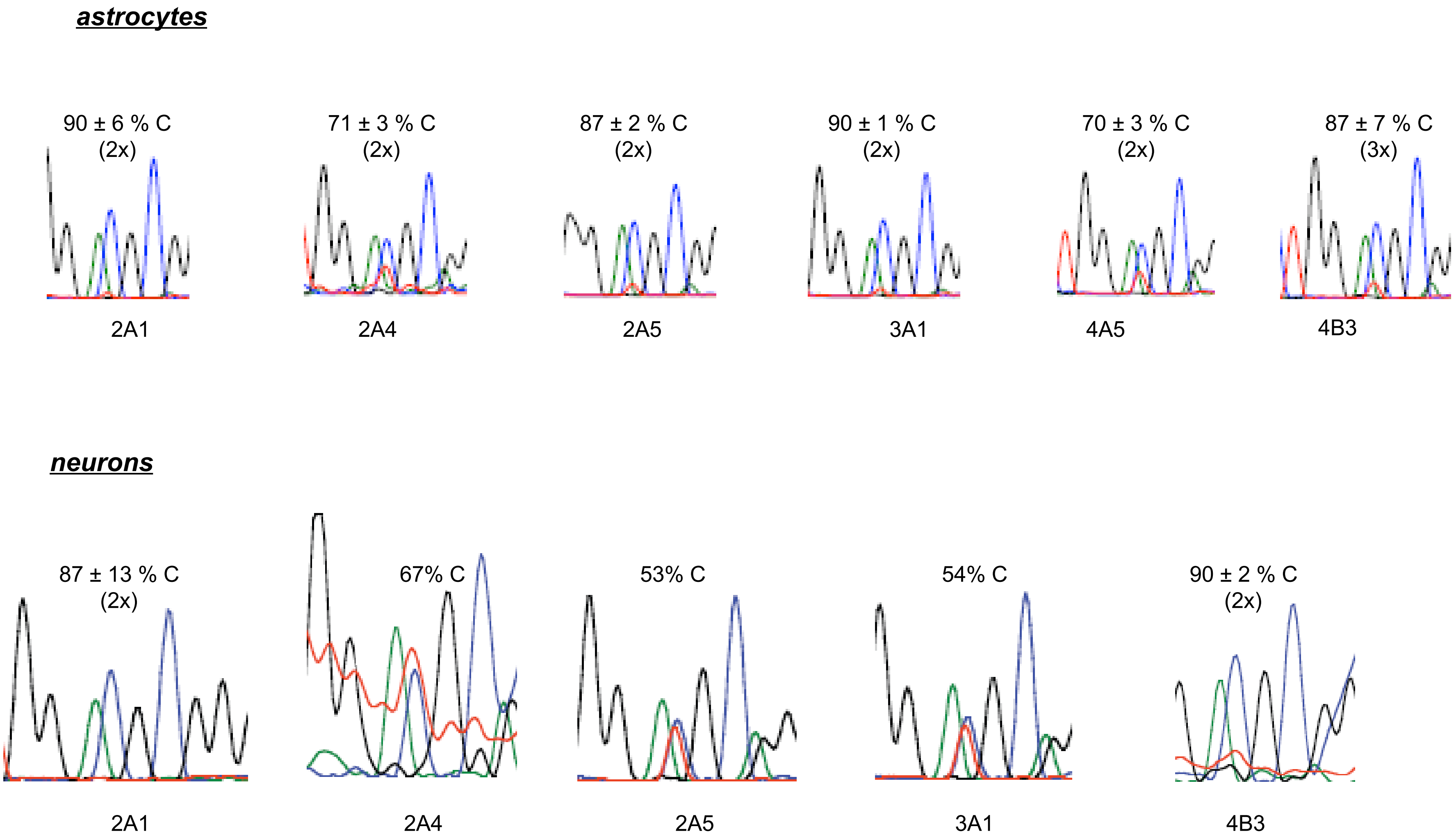

Camk2a primer set 837/838 (T B6: C JF1)

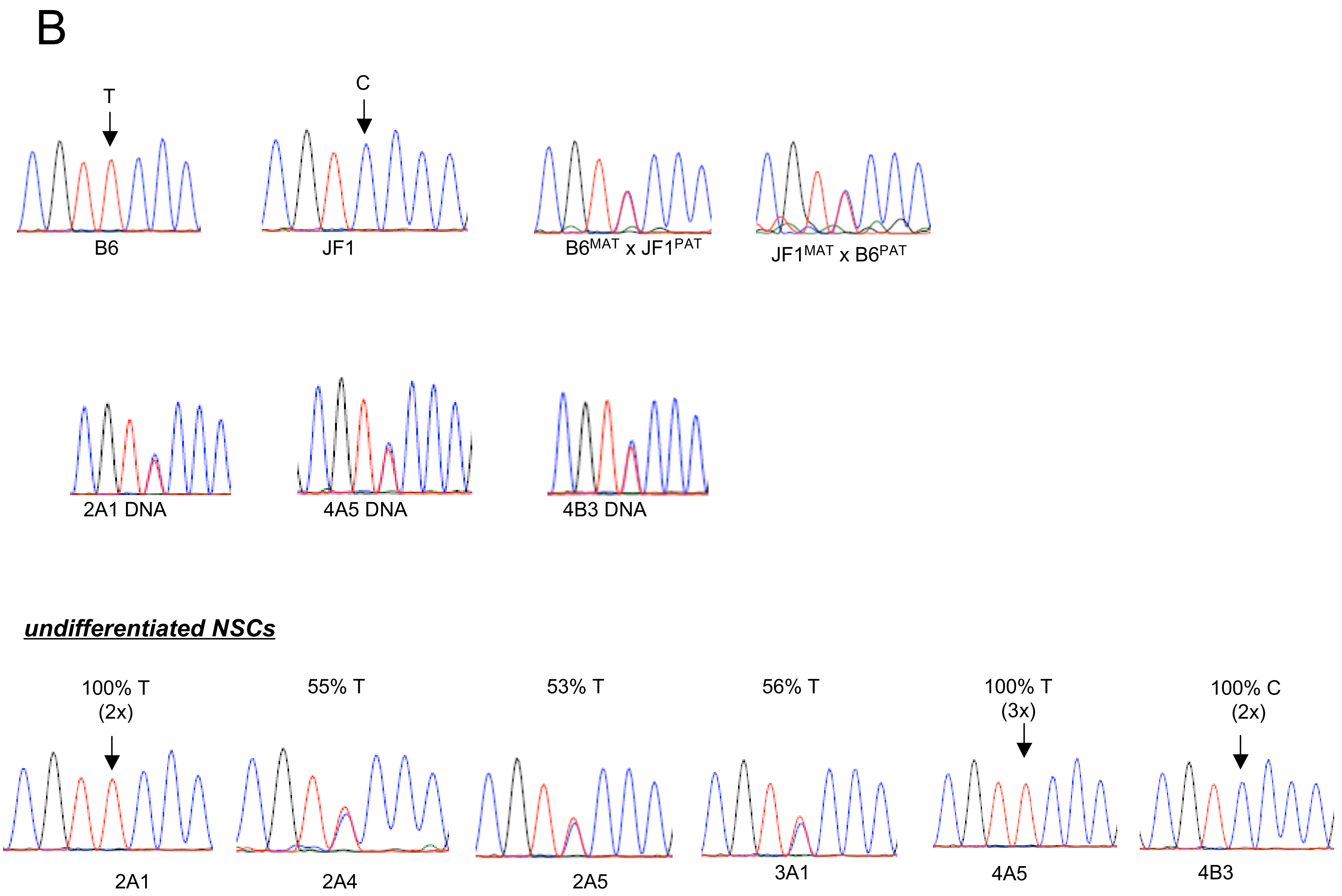

Cspg4 (primer set 917/918; 1503/1504 for DNA)

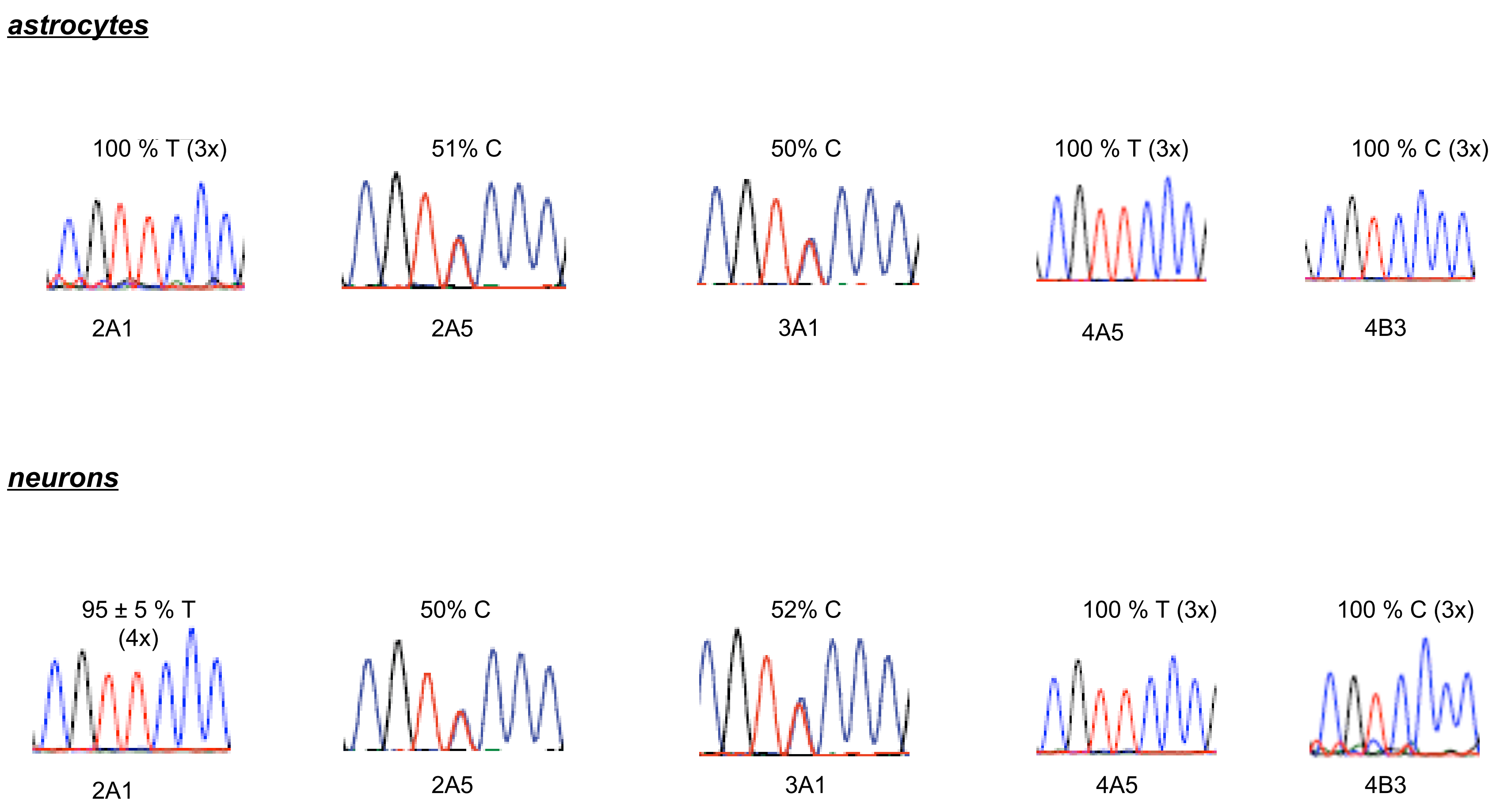

Cspg4 primer set 917/918 (T B6: C JF1)

C

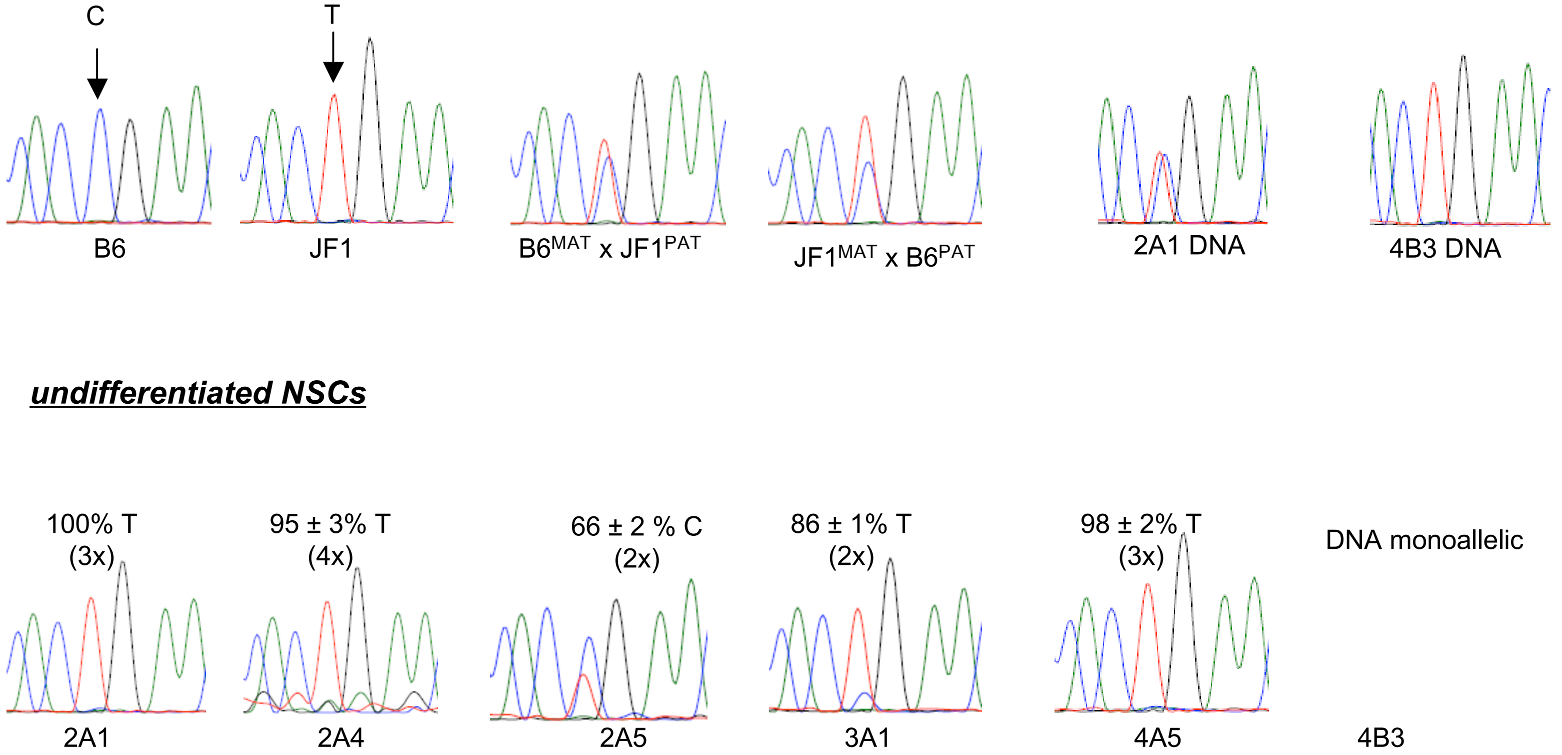

Gfra2 (primer set 853/854; 853/1510 for DNA)

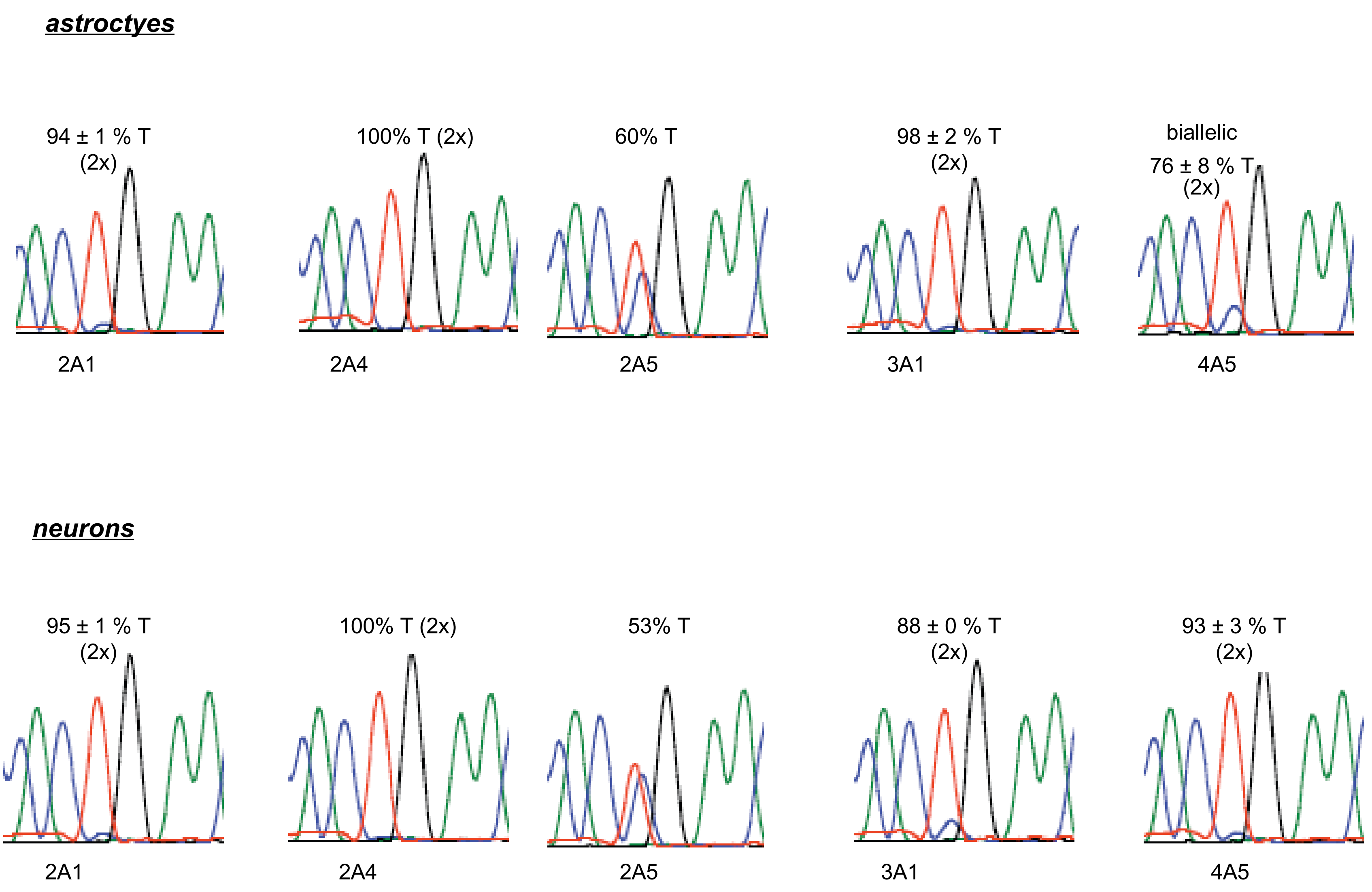

Gfra2 primer set 853/854 (C B6: T JF1)

D

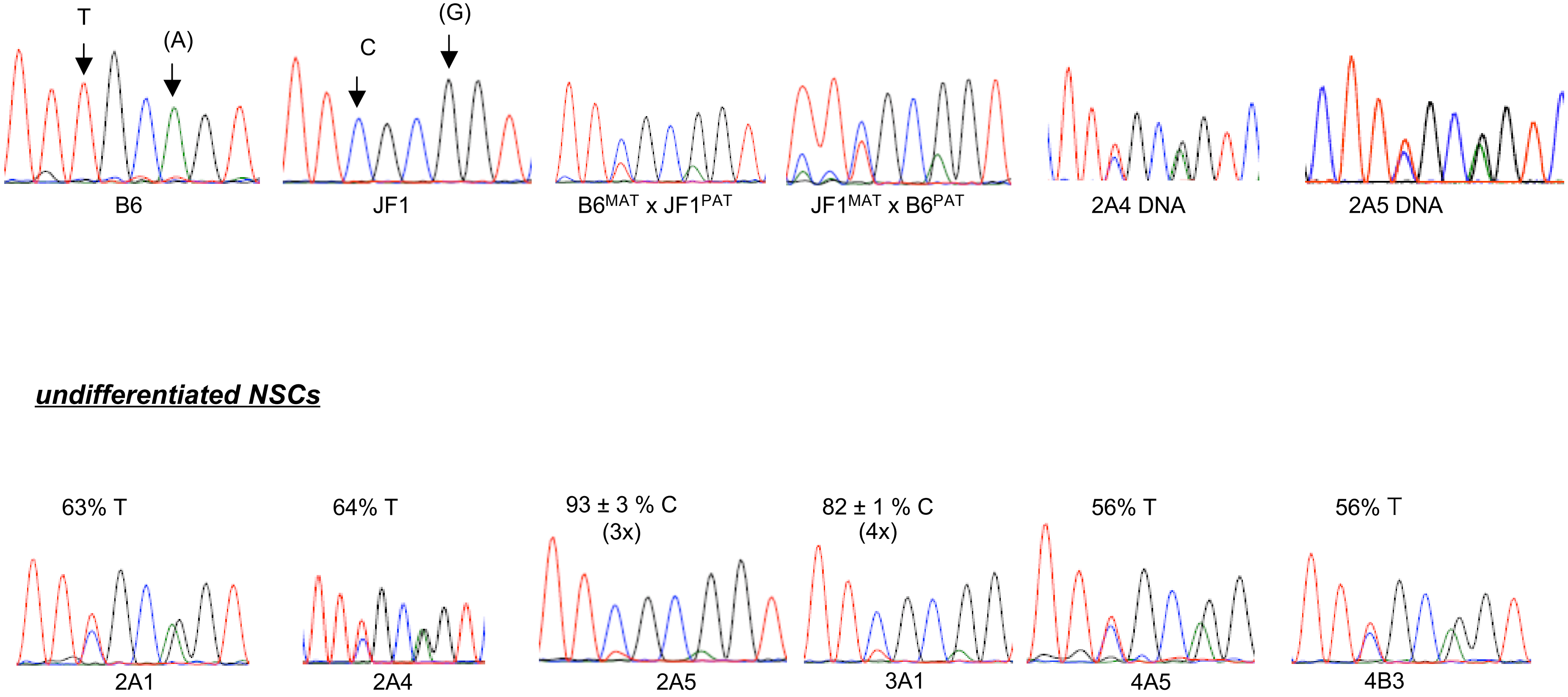

Igsf3 (primer set 801/802;1523/1524 for DNA)

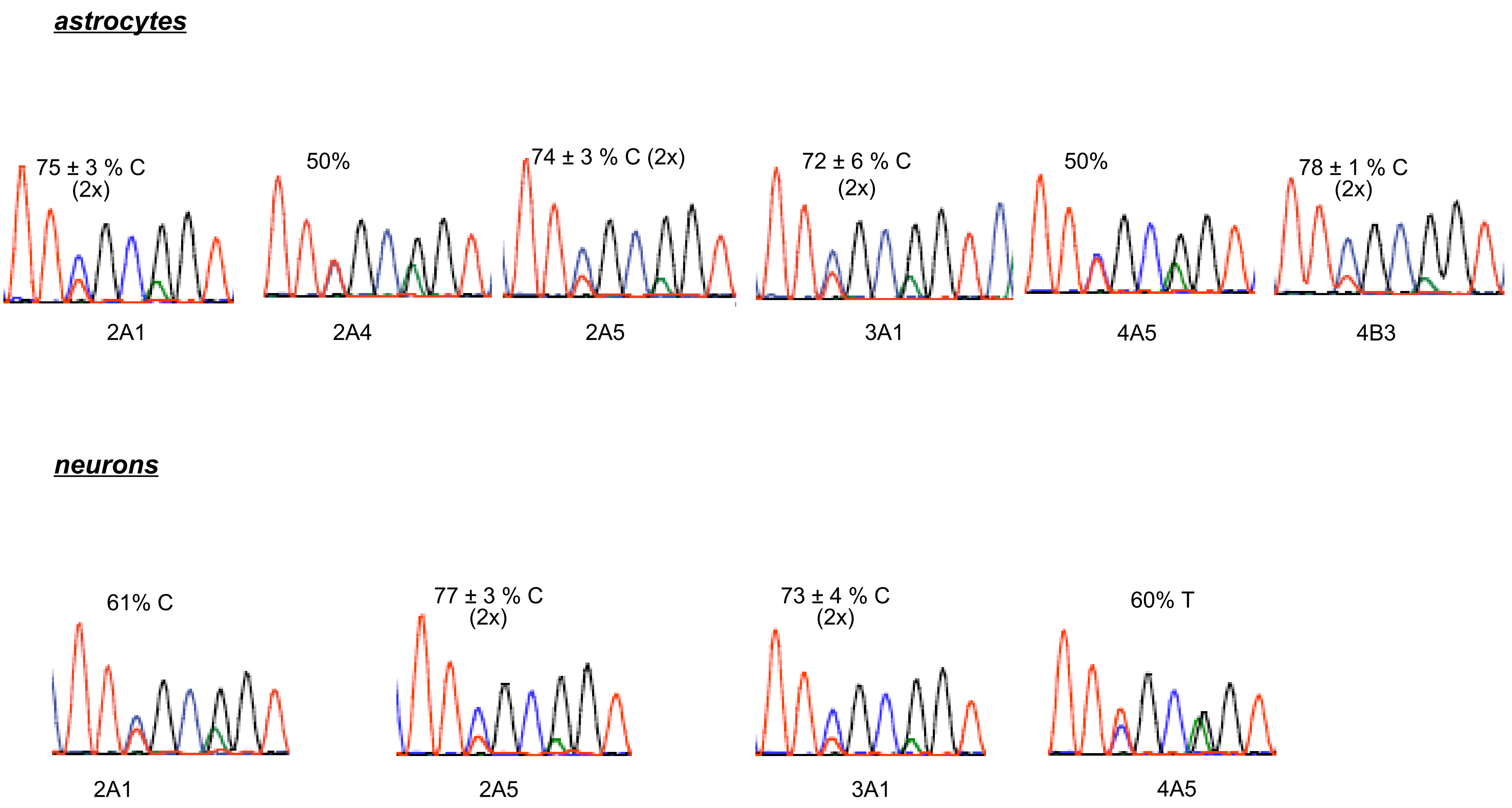

Igsf3 primer set 801/802 (T B6: C JF1, A B6: G JF1)

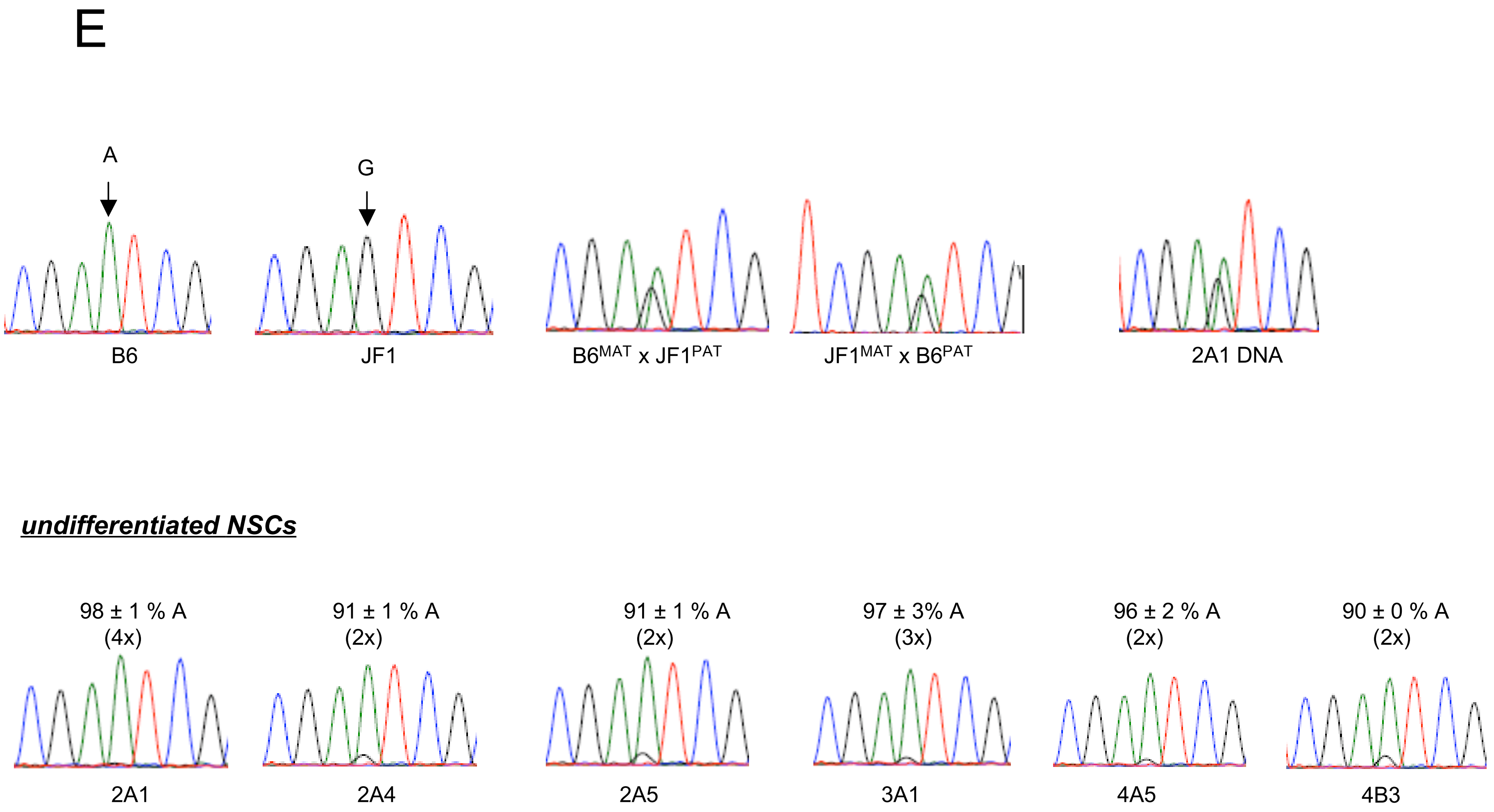

Kcnc4 (primer set 953/954; 953/1516 for DNA)

astrocytes

93 ± 3 % A
(2x)

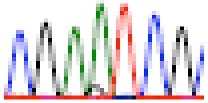

2A1

90 ± 0 % A
(2x)

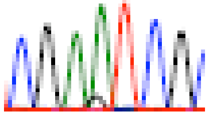

2A5

94 ± 4 % A
(2x)

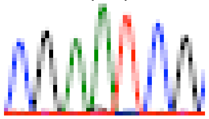

3A1

69 ± 2 % A
(2x)

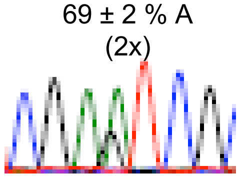

4A5

64 ± 3 % A
(2x)

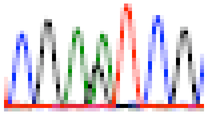

4B3

neurons

83 ± 10 % A
(2x)

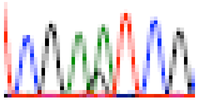

2A5

93 ± 0 % A
(2x)

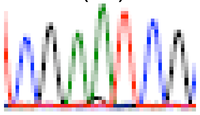

4B3

Kcnc4 primer set 953/954 (A B6: G JF1)

F

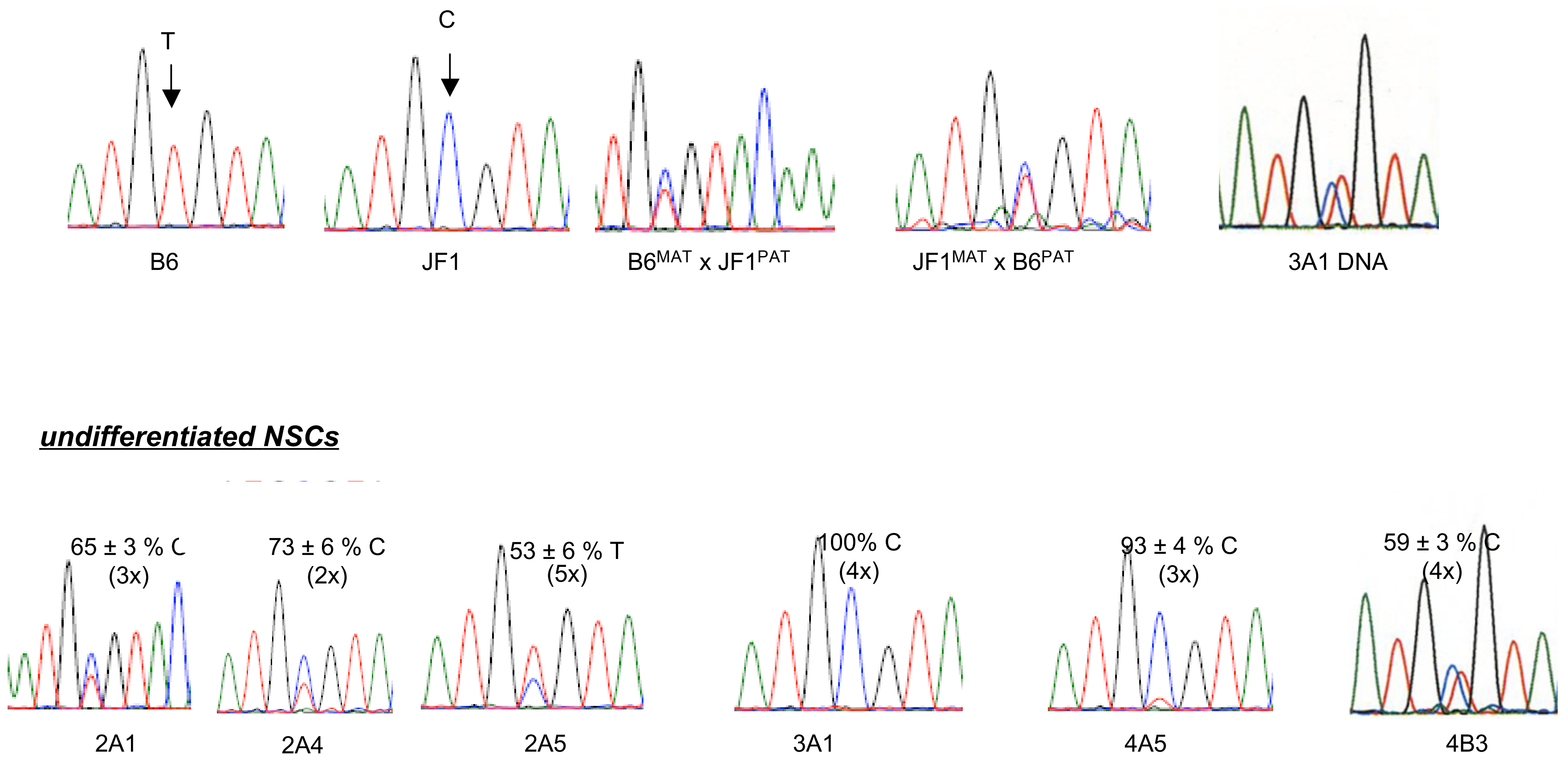

Lgi1 (primer set 871/872)

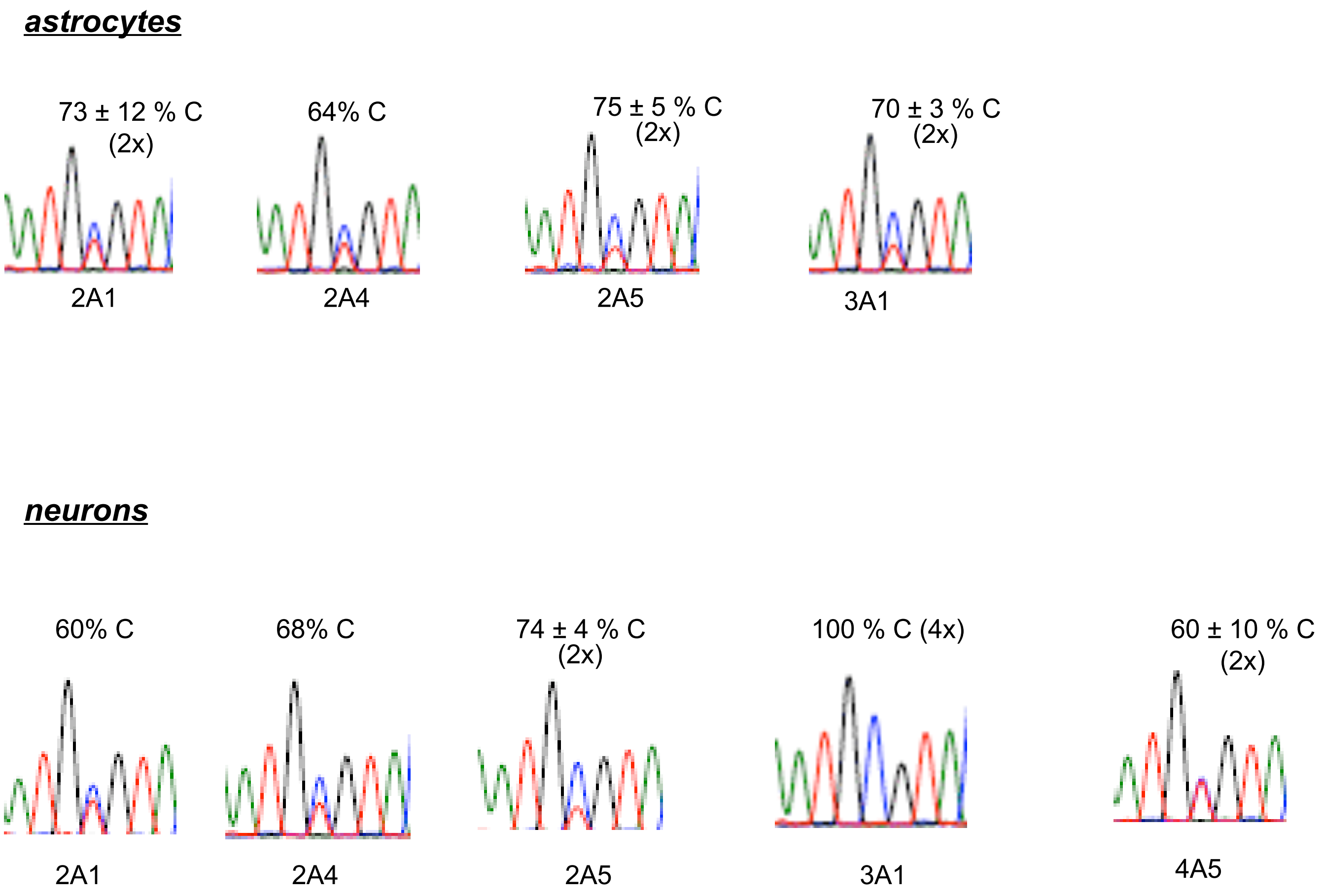

Lgi1 primer set 871/872 (T B6: C JF1)

G

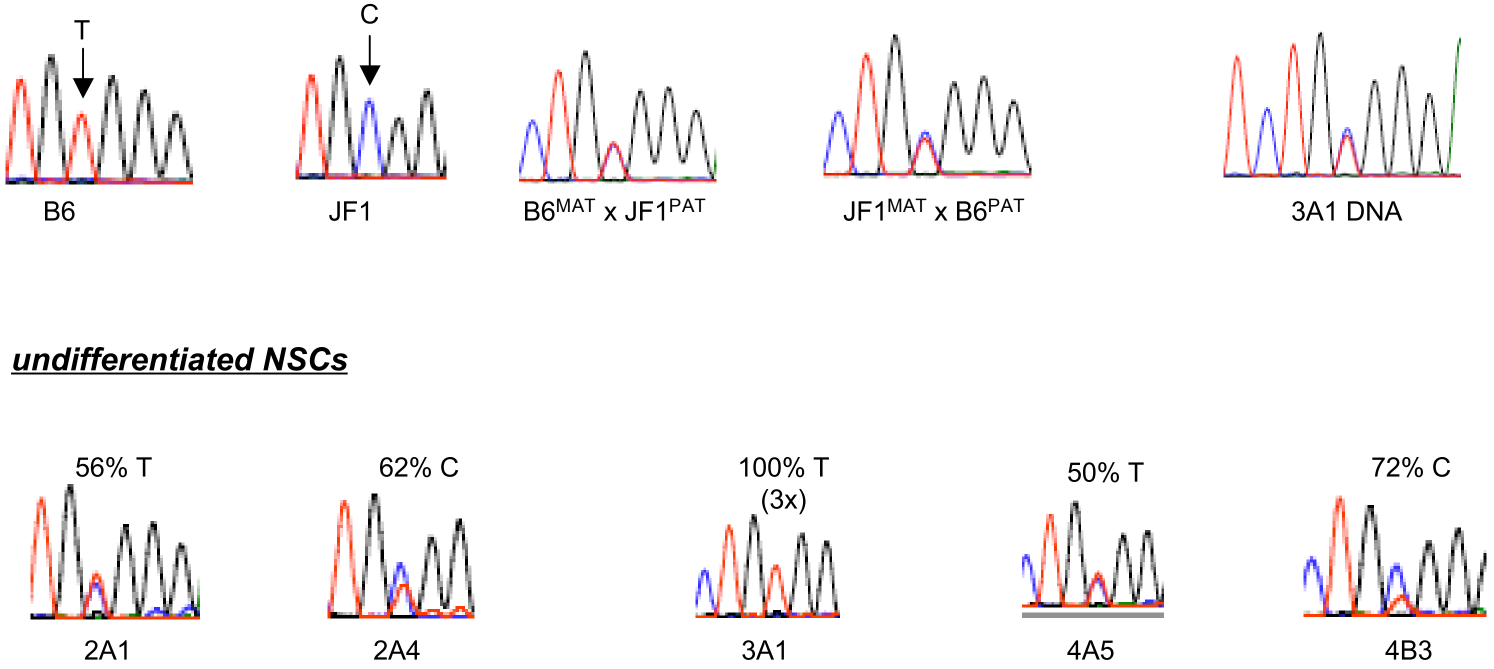

Slc6a1 (primer set 795/796; 1517/1518 for DNA)

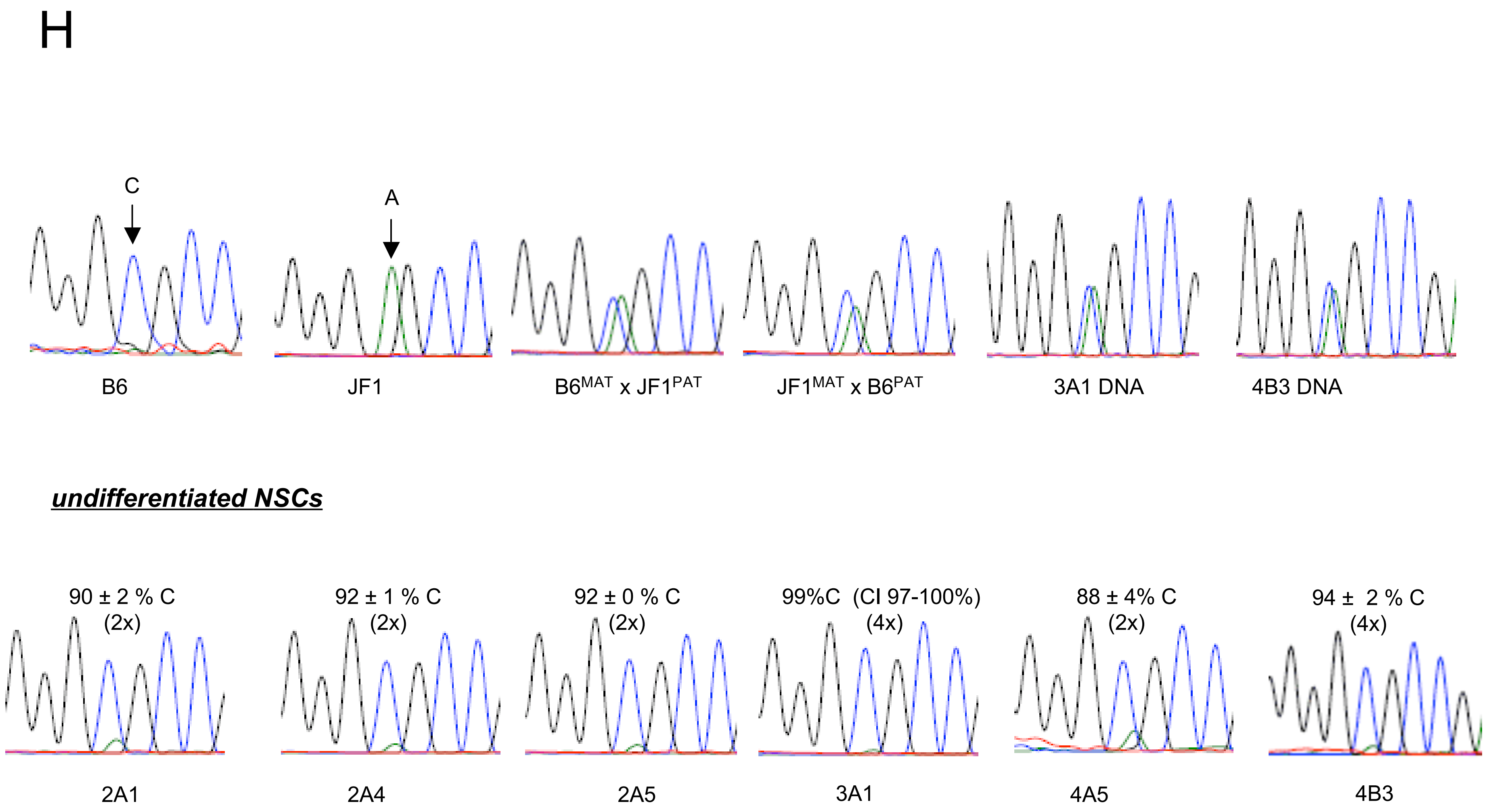

Unc5a (primer set 553/554; 673/674 for DNA)

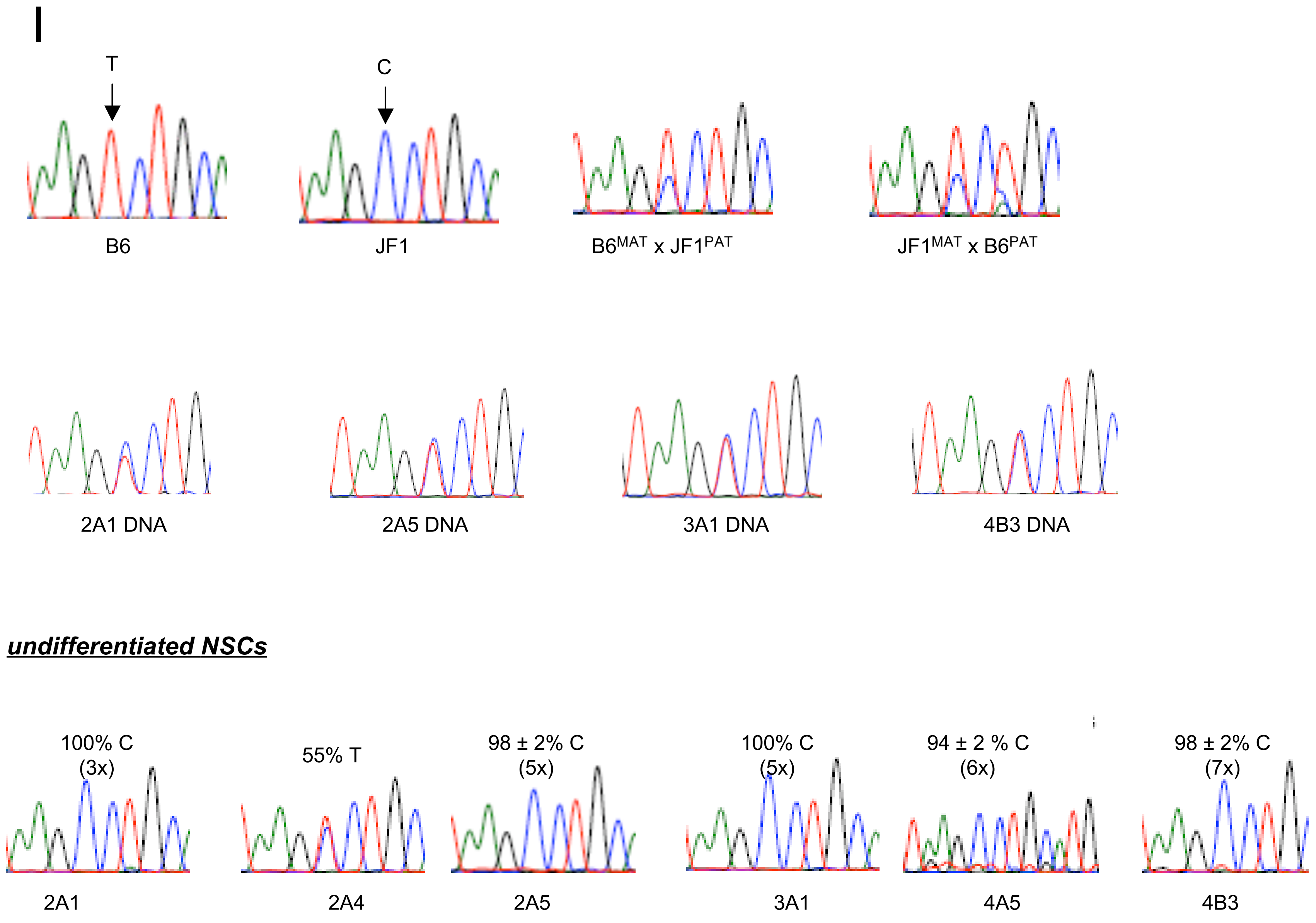

Vmp (primer set 555/556; 685/556 for DNA))

J

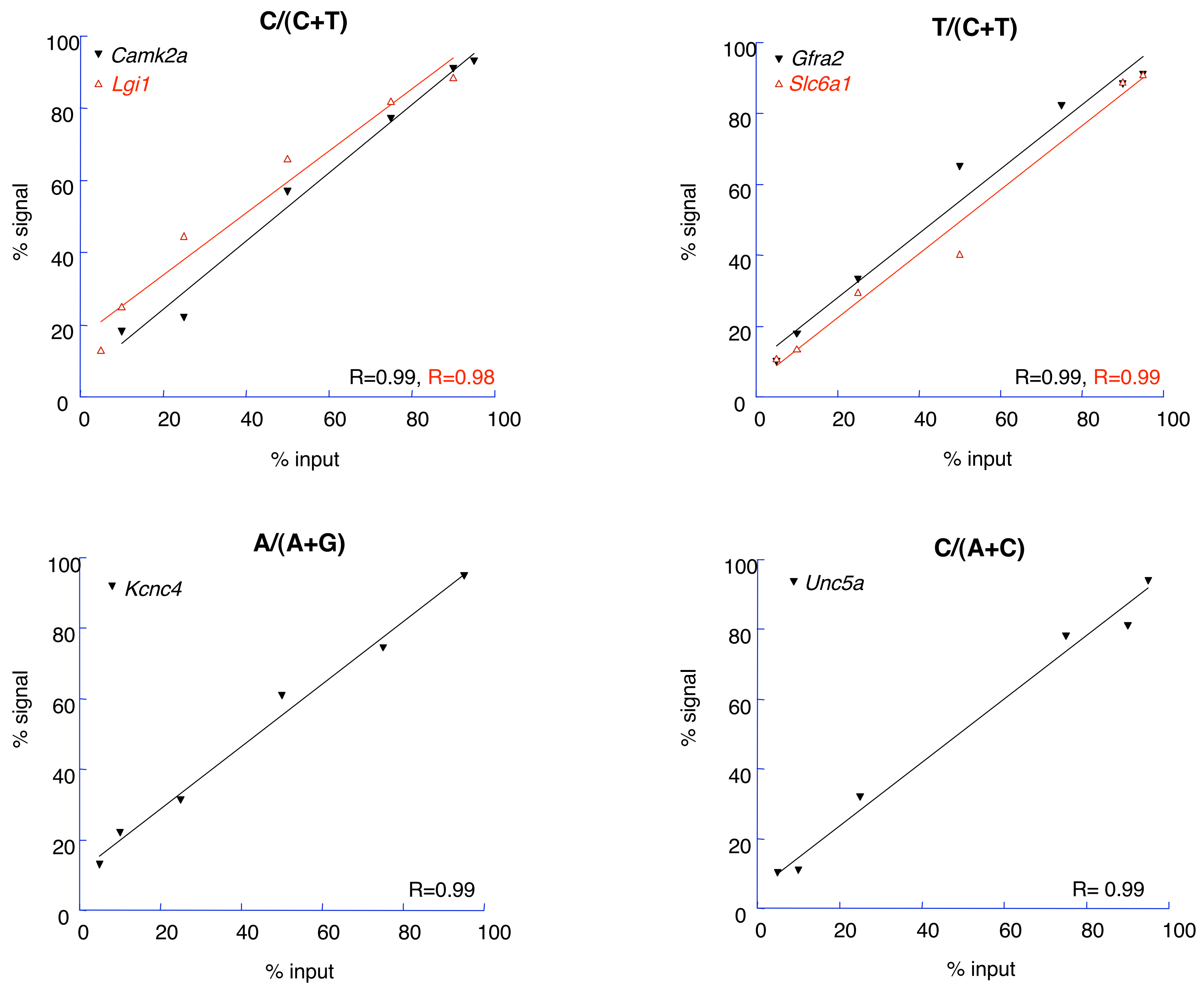

C/(C+T)
Camk2a
Lgi1
R=0.99, R=0.98
% signal
% input
T/(C+T)
Gfra2
Slc6a1
R=0.99, R=0.99
% signal
% input
A/(A+G)
Kcnc4
R=0.99
% signal
% input
C/(A+C)
Unc5a
R= 0.99
% signal
% input

K

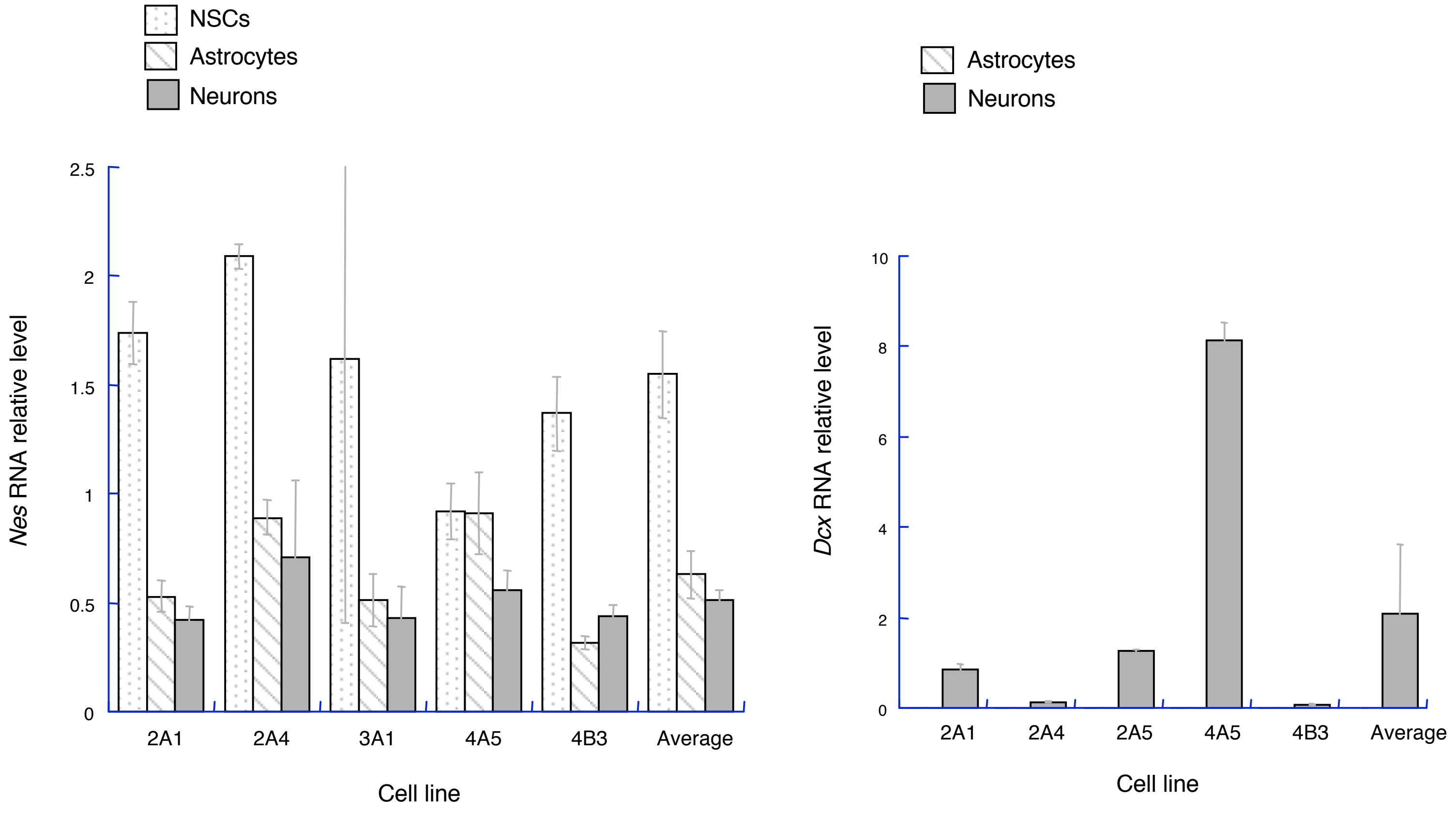
NSCs
Astrocytes
Neurons
2.5
2
1.5
1
0.5
0
Nes RNA relative level
2A1
2A4
3A1
4A5
4B3
Average
Cell line
Astrocytes
Neurons
10
8
6
4
2
0
Dcx RNA relative level
2A1
2A4
2A5
4A5
4B3
Average
Cell line
