# Supplementary material for: Dual DNA Methylation Patterns in the CNS Reveal Developmentally Poised Chromatin and Monoallelic Expression of Critical Genes
Source: PLoS One. 2010 Nov 4;5(11):e13843. doi: 10.1371/journal.pone.0013843 (PMC2973945; doi:10.1371/journal.pone.0013843)
Supplement: Table S1 — Top 10 annotated clusters from DAVID Bioinformatics Site. (0.12 MB DOC) [file pone.0013843.s006.doc]

**Table S1. Top 10 annotated clusters from DAVID Bioinformatics Site.**

| **MAUD hits** |  |  |  |
| --- | --- | --- | --- |
|  | ^**^Fold | *P* Value | ^***^Corrected |
| Cluster 1: **Development** ^*^Score: 5.2 |  |  |  |
| GO:0048731~system development | 1.4 | 1.10E-08 | 3.60E-05 |
|  |  |  |  |
| Cluster 2: **Ion Channels** Score: 4.3 |  |  |  |
| GO:0015075~ion transmembrane transporter activity | 1.7 | 3.30E-08 | 1.30E-05 |
| SP: voltage-gated channel | 2.6 | 2.30E-07 | 2.50E-05 |
|  |  |  |  |
| Cluster 3/10: **Extracellular/Membrane Proteins**  Score:4.3/3.0 |  |  |  |
| SP: glycoprotein | 1.3 | 9.70E-09 | 2.70E-06 |
| SP: disulfide bond | 1.3 | 9.70E-06 | 5.30E-04 |
| GO:0005886~plasma membrane | 1.3 | 6.10E-09 | 2.90E-06 |
|  |  |  |  |
| Cluster 4: **Guanyl-nucleotide exchange** Score: 3.9 |  |  |  |
| SP: guanine-nucleotide releasing factor | 3.3 | 1.10E-09 | 5.90E-07 |
| GO:0005085~guanyl-nucleotide exchange factor activity | 2.7 | 3.30E-09 | 3.70E-06 |
|  |  |  |  |
| Cluster 5: **Calmodulin Binding** Score: 3.8 |  |  |  |
| GO:0005516~calmodulin binding | 2.3 | 5.10E-05 | 2.50E-03 |
| SP: calmodulin-binding | 2.4 | 1.00E-04 | 4.00E-03 |
|  |  |  |  |
| Cluster 6/7: **Actin/Cytoskeleton** Score: 3.8/3.8 |  |  |  |
| GO:0030029~actin filament-based process | 2.3 | 1.90E-06 | 9.40E-04 |
| GO:0030036~actin cytoskeleton organization | 2.2 | 6.60E-06 | 2.50E-03 |
| GO:0007010~cytoskeleton organization | 1.8 | 2.80E-05 | 6.80E-03 |
| GO:0008092~cytoskeletal protein binding | 1.7 | 2.60E-05 | 1.60E-03 |
|  |  |  |  |
| Cluster 8: **Blood Vessel Development** Score: 3.2 |  |  |  |
| GO:0048646~anatomical structure formation/morphogenesis | 1.6 | 3.20E-04 | 5.30E-02 |
| GO:0001525~angiogenesis | 2 | 4.50E-04 | 6.00E-02 |
|  |  |  |  |
| Cluster 9: **Src Homology-3 Domain** Score: 3.2 |  |  |  |
| IPR001452:Src homology-3 domain | 1.9 | 1.00E-04 | 4.00E-02 |
| SP: sh3 domain | 1.9 | 1.60E-04 | 5.50E-03 |
|  |  |  |  |
|  |  |  |  |
|  |  |  |  |

| **MAUD hits with bivalent chromatin** |  |  |  |
| --- | --- | --- | --- |
|  | Fold | P Value | Corrected |
| Cluster 1: **Development**  Score: 11.0 |  |  |  |
| GO:0048731~system development | 2.4 | 6.40E-19 | 1.20E-15 |
|  |  |  |  |
| Cluster 2: **Ion Channels** Score: 6.4 |  |  |  |
| SP: voltage-gated channel | 7.4 | 1.60E-12 | 2.30E-10 |
| GO:0022843~voltage-gated cation channel activity | 7.2 | 9.40E-12 | 2.40E-09 |
|  |  |  |  |
| Cluster 3/10: **Membrane/Extracellular Proteins**  Score: 5.7/3.7 | |  |  |
| SP:glycoprotein | 1.8 | 2.70E-14 | 7.90E-12 |
| GO:0005886~plasma membrane | 1.7 | 2.50E-08 | 1.50E-06 |
| SP: signal | 1.6 | 1.00E-06 | 3.00E-05 |
| SP: disulfide bond | 1.6 | 2.10E-05 | 5.20E-04 |
| SP: Secreted | 1.5 | 4.60E-03 | 4.20E-02 |
|  |  |  |  |
| Cluster 4: **Neural development** Score: 4.6 |  |  |  |
| GO:0007399~nervous system development | 2.6 | 1.40E-09 | 3.30E-07 |
| GO:0022008~neurogenesis | 2.4 | 3.40E-05 | 1.60E-03 |
|  |  |  |  |
| Cluster 5: **Embryonic Development** Score: 4.5 |  |  |  |
| GO:0009790~embryonic development | 2.5 | 2.50E-07 | 2.50E-05 |
| GO:0048568~embryonic organ development | 3.8 | 6.40E-07 | 5.80E-05 |
|  |  |  |  |
| Cluster 6: **Sensory/Eye Development** Score: 4.2 |  |  |  |
| GO:0007423~sensory organ development | 3.9 | 2.30E-07 | 2.60E-05 |
| GO:0043010~camera-type eye development | 3.9 | 4.70E-04 | 1.10E-02 |
|  |  |  |  |
| Cluster 7: **Membrane Potential**  Score: 4.1 |  |  |  |
| GO:0042391~regulation of membrane potential | 5.1 | 3.30E-06 | 2.80E-04 |
| GO:0050801~ion homeostasis | 3.1 | 3.00E-05 | 1.50E-03 |
|  |  |  |  |
| Cluster 8: **Blood Vessel Development** Score: 3.8 |  |  |  |
| GO:0048646~anatomical structure formation/morphogenesis | 2.8 | 1.30E-05 | 7.40E-04 |
| GO:0001525~angiogenesis | 3.5 | 1.20E-03 | 2.30E-02 |
|  |  |  |  |
| Cluster 9: **Intercellular Communication** Score: 3.7 |  |  |  |
| GO:0007267~cell-cell signaling | 3.2 | 9.80E-06 | 6.30E-04 |
| GO:0019226~transmission of nerve impulse | 3.1 | 1.90E-04 | 6.30E-03 |

__________________________________________________________________________________

We used the DAVID Bioinformatics Website version 6.7 (david.abcc.ncifcrf.gov) to compare gene cluster enrichment in MAUD hits *vs.* MAUD hits that also show developmental bivalent histone modification. For ease of comparison, we assigned names to the clusters based on the annotation of the clustered terms. Where possible, identical terms in the two groups are shown for direct comparison. Where closely related clusters are combined, ranks and scores for each of the original clusters are shown. DAVID’s clustering algorithm was set to "medium" stringency. GO indicates annotation terms from the Gene Ontology Consortium. SP indicates Swiss Protein/Protein Information Resource keywords.

^*^Clusters are ordered by DAVID Bioinformatics using a score that is the mean of the negative log of the *P* values for each term in the cluster. Since only the top 1-3 terms for each cluster are shown here, the score cannot be calculated from the data in this table.

^**^Fold enrichment. ObsSp/((Tsp/T)*(ObsT)) where T is the total number of genes in the background list, Tsp is the number of genes having a specific annotation term in the background list, ObsT is the total number of genes in the data list (e.g., 2,337 for MAUD hits) and ObsSp is the number of genes having a specific annotation term in the data list.

^***^The corrected *p* values for multiple comparisons were obtained by the Benjamini method.
