# Supplementary material for: Dual DNA Methylation Patterns in the CNS Reveal Developmentally Poised Chromatin and Monoallelic Expression of Critical Genes
Source: PLoS One. 2010 Nov 4;5(11):e13843. doi: 10.1371/journal.pone.0013843 (PMC2973945; doi:10.1371/journal.pone.0013843)
Supplement: Dataset S2 — List of primers and genes assayed. (0.07 MB PDF) [file pone.0013843.s008.pdf]

## Dataset S2. List of primers and genes assayed.

Primers were designed with the aid of Primer3 (http://frodo.wi.mit.edu/primer3).
Except as noted, reverse transcription and PCR were carried out as previously described (Wang et al., 2007), with 1.5 mM $MgCl_{2.}$

## I. Primers for SNP detection in RNA

MAUD hits are underlined, and genes showing monoallelic expression are marked by an asterisk.

| Gene | Accession Number | Primer | Sequence | RT-PCR product | SNPs | SNP location | Annealing Temp. ( ° C) |
|---|---|---|---|---|---|---|---|
| *Abcd3* | NM_008991 | 803 | 5-GTCTTCCGAAAACTGGTGGA | 478 | G vs A | 204 | 59 |
| | | 804 | 5-TGTCACCATTTGGTGTTGCT | | | | |
| *Acsbg1* | BC057322 | 897 | 5-CATGTCCAGCCCCTACAACT | 641 | T vs C | 88 | 60 |
| | | 898 | 5-GTCCCAGTTCTCCACCTGAA | | | | |
| <u>*Acsl6*</u> | AK039380 | 1049 | 5-CGCCAGAGAAGATCGAGAAC | 521 | T vs C | 172 | 60 |
| | | 1050 | 5-GTCAGGAAAGGCTCAGTTGC | | | | |
| <u>*Adcy9*</u> | NM_009624 | 1051 | 5-GACGAGCCTAAGACCAGCAC | 406 | G vs C | 147 | 59 |
| | | 1052 | 5-CATCCAGAAGGGAGCTGAAG | | | | |
| *Adora1* | NM_001039510 | 825 | 5-CATTGGGCCACAGACCTACT | 419 | C vs T | 178 | 59 |
| | | 826 | 5-GCTTGCGGATCAGGTAGAAG | | | | |
| *Amph* | NM_175007 | 519 | 5-GGATTCTCAGGCAACTCTGC | 585 | C vs T | 290 | 60 |
| | | 520 | 5-TTGGTGGGAGGAGAATCAAG | | | | |
| <u>*Arpp21*</u> | NM_028755 | 777 | 5-CACAACGTGGATCAAACTGG | 506 | A vs C | 279 | 60 |
| | | 778 | 5-GTCATGGTGGGCTTCAGATT | | | | |
| <u>*Atrn*</u> | AF116897 | 901 | 5-TAGTTTGCTGCAGTGGATGC | 559 | C vs T | 172 | 60 |
| | | 902 | 5-GCAAGCTGGACAGTGAATGA | | | | |
| <u>*Atxn10*</u> | NM_016843 | 1347 | 5-AAGCTAGTGGGTGAGGAGCA | 446 | G vs A | 375 | 60 |
| | | 1348 | 5-ATCAGAGGAATGCCATCCAG | | | | |
| *Bmp6* | NM_007556 | 521 | 5-AAAGGCTACGCTGCCAACTA | 417 | C vs T | 285 | 60 |
| | | 522 | 5-CATAAAAGCCTCCGTGGGTA | | | | |
| <u>*Cacnb4*</u> | NM_146123 | 811 | 5-TTGGTAACAGATGGCACAGC | 507 | B6 gap, JF1 TT | 368 | 60 |
| | | 812 | 5-CACTAAGCGGAGGGAGAACA | | | | |
| <u>*Camk2a**</u> | NM_177407 | 837 | 5-CTGAACCCTCACATCCACCT | 506 | T vs C | 75 | 59 |

| | | | | | | | |
|---|---|---|---|---|---|---|---|
| | | 838 | 5-AGGCCAACAACAGTGGGTAG | | | | |
| <u>*Camk2b*</u> | BC080273 | 1059 | 5-ACTTTGAGGCCTATGCGAAA | 432 | G vs A | 185 | 60 |
| | | 1060 | 5-AGACACAAACATGCGACAGG | | | | |
| <u>*Camkk2*</u> | AK160407 | 1061 | 5-CATTACCGGTTTGCAGGACT | 643 | T vs C | 529 | 59 |
| | | 1062 | 5-GAAGATCTTGCGGGTCTCTG | | | | |
| <u>*Ccbl1*</u> | BC016206 | 1063 | 5-CAGCATGATGTCCTGTGCTT | 624 | G vs A | 462 | 59 |
| | | 1064 | 5-ATCCATGGCCTGGAGTGTAG | | | | |
| *Cerk* | NM_145475 | 747 | 5-TCTGCAAGGACAGACCCTCT | 388 | T vs C | 59 | 59 |
| | | 748 | 5-CAAGTGCCATTTGCTGAGAA | | | | |
| *Chgb* | NM_007694 | 1065 | 5-AGCTAGGACCGGAAGAAAGC | 523 | T vs C | 331 | 60 |
| | | 1066 | 5-GCAGCCAAGTTCTCCAGTTC | | | | |
| *Chn2* | NM_023543 | 1067 | 5-CCTCACACACAACGACAACC | 547 | G vs A | 121 | 60 |
| | | 1068 | 5-CTCATCCGCATTGGAGATTT | | | | |
| <u>*Cln8*</u> | BC021625 | 911 | 5-CCTGGGACAGCGAGTCTAAG | 602 | C vs T | 124 | 60 |
| | | 912 | 5-TGGTGGACAACCAGAAACA | | | | |
| *Cnr1* | BC079564 | 1075 | 5-CTGCAAGAAGCTGCAATCTG | 450 | T vs C | 151 | 59 |
| | | 1076 | 5-TGTCTCAGGTCCTTGCTCCT | | | | |
| *Coch* | NM_007728 | 607 | 5-GTGATGCCATCGCCTTTACT | 604 | C vs T | 314 | 60 |
| | | 608 | 5-TGTAAGCGCCAGGTTCTTTT | | | | |
| *Comt* | NM_007744 | 583 | 5-TGCAGTGATTCGGGAGTACA | 585 | C vs T | 67 | 60 |
| | | 584 | 5-GGGGGTCAGAGTGAGTGTGT | | | | |
| *Cplx2* | NM_009946 | 1077 | 5-CTCATTTCCCAAAGGCAGAC | 422 | CAG vs gap | 88 | 59 |
| | | 1078 | 5-CTCTTCCTCCTCCTCCTCGT | | | | |
| <u>*Cry1*</u> | NM_007771 | 1081 | 5-TAAGAGGCTTCCCTGCAAAA | 419 | G vs A | 200 | 59 |
| | | 1082 | 5-GCTTCTCCCTTGCTTGAGTG | | | | |
| <u>*Cspg4**</u> | NM_139001 | 917 | 5-TGATTCCTTCTCCCTGGATG | 415 | T vs C | 115 | 59 |
| | | 918 | 5-AAGGATGGTGATCGTGAAGG | | | | |
| *Ctnnb1* | BC048153 | 921 | 5-GGTGGACTGCAGAAAATGGT | 411 | T vs C | 171 | 60 |
| | | 922 | 5-TATCATCGGAACCCAGAAGC | | | | |
| *Cyfip2* | NM_133769 | 775 | 5-CACAACAGGATCACCCAGTG | 436 | G vs A | 120 | 59 |
| | | 776 | 5-CAGAGATGAAGCGGATGTGA | | | | |
| <u>*Dctn1*</u> | BC066061 | 1087 | 5-AGAGGCAAAGGAACGCTACA | 472 | A vs G | 334 | 60 |
| | | 1088 | 5-GGTCAGCATCTCCACCATCT | | | | |
| <u>*Dlgap1*</u> | NM_027712 | 1469 | 5-TGCTCACGTCACCAAAGTTC | 304 | G vs A | 152 | 60 |
| | | 1470 | 5-CTGCTCTGGATGGTCCTCAC | | | | |
| *Dpp10* | NM_199021 | 595 | 5-TCATTTCCAGCATTCAGCAG | 425 | G vs A | 174 | 60 |
| | | 596 | 5-AACCCTTGCAACTTCCCTTT | | | | |
| *Drd1a* | NM_010076 | 525 | 5-GCTCCTGATGGAACACCATT | 561 | C vs T | 100 | 60 |
| | | 526 | 5-CTGCCTTCGGAGTCATCTTC | | | | |

| | | | | | | | |
|---|---|---|---|---|---|---|---|
| *Dusp22* | NM_134068 | 527 | 5-GGCCTGTACATTGGCAACTT | 449 | T vs C | 87 | 60 |
| | | 528 | 5-CGCAAAGGGTTCTCTCCATA | | | | |
| *Elavl4* | NM_001038698 | 847 | 5-CCAAAGGATGCAGAGAAAGC | 460 | A vs G | 84 | 59 |
| | | 848 | 5-GCAAATTGTCCAGCCTGAAT | | | | |
| *<u>Elmo1</u>* | NM_080288 | 531 | 5-AGCGCTTACAACCAAGCCTA | 564 | C vs T | 298 | 60 |
| | | 532 | 5-ATCATGTCCTTCCCAAGCAG | | | | |
| *<u>Elmo2</u>* | NM_207705 | 849 | 5-CAGATGGGCCTCAGCTCTAC | 565 | G vs A | 56 | 59 |
| | | 850 | 5-AGCGCGTTAATCAGAGCAAT | | | | |
| *<u>Enpp2</u>* | BC058759 | 927 | 5-GTTCCGAGGATGAGTCGAAG | 558 | T vs C | 84 | 60 |
| | | 928 | 5-CGCAGATGAATGCACCTTTA | | | | |
| *Ephb1* | AK039530 | 929 | 5-GCACCCTCCAAGAGAGACTG | 589 | C vs T | 386 | 60 |
| | | 930 | 5-CTGCTCTCTCAGCTCCGACT | | | | |
| *Erbb3* | NM_010153 | 1091 | 5-GAGGCTTGTCTGGATTCTGC | 518 | G vs A | 165 | 60 |
| | | 1092 | 5-GAGGCTTGTCTGGATTCTGC | | | | |
| *<u>Fbxw11</u>* | AK168667 | 773 | 5-CCTCCGGGACAACTCTATCA | 568 | C vs T | 250 | 59 |
| | | 774 | 5-CAATTCTTCGTGCCCCTCTA | | | | |
| *<u>Fmod</u>* | NM_021355 | 933 | 5-GAGCCTCTGCTCATCCTTTG | 550 | A vs G | 129 | 60 |
| | | 934 | 5-CTGGTTTGGCTTTTGTGGAT | | | | |
| *<u>Foxf2</u>* | NM_010225 | 533 | 5-GCGCTTCACCTTACCTCAAG | 553 | C vs A | 200 | 60 |
| | | 534 | 5-CCTCCTCTGTCTGTCGCTCT | | | | |
| *<u>Fxyd6</u>* | BC042579 | 787 | 5-TCTGTTCCTGAGGAGCCCTA | 499 | A vs C | 190 | 59 |
| | | 788 | 5-CCAGGCAGAGAGACAGAACC | | | | |
| *<u>Fyn</u>* | NM_008054 | 937 | 5-TAATGGGCTGTGTGCAATGT | 556 | G vs T | 269 | 60 |
| | | 938 | 5-TAGGCACCTTTGGTGGTTTC | | | | |
| *Fzd3* | NM_021458 | 599 | 5-GAAGCAAAGCAGGGAGTGTC | 445 | G vs A | 287 | 60 |
| | | 600 | 5-ACCCCAGGGTTGATGTGATA | | | | |
| *Garnl1* | NM_001003719 | 643 | 5-AGTGCAGGGAGCAGTGATCT | 641 | A vs G | 241 | 60 |
| | | 644 | 5-TTGTCAGCTGAAATGCCAAG | | | | |
| *Gfap* | AK079577 | 941 | 5-GAGGCAGAAGCTCCAAGATG | 408 | G vs A | 61 | 60 |
| | | 942 | 5-CGGCGATAGTCGTTAGCTTC | | | | |
| *<u>Gfra2*</u>* | NM_008115 | 853 | 5-CCTGAACGACAACTGCAAGA | 347 | T vs C | 70 | 59 |
| | | 854 | 5-AGCTGGTGATTGTCCGGTAG | | | | |
| *<u>Gnal</u>* | AK158831 | 1099 | 5-TATCTCCCTAGCCTGCGTGT | 516 | C vs T | 76 | 60 |
| | | 1100 | 5-TGCAACTGTGGCTGAAAAAG | | | | |
| *Gnaz* | NM_010311 | 857 | 5-GCCTCTTTGACTCCATCTGC | 616 | T vs C | 98 | 59 |
| | | 858 | 5-TCAGGAAACCTTGACGCTCT | | | | |
| *Gprin1* | NM_012014 | 537 | 5-CTGGGTCCTTGAGGAATGAA | 602 | G vs A | 338 | 60 |
| | | 538 | 5-CACCGATACAGACCCACCTT | | | | |
| *<u>Grid1</u>* | NM_008166 | 1101 | 5-ACGCCTTTCTATGGGATGTG | 424 | A vs C | 220 | 60 |

| | | | | | | | |
|---|---|---|---|---|---|---|---|
| | | 1102 | 5-CGGTGTACCTGCTCCAGATT | | | | |
| *Grik4* | NM_175481 | 585 | 5-GAATGGACAGCCTTGTGGAT | 496 | A vs G | 400 | 60 |
| | | 586 | 5-ACCAGGGTGGTGTTGAAGAG | | | | |
| *Hrh1* | NM_012014 | 1103 | 5-GACCGCCATCATCAACTTCT | 532 | G vs A | 157 | 60 |
| | | 1104 | 5-TGGCTCTATGCTGGTGTCTG | | | | |
| *Igsf3** | NM_207205 | 801 | 5-CATCCAGATGGAGCAGGTG | 437 | C vs T | 187 | 59 |
| | | 802 | 5-GGACATTGTTGCTGGACTCA | | | | |
| *Il1f5* | NM_019451 | 809 | 5-GGGCCAATTCTGAAACTTGA | 430 | G vs A | 253 | 60 |
| | | 810 | 5-ATTTACCAGCACTGGGATGC | | | | |
| *Impa2* | BC011093 | 863 | 5-ACCTTGGCCCTCTGTTACCT | 432 | C vs T | 234 | 60 |
| | | 864 | 5-GGGTTTGGCTCTGATGAAAA | | | | |
| *Ipmk* | AK172246 | 947 | 5-TGCAAATCTTTTTGCTGTGC | 502 | G vs A | 279 | 60 |
| | | 948 | 5-GCAGATGGGCTTGAGAGTTC | | | | |
| *Itih3* | NM_008407 | 867 | 5-TTGACCACTCAGCAGTCCAG | 609 | A vs G | 70 | 60 |
| | | 868 | 5-TGTGGCATCTGGCTTTGTAG | | | | |
| *Kcnc4** | NM_145922 | 953 | 5-TGTATCCCAAGACGTGGTCA | 603 | A vs G | 185 | 59 |
| | | 954 | 5-ACTTCAGCGTGGGCATAGTT | | | | |
| *Kcnd3* | NM_019931 | 807 | 5-CCTTCTCTGTCCAGCCACTC | 588 | T vs C | 90 | 60 |
| | | 808 | 5-GCCCCAGTAAGATGCAATGT | | | | |
| *Kcnmb1* | NM_031169 | 1309 | 5-ATCTTGGCGGCTCAGAAGTA | 374 | C vs T | 158 | 60 |
| | | 1310 | 5-ATTTGGCCAAGGACAACATC | | | | |
| *Kif5c* | NM_008449 | 959 | 5-ACTGGAACAGGAGAGGCTCA | 580 | A vs G | 69 | 60 |
| | | 960 | 5-CGGAGATGATGCTGGGTAAT | | | | |
| *Lgi1** | NM_020278 | 871 | 5-CCTGTGAGGCTCATGTTTGA | 579 | T vs C | 473 | 59 |
| | | 872 | 5-CTCCATAGAGGCAGCACACA | | | | |
| *Mal* | BC006826 | 969 | 5-TTCAAGGCACAACAGTCTG | 619 | C vs T | 213 | 60 |
| | | 970 | 5-TGGCTGGGATAACCAAAGTC | | | | |
| *Map4k2/Rab8i* | NM_009006 | 971 | 5-CTTGGGTTCACCCTGTGACT | 621 | G vs A | 221 | 60 |
| | | 972 | 5-GAGGGATGAGGATGTCTGGA | | | | |
| *Mark1* | NM_145515 | 973 | 5-CTGAACAACAGCACCCTTCA | 388 | C vs T | 105 | 60 |
| | | 974 | 5-GGCTGCTGTCTCTTCCATTC | | | | |
| *Mrps25* | BC022953 | 981 | 5-CGCCAGGAAATTTGTGTTTT | 355 | G vs a | 267 | 60 |
| | | 982 | 5-CCCTGTCATCTCCTTGGGTA | | | | |
| *Mylip* | NM_153789 | 541 | 5-CCCTGAAGTCCTCAGACAGC | 597 | A vs C | 313 | 60 |
| | | 542 | 5-TTGCCACTTGACTGACGAAG | | | | |
| *Myo9b* | NM_015742 | 877 | 5-AAGAACCTGCTGGACTCGAA | 579 | T vs C | 343 | 60 |
| | | 878 | 5-GGCAGCATGCTTCATCTGTA | | | | |
| *Napb* | BC038362 | 983 | 5-GCCAACAAGTGTCTGCTGAA | 387 | A vs G | 126 | 60 |
| | | 984 | 5-CCCTTGGATGGACTTCTTG | | | | |

| | | | | | | | |
|---|---|---|---|---|---|---|---|
| *Nck2* | BC011071 | 1113 | 5-GGCTCCCGTGTCACAGTTAT | 590 | G vs A | 104 | 59 |
| | | 1114 | 5-TTGTTTCTCCCTGACGCTTT | | | | |
| *Ndst1* | NM_008306 | 749 | 5-AAGGCTTCCCTCTCTTCCTG | 431 | A vs T | 68 | 59 |
| | | 750 | 5-CTCCTTGCCCACAAAAATGT | | | | |
| *Nedd9* | NM_017464 | 543 | 5-CAACTGACCACCACCATCAG | 549 | G vs A | 321 | 60 |
| | | 544 | 5-GATGGCGTTGAGTAGGGAAA | | | | |
| *Nes* | AF076623 | 797 | 5-TCTCTTCCCCCTTGCCTAAT | 447 | A vs T | 224 | 60 |
| | | 798 | 5-CCTCTGTGGCTGCTTCTTTC | | | | |
| *Nfatc2* | NM_001037177 | 879 | 5-TCTGCTGTTCTCATGGATGC | 574 | T vs C | 261 | 60 |
| | | 880 | 5-TCTCATAGCTCGTGGTGGTG | | | | |
| *Nos2* | BC062378 | 989 | 5-GTGGTGACAAGCACATTTGG | 487 | T vs G | 75 | 60 |
| | | 990 | 5-GGCTGGACTTTTCACTCTGC | | | | |
| *Npas3* | NM_013780 | 611 | 5-CAGTCACCTGGACTTGCTGA | 543 | T vs C | 293 | 60 |
| | | 612 | 5-ACGTAGCGCTCCACTTTGAT | | | | |
| *Nr1h3* | NM_013839 | 991 | 5-TACAACCGGGAAGACTTTGC | 465 | A vs T | 255 | 60 |
| | | 992 | 5-CCCTTCTCAGTCTGCTCCAC | | | | |
| *Nr4a1* | NM_010444 | 1351 | 5-GAAATTGTTCCCACCACCAG | 469 | C vs T | 145 | 60 |
| | | 1352 | 5-CGGATGAGGGAAGTGAGAAG | | | | |
| *Nrg2* | AY227025 | 579 | 5-ATCATCATCTCCGGCAGAAC | 523 | C vs T | 227 | 60 |
| | | 580 | 5-GCCAGCGAGTAGTGGAAGTC | | | | |
| *Nrxn1* | BC047146 | 1141 | 5-GCAAAGGTGGTGGACAGATT | 561 | A vs C | 134 | 60 |
| | | 1142 | 5-CATCGTTCTCTGCTGCCATA | | | | |
| *Nrxn2* | NM_020253 | 995 | 5-ATACATGGCCTCCCAATGAC | 257 | A vs T | 65 | 59 |
| | | 996 | 5-GCCACCACTTCGAGTAAAGC | | | | |
| *Nt5m* | BC020084 | 997 | 5-ACTGTGATCTCAGCCGACCT | 513 | T vs C | 304 | 60 |
| | | 998 | 5-CCTGGATCAGGGTACTTCCA | | | | |
| *Per2* | AK159847 | 1117 | 5-TTGACGCGTTTTGACTTCAG | 403 | A vs G | 99 | 60 |
| | | 1118 | 5-TTGACGCGTTTTGACTTCAG | | | | |
| *Pick1* | NM_008837 | 1119 | 5-CCTACAGAAGGATGCCCAGA | 567 | G vs A | 256 | 60 |
| | | 1120 | 5-GCGTCTGCAAACTTCACAAA | | | | |
| *Plcb1* | NM_019677 | 1121 | 5-CACCTACAGCGACTCCTCCA | 562 | A vs T | 144 | 60 |
| | | 1122 | 5-GGCCACTATCCCATCTACGA | | | | |
| *Pmp22* | NM_008885 | 1377 | 5-TCTTGTTGGGGATCCTGTTC | 400 | C vs T | 218 | 60 |
| | | 1378 | 5-ATGTAGGCGAAGCCATAGGA | | | | |
| *Ppp2r5d* | NM_009358 | 1005 | 5-CTTCCCCTTCAACCTCAACA | 547 | A vs G | 154 | 60 |
| | | 1006 | 5-CCCCAAAAACTTGCCATAGA | | | | |
| *Ptgds* | NM_008963 | 1123 | 5-TCCTTGCTTTGTCCACATTG | 434 | A vs G | 401 | 60 |
| | | 1124 | 5-CATAGTTGGCCTCCACCACT | | | | |
| *Rabggta* | BC012214 | 765 | 5-GAGCAGCCTACTTGGACGAC | 454 | C vs T | 408 | 59 |

| | | | | | | | |
|---|---|---|---|---|---|---|---|
| | | 766 | 5-TCCTAGGTGAGGATGCTGCT | | | | |
| *Rapgef4* | AK220281 | 813 | 5-GGCGTACCAGATGACAACCT | 624 | C vs T | 289 | 60 |
| | | 814 | 5-CGGACATCCTGATGGTTCTT | | | | |
| *Rarb* | NM_011243 | 727 | 5-CAAGGCAAAAACACTGTCCA | 266 | T vs C | 153 | 59 |
| | | 728 | 5-GCCGACACTTGAGTCTGTCA | | | | |
| *Rbms3* | AK162457 | 783 | 5-GCCAGGGACAGTGGATTAAG | 448 | C vs T | 425 | 59 |
| | | 784 | 5-AAGGACGCCACTGCTTTCT | | | | |
| *Rhpn1* | BC052010 | 1009 | 5-GCGAGGATGACTTCTTCGAG | 489 | A vs G | 144 | 60 |
| | | 1010 | 5-GTTTATTCTTGCGGCTCCAG | | | | |
| *Ror2* | NM_013846 | 567 | 5-GGAATATAAGGGGCCTGGAG | 306 | T vs A | 151 | 60 |
| | | 568 | 5-GCGGTGAAGATGTAGCCTGT | | | | |
| *Rorb* | AK134790 | 1013 | 5-TGAGAATGTGTCGTGCCTTC | 639 | G vs A | 104 | 60 |
| | | 1014 | 5-CCCCACTCAGGTGCATATTT | | | | |
| *S100b* | BC061178 | 1125 | 5-AGAGGAGCACAGCCACACTT | 415 | T vs C | 283 | 60 |
| | | 1126 | 5-GACTCTCAGCCTCACCAAGG | | | | |
| *Serpinb9* | BC029900 | 1337 | 5-TCCAAGGCCTTCATGGATTA | 440 | T vs A | 73 | 60 |
| | | 1338 | 5-TGCTCATGGTGAGGTCAGAG | | | | |
| *Sgip1* | AK014022 | 1017 | 5-GGCATTCAGTCCACACCTCT | 475 | T vs A | 105 | 60 |
| | | 1018 | 5-TGGATCTTCCAAATCCTTGC | | | | |
| *Sh3tc2* | NM_172628 | 753 | 5-GCAGTGGTTCATTGGGAAGT | 490 | T vs C | 77 | 59 |
| | | 754 | 5-GTGCTCCACATCATCCTCCT | | | | |
| *Slc12a7* | AK052910 | 1339 | 5-GGAAAACAGCCCTTTCATCA | 464 | G vs A | 93 | 60 |
| | | 1340 | 5-CAACAGCACCTCCAAACTCA | | | | |
| *Slc1a2* | NM_001077514 | 885 | 5-GGCAATCCCAAACTCAAGAA | 480 | G vs A | 126 | 59 |
| | | 886 | 5-GTCCTTGATGGCGATGATCT | | | | |
| *Slc1a3* | BC066154 | 1019 | 5-TCTGACTCCTGCCCTGACTT | 411 | T vs C | 303 | 60 |
| | | 1020 | 5-TGGGAAGTGTACCCCAGAAG | | | | |
| *Slc6a1** | NM_178703 | 795 | 5-GATCGATTAGGCTGCAAAGC | 571 | T vs C | 271 | 59 |
| | | 796 | 5-GGTCGTGGTGAAGGAGTTGT | | | | |
| *Slc20a1* | NM_015747 | 1021 | 5-GGTTTCCGTGCCGTAGTTTA | 532 | G vs A | 443 | 60 |
| | | 1022 | 5-CGGAAGCTTCAAAAACGAAG | | | | |
| *Smad2* | NM_010754 | 1023 | 5-CTGGCTCAGTCTGTCAACCA | 536 | C vs T | 260 | 60 |
| | | 1024 | 5-TGCAATACTGGGTCTGGACA | | | | |
| *Sncaip* | NM_026408 | 769 | 5-CAGGAGCCAAAACAGAGGAG | 425 | T vs C | 57 | 60 |
| | | 770 | 5-CCCATAGCAACCTGCGTAAT-3 | | | | |
| *Snta1* | NM_009228 | 1027 | 5-GCACGAGTCTCCTTTTCCTG | 602 | A vs C | 250 | 60 |
| | | 1028 | 5-GATGAAGGAGGGGAGAGAGG | | | | |
| *Sparc* | NM_009242 | 1029 | 5-AAACATGGCAAGGTGTGTGA | 468 | T vs C | 252 | 60 |
| | | 1030 | 5-CCAGTGGACAGGGAAGATGT | | | | |

| | | | | | | | |
|---|---|---|---|---|---|---|---|
| *Spry4* | NM_011898 | 1131 | 5-GTGAGGCCTGTGGAAAGTGT | 563 | C vs T | 483 | 60 |
| | | 1132 | 5-ATCCTTTTGGGGACTCAAGG | | | | |
| *Srpk1* | NM_016795 | 1031 | 5-GGGGAGGATTACACACGAGA | 550 | G vs A | 63 | 60 |
| | | 1032 | 5-GGGTGAGGCTGTTCACACTT | | | | |
| *Stx1a* | NM_016801 | 1133 | 5-GAACAAAGTTCGCTCCAAGC | 576 | G vs A | 235 | 60 |
| | | 1134 | 5-ATGATGCCCAGAATCACACA | | | | |
| *Stxbp6* | BC024598 | 645 | 5-CAATATGGTGGCATCAGGTG | 555 | C vs T | 58 | 60 |
| | | 646 | 5-AATGTGGGTGGAGGTCAGAG | | | | |
| *Sulf2* | NM_028072 | 1033 | 5-AATGCCCAGGAGGAGAACTT | 543 | T vs C | 165 | 60 |
| | | 1034 | 5-GTAGCACCGATGGGTCACTT | | | | |
| *Sult4a1* | NM_013873 | 1135 | 5-ATGAAATCGGCCTGATGAAC | 430 | T vs C | 278 | 60 |
| | | 1136 | 5-CAGGACACACCCAGGAATCT | | | | |
| *Syt12* | NM_134164 | 1035 | 5-GGCGAAACTAACAGCCAGAG | 613 | T vs C | 133 | 60 |
| | | 1036 | 5-CCCATGGTAAAGATGGGATG | | | | |
| *Tecta* | NM_009347 | 1137 | 5-TGACTGGAAGTGTGGCTCTG | 557 | G vs T | 349 | 60 |
| | | 1138 | 5-CTCCCTCTTCGCATAAGCAC | | | | |
| *Tnfaip8l2* | BC055879 | 891 | 5-CGGGTGATCAAAGACCTCAT | 647 | G vs T | 391 | 59 |
| | | 892 | 5-AGAAGCGGACCTGAGTTTCA | | | | |
| *Unc5a** | NM_153131 | 553 | 5-CCTTCAAGATCCCCTTCCTC | 497 | C vs A | 68 | 59 |
| | | 554 | 5-AGCGAGAACAGGTGGCTAAA | | | | |
| *Vangl1* | NM_177545 | 1043 | 5-CTTCATCCTGCTACCCCAAA | 589 | A vs G | 85 | 60 |
| | | 1044 | 5-GGACTGACCAGTGGCGTTAT | | | | |
| *Vmp** | NM_001087053 | 555 | 5-GCCTAACAGGTGGAGCTCAG | 641 | T vs C | 577 | 59 |
| | | 556 | 5-GAACCCACCAGAAGCAGGTA | | | | |
| *Wwc1* | NM_170779 | 1047 | 5-CGGTATGAAGAGGCGAAGAG | 400 | A vs G | 141 | 59 |
| | | 1048 | 5-CAGGGCTACTGCCTGAAGAC | | | | |

## II. Primers for SNP detection in genomic DNA

| Gene | Primer | Sequence |
|---|---|---|
| *Cspg4* | 1503 | 5-CGGGTTCTCTACCTGCACTC |
| | 1504 | 5-CTCCAGGAGAAGGTGCTGAG |
| *Camk2a* | 837 | 5-CTGAACCCTCACATCCACCT |
| | 1506 | 5-GTGGACGATCTGCCATTTG |
| *Gfra2* | 853 | 5-CCTGAACGACAACTGCAAGA |
| | 1510 | 5-CCTGACAGGAGCAGAAGAGC |

| | | |
|---|---|---|
| *Igsf3* | 1523 | 5-AACCCACACAGGATCTCAGG |
| | 1524 | 5-TGGAACCTCCATGTCACAGA |
| *Kcnc4* | 953 | 5-TGTATCCCAAGACGTGGTCA |
| | 1516 | 5-GGGCTGGTGTCACTGTAGGT |
| *Lgi1* | 871 | 5-CCTGTGAGGCTCATGTTTGA |
| | 872 | 5-CTCCATAGAGGCAGCACACA |
| *Slc6a1* | 1517 | 5-ACGTGGACTGGAGCTGACAT |
| | 1518 | 5-TCCCTCCCCTCAAGGTAGAC |
| *Unc5a* | 673 | 5-ACGAGGTTTGCTGAAATGCT |
| | 674 | 5-TGTCCAGGTGAAGTTTCTGG |
| *Vmp* | 685 | 5-GCAGCCTCTATCAGCCACTT |
| | 556 | 5-GAACCCACCAGAAGCAGGTA |

## III. Primers for real time PCR

PCR conditions were: 95° 3 min, 1 cycle; 95° 30 sec, 60° 30 sec, 70° 30 sec, cycles to completion.

| **Gene** | **Primer** | **Sequence** |
|---|---|---|
| *Cspg4* | see primers 917-918, above | |
| *Pgk1* | js141 | 5-AGCTGAGCCGGCCAAAATTGAT |
| | js142 | 5-GTAAAGGCCATTCCACCACCAA |
| *Nes* | js217 | 5-CTTGCAGACACCTGGAAGAAG |
| | js218 | 5-TATTAGGCAAGGGGGAAGAGA |
| *Dcx* | js239 | 5'TCCTTGGATGAGAATGAATGC |
| | js240 | 5-AGGGCTTGTGGGTGTAGAGAT |
